# Targeting proprotein convertase subtilisin/kexin type 9 (PCSK9): from bench to bedside

**DOI:** 10.1038/s41392-023-01690-3

**Published:** 2024-01-08

**Authors:** Xuhui Bao, Yongjun Liang, Hanman Chang, Tianji Cai, Baijie Feng, Konstantin Gordon, Yuekun Zhu, Hailian Shi, Yundong He, Liyi Xie

**Affiliations:** 1 Institute of Therapeutic Cancer Vaccines, Fudan University Pudong Medical Center, Shanghai, China; 2 Shanghai Key Laboratory of Regulatory Biology, School of Life Sciences, East China Normal University, Shanghai, China; 3 Department of Oncology, Fudan University Pudong Medical Center, Shanghai, China; 4 Center for Clinical Research, Fudan University Pudong Medical Center, Shanghai, China; 5 Clinical Research Center for Cell-based Immunotherapy, Fudan University, Shanghai, China; 6 Department of Pathology, Duke University Medical Center, Durham, NC USA; 7 Center for Medical Research and Innovation, Fudan University Pudong Medical Center, Shanghai, China; 8 Institute for Food Safety and Health, Illinois Institute of Technology, Chicago, IL USA; 9 Department of Sociology, University of Macau, Taipa, Macau China; 10 Medical Institute, Peoples’ Friendship University of Russia, Moscow, Russia; 11 A. Tsyb Medical Radiological Research Center, Obninsk, Russia; 12 Department of Colorectal Surgery, The First Affiliated Hospital of Harbin Medical University, Harbin, Heilongjiang China; 13 Shanghai Key Laboratory of Compound Chinese Medicines, Institute of Chinese Materia Medica, Shanghai University of Traditional Chinese Medicine, Zhangjiang Hi-tech Park, Shanghai, China; 14 Department of Radiation Oncology, Fudan University Shanghai Cancer Center, Shanghai, China; 15 Department of Oncology, Shanghai Medical College, Fudan University, Shanghai, China

**Keywords:** Translational research, Molecular medicine

## Abstract

Proprotein convertase subtilisin/kexin type 9 (PCSK9) has evolved as a pivotal enzyme in lipid metabolism and a revolutionary therapeutic target for hypercholesterolemia and its related cardiovascular diseases (CVD). This comprehensive review delineates the intricate roles and wide-ranging implications of PCSK9, extending beyond CVD to emphasize its significance in diverse physiological and pathological states, including liver diseases, infectious diseases, autoimmune disorders, and notably, cancer. Our exploration offers insights into the interaction between PCSK9 and low-density lipoprotein receptors (LDLRs), elucidating its substantial impact on cholesterol homeostasis and cardiovascular health. It also details the evolution of PCSK9-targeted therapies, translating foundational bench discoveries into bedside applications for optimized patient care. The advent and clinical approval of innovative PCSK9 inhibitory therapies (PCSK9-iTs), including three monoclonal antibodies (Evolocumab, Alirocumab, and Tafolecimab) and one small interfering RNA (siRNA, Inclisiran), have marked a significant breakthrough in cardiovascular medicine. These therapies have demonstrated unparalleled efficacy in mitigating hypercholesterolemia, reducing cardiovascular risks, and have showcased profound value in clinical applications, offering novel therapeutic avenues and a promising future in personalized medicine for cardiovascular disorders. Furthermore, emerging research, inclusive of our findings, unveils PCSK9’s potential role as a pivotal indicator for cancer prognosis and its prospective application as a transformative target for cancer treatment. This review also highlights PCSK9’s aberrant expression in various cancer forms, its association with cancer prognosis, and its crucial roles in carcinogenesis and cancer immunity. In conclusion, this synthesized review integrates existing knowledge and novel insights on PCSK9, providing a holistic perspective on its transformative impact in reshaping therapeutic paradigms across various disorders. It emphasizes the clinical value and effect of PCSK9-iT, underscoring its potential in advancing the landscape of biomedical research and its capabilities in heralding new eras in personalized medicine.

## Background

### The discovery of proprotein convertase subtilisin/kexin type 9 (PCSK9) and its structure

The understanding that polypeptide hormones, including melanotropins, β-endorphin, and insulin, derive from larger and predominantly inactive precursor proteins through a series of cleavages at basic amino acids (aa) pairs, has been established since the 1960s.^1–6^ This principle of restricted proteolysis was later applied to a variety of secretory proteins and even pathogens, with proteolytic cleavages occurring at single or paired basic residues within a defined motif.^7^ In humans, over 560 proteases have been identified, among which proprotein convertases are a small family of serine endoproteases that recognize paired or multiple basic clusters or hydrophobic motifs to process a multitude of protein precursors (proproteins).^8^ This family comprises seven initial members of basic aa-specific serine proteases, associated with subtilisin/kexin, with their genes predominantly termed proprotein convertases subtilisin/kexin (PCSKs), including PC1 (gene *PCSK1*), PC2 (gene *PCSK2*), Furin (gene *Fur*), PC4 (gene *PCSK4*), PC5 (gene *PCSK5*), PACE4 (gene *PCSK6*), and PC7 (gene *PCSK7*),^7,9^ with the eighth member, subtilisin-kexin isozyme 1 (SKI-1), identified in 1999,^10^ and PCSK9, the ninth member, discovered in 2003.^11^ Initially, PCSK9 was referred to as neural apoptosis-regulated convertase-1 (NARC1), achieved by amplifying mRNAs that potentially encoded a SKI-1/S1P equivalent.^11^ The cDNA of PCSK9, initially discovered in projects researching apoptosis in cerebellar neurons and secretory proteins, was eventually identified in human, mouse, and rat libraries. PCSK9, a member of the proteinase K family of subtilases, was thus named due to its solubility and its role in gene expression related to apoptosis.^12^ The mRNA of human PCSK9, which is 3,710 base pairs (bp) in length across 12 exons, encodes a protein with 692 aa. Subsequent detailed examination determined that the primary production sites of PCSK9 in humans, mice, and rats were the liver and small intestine.^11^ The three-dimensional (3D) structures of PCSK9 show three distinct domains: the prodomain (aa 31–152), the catalytic domain (aa 153–421), and the C-terminal Cys/His-rich domain (CHRD; aa 453–692), each playing a significant role in managing PCSK9’s biological functions and its trafficking inside cells.^13,14^

**Fig. 1:**
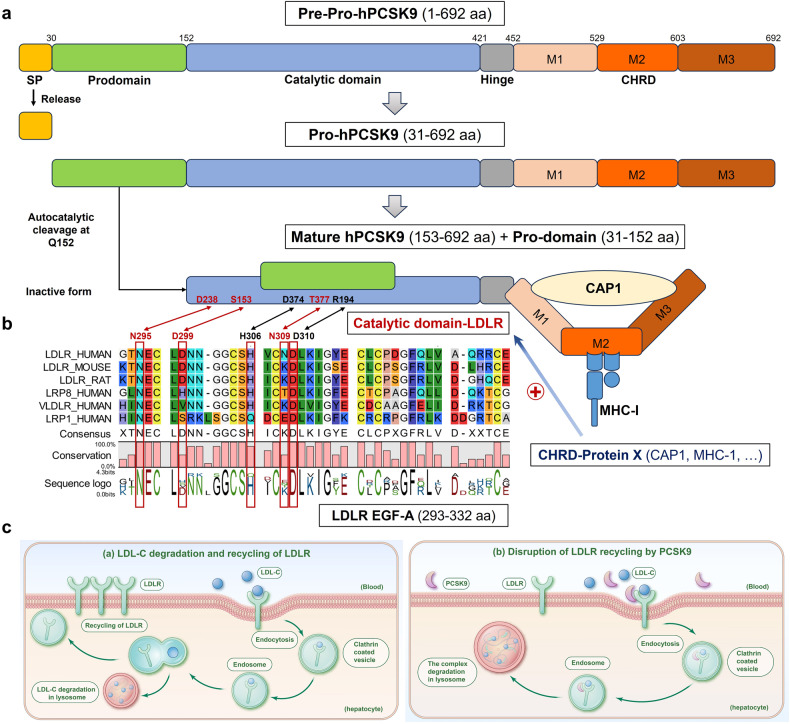
The main structure and function of PCSK9. **a** PCSK9 comprises a signal peptide (SP, aa 1–30), a prodomain (aa 31–152), a catalytic domain (aa 153–421) with a hinge (aa 422–452), and a Cysteine-Histidine rich C-terminal domain (CHRD, aa 453–692) that can be further divided into three modules, M1 (aa 453–529), M2 (aa 530–603), and M3 (aa 604–692). In ER, proPCSK9 undergoes autocatalytic cleavage at Q152. The prodomain is then separated from the mature PCSK9, but remains associated with the catalytic domain, inhibiting the protease activity of the mature PCSK9. **b** There are five residues directly involved in the avidity of PCSK9:LDLR interface including S153:D299, R194:D310, D238:N295, D374:H306, and T377:N309, in which the hydrogen bonds between D238 and N295, T377 and N309, and a salt bridge between S153 and D299 contribute to the specificity of PCSK9 binding to the epidermal growth factor (EGF)-A domain of low-density lipoprotein receptor (LDLR) instead of other EGF-like domains. Primary sequence alignments of EGF-A domain from selected species (human, mouse, and rat) and human LDLR family members (LDLR related protein 8 [LRP8]/apolipoproteinE receptor 2 [ApoER2], very low-density lipoprotein receptor [VLDLR], and LRP1) were performed using the CLC workbench. Furthermore, cyclase associated actin cytoskeleton regulatory protein 1 (CAP1) and major histocompatibility complex class 1 (MHC-1) (e.g., human leukocyte antigen [HLA]-C) may be two strong candidates for “protein X” that can promote the degradation of PCSK9-LDLR complex in acidic cytosolic compartments. **c** (a) LDLRs are crucial in controlling levels of LDL cholesterol (LDL-C) in the blood by managing their removal from circulation. LDLRs bind to LDL-C and the resulting complexes are internalized into hepatocytes through endocytosis into clathrin-coated vesicles that can be subsequently fused with endosomes, whose acidic environment leads to the dissociation of the LDL-C particles to be transported to lysosomes to degrade into lipids and amino acids, while LDLRs can recycle back to the surface of the hepatocytes to transport and clear additional LDL-C from the circulation. (b) When PCSK9 is secreted from hepatocytes and binds to LDLRs on the cell surface, LDLR recycling to the cell surface is impeded. Due to a conformational change in LDLR caused by PCSK9, LDLR cannot get out of the endosome to recycle back to the cell surface. Instead, the PCSK9-LDLR-LDL-C complex traffics to the lysosome for degradation. By promoting LDLR degradation, PCSK9 decreases LDLR levels at the cell surface, increasing serum LDL-C. Panels were illustrated by Adobe Illustrator and Microsoft PowerPoint

The prodomain, found between aa 31 and 152, is cleaved following the signal peptide (SP). Following this, the precursor PCSK9, also known as proPCSK9, performs an autocatalytic cleavage at the FAQ152/SIPK site, a process that begins relatively early in the endoplasmic reticulum (ER).^15,16^ Distinctively, PCSK9 preserves its connection to the prodomain post secretion, given the indispensability of the prodomain and its cleavage for PCSK9’s leaving from the ER.^11,17,18^ PCSK9 variants that obstruct this ER exit, such as Q152H,^19,20^ prevent PCSK9 secretion, leading to hypocholesterolemia and a loss-of-function (LOF) mutation.^21^ Over 40 gain-of-function (GOF) or LOF variants of PCSK9 have been determined in this sequence.^22^ These include the common LOF R46L variant linked with protection against heart disease, Tyr38-sulphation variant, and Ser47-phosphorylation variant.^22–26^ The catalytic domain, spanning aa 153–421, is vital in PCSK9’s degradation of low-density lipoprotein receptor (LDLR), which will be discussed more thoroughly in the following section. Within the enzymatic domain, three PCSK9 LOF variations, namely R215H, F216L, and R218S, have been detected. These findings led to the discovery that Furin has the ability to deactivate PCSK9 via cleavage at RFHR218 ↓ .^12,25,27–29^ Out of all PCSK9 GOF variants, the D374Y variant stands as the most potent,^30^ exhibiting an LDLR-binding affinity that is 10 to 20 times greater, as well as a strong resistance to Furin cleavage.^13,25,31^ Additionally, the PCSK9 enzymatic domain is followed by a rather disorganized hinge (aa 422–452), succeeded by a highly structured C-terminal 240-aa CHRD. This CHRD consists of three successive repeats, compactly organized into similar structural modules identified as M1 (aa 453–529), M2 (aa 530–603), and M3 (aa 604–692) (Fig. 1a). Each of these is characterized by their β-sheet structures. Intriguingly, out of the 14 histidine residues in the CHRD, nine are located within the M2 module, hinting at a potential pH-dependent function, especially under the acidic conditions inside cytosolic endosomes.^13,14^ The modules M1, M2, and M3 bear a structural resemblance to resistin, a secreted small protein to modulate mammalian glucose metabolism.^32^ Circulating levels of resistin have been associated with atherosclerosis, cardiovascular diseases (CVD), inflammation, and cancer.^33^ The majority of PCSK9 variations found within the CHRD occur within the M1 and M3 modules, whereas fewer PCSK9 genetic variations but greater structural flexibility is observed in the hinge region and M2 module.^13,14^ For instance, we discerned that the M2 module was instrumental in the binding of mouse PCSK9 and histocompatibility 2 (H2)-K1 protein^34^ (Fig. 1b).

Interestingly, the location of PCSK9 on the short arm of chromosome 1p32, adjacent to the 1p34.1p32 locus discovered and determined in sizeable French families, hinted at a possible third gene for familial hypercholesterolemia (FH), other than the already known *LDLR* and apolipoprotein B (*APOB*) genes.^11^ The specific 1p34.1p32 locus was related to the augmented hepatic function to secret cholesterol, connected to very low-density lipoprotein (VLDL), which transforms into LDL cholesterol (LDL-C) upon secretion.^35,36^ With this knowledge and the confirmed high PCSK9 expression in the liver, subsequent extensive genetic analysis involving 23 French families (displaying no *LDLR* or *APOB* variations) led to the identification of two PCSK9 variants, S127R and F216L. These revelations shed light on the genetics of hypercholesterolemia and established human *PCSK9* as an essential FH gene for LDL-C regulation.^12^ Further research into PCSK9 biosynthesis revealed that while PCSK9 also underwent autocatalytic cleavage of its prodomain in the ER, it was the only proprotein convertase (PC) that continuously remained noncovalently attached to its prodomain, even in the secreted form^15,16,19,37^ (Fig. 1a). Hence, PCSK9 acts as a protease singularly during its prodomain’s autocatalytic cleavage in the ER, suggesting LDL-C regulation by secreted PCSK9 occurs via a non-enzymatic mechanism. This clarifies the occurrence of GOF variants, uncommon for an enzyme. Subsequent research linked PCSK9 GOF variants to elevated levels of cholesterol and a heightened prevalence of coronary artery disease (CAD),^38^ whereas LOF mutations were linked with hypocholesterolemia as well as a reduced risk for the development of CAD,^23^ suggesting that normal lives can be led without functional expression of PCSK9. Moreover, heterozygote complete PCSK9 LOF variants can primarily protect individuals from cardiovascular events (CVEs) and coronary heart disease (CHD) over a lifetime.^23^

### The regulation of the biosynthesis and expression of PCSK9

Indeed, the majority of PCSK9 is synthesized by the liver, with smaller amounts also originating from the small intestine, pancreas, kidneys, lungs, and the central nervous system (CNS).^11,39^ Under typical physiological circumstances, PCSK9 is detectable in human smooth muscle cells (SMCs) but is absent in human umbilical vein endothelial cells (HUVECs), monocytes, and macrophages.^40^ However, in conditions of inflammation triggered by lipopolysaccharide (LPS), HUVECs could generate elevated levels of PCSK9.^41^ In cases of atherosclerosis, SMCs, endothelial cells, and macrophages within damaged blood vessels can generate substantial quantities of PCSK9 at not only transcriptional but also translational levels regarding various stimuli such as LPS, low shear stress, oxidized LDL (oxLDL), interleukin-1β (IL-1β), tumor necrosis factor-alpha (TNF-α), reactive oxygen species (ROS), mitochondria-derived ROS (mtROS), and mitochondrial DNA (mtDNA) released from a large amount of ruptured cells.^40,42–45^ For instance, during a myocardial infarction (MI), the ischemic cardiac tissues could significantly elevate the expression of PCSK9, especially in the border zone, potentially as a result of hypoxia and the aforementioned pro-inflammatory cytokines.^46–48^

On the transcriptional level, PCSK9 expression can be primarily controlled by sterol regulatory element-binding protein 2 (SREBP2), forkhead box O3 (FOXO3), hepatocyte nuclear factor-1α (HNF1α), and Sirtuin 6 (SIRT6).^49–52^ A sterol regulatory element (SRE) site was disclosed in the proximal region of the PCSK9 promoter by a sequence analysis of the *PCSK9* gene.^53,54^ In addition, not only the SRE site but also the neighboring upstream nucleotides play critical roles in the sterol-dependent transcriptional modulation of PCSK9, in which SREBPs can target the SRE site to function.^49^ Statin therapy that lowers cholesterol levels in the ER can also stimulate PCSK9 production by activating the upstream SREBP2, causing a poor response to statins in some patients with atherosclerotic cardiovascular disease (ASCVD),^53^ whereas insulin-induced PCSK9 transcription is dependent on SREBP1c.^54^ Conversely, caffeine can raise the Ca^2+^ level in the ER of the liver to inhibit the expression of SREBP2 at the transcriptional level, thus decreasing the levels of PCSK9 as well as CVEs.^55^

In addition, both HNF1α and HNF1β can positively regulate PCSK9 transcription, though the role of HNF1β is less well-documented.^56^ HNF1α can control PCSK9 transcription through the HNF1 site upstream from the SRE.^57^ The PCSK9 promoter could be significantly inhibited by a genetic mutation in the HNF1 site, owing to its direct and indirect impact on restricting the function of the SRE site.^50^ In the mouse models, HNF1α could be silenced by the activation of mechanistic target of rapamycin complex 1 (mTORC1) pathway, thereby suppressing PCSK9 transcription.^58^ Conversely, FOXO3 and SIRT6 are two negative regulators of PCSK9 transcription.^51^ As a nicotinamide adenine dinucleotide (NAD)^+^-dependent histone deacetylase, SIRT6 could bind to PCSK9 promoter to induce the deacetylation of histone H3, following FOXO3’s interaction with the insulin-response element (IRE) to inhibit the physiological function of the PCSK9 promoter. Both SIRT6 and FOXO3 could also hinder the activities of HNF1 and SRE at the transcriptional level to affect PCSK9 transcription.^51,59–61^ Exploring these molecular mechanisms that regulate PCSK9 biosynthesis can provide valuable insights into how to effectively reduce PCSK9 overexpression, potentially reducing the risk of ASCVDs.

### PCSK9 to regulate the degradation of LDLR and other surface receptors

Insights into PCSK9’s operational mechanism were derived from studies by Maxwell and Breslow between 2004 and 2005. These uncovered that PCSK9 overproduction significantly diminished LDLR protein levels, without altering its mRNA expression, by promoting LDLR degradation in the acidic endosomal/lysosomal pathway.^16,62–64^ Moreover, it was discovered that dietary cholesterol led to a considerable downregulation of PCSK9, while SREBP1a and SREBP2 significantly upregulated it, signifying that *PCSK9* was a gene regulated by cholesterol. This important finding was later reaffirmed by Horton and colleagues and the recognition of statins’ capacity to intensify PCSK9 transcription.^53,65^ Interestingly, while the deficiency of cholesterol and the application of statin treatment positively regulated both PCSK9 and LDLR mRNA levels, PCSK9 itself could effectively degrade LDLR protein, which could explain the mechanism underlying certain reported human mutations leading to hypercholesterolemia. As such, PCSK9 GOF mutations resulted in amplified PCSK9-induced LDLR degradation.^12,30,66^

Furthermore, two pivotal studies by Cohen et al. provided substantial support for PCSK9’s function, demonstrating a clear association between two prevalent heterozygote PCSK9 LOF variations Y142X and C679X identified in African Americans and significantly reduced LDL-C levels. These LOF variants were related to approximately 40% reductions in LDL-C, and astonishingly, an 88% lower incidence of CHD over a long follow-up period of 15 years.^23,67^ This provided the initial robust proof indicating that PCSK9 might function in a stoichiometric manner with the LDLR, diverging from typical protease behavior, as most enzymes necessitate over a 90% reduction in activity to considerably impact their function.^68^ The inactivation of PCSK9 in mice further validated this finding by showing that the absence of PCSK9 was linked with roughly three-fold higher hepatic LDLR levels and a substantial reduction in plasma LDL-C.^69^ The fact that PCSK9 knockout (KO) mice thrived, together with the identification of the initial subjects completely devoid of functional PCSK9, highlighted the potential of PCSK9 as a hopeful therapeutic option for reducing LDL-C concentrations in a clinical context.^70,71^

Moreover, it becomes clear that PCSK9 does not exhibit the function of a protease. Initial PCSK9’s structures illustrated that the C-terminal end within its prodomain, which underwent autocatalytic cleavage, was securely lodged in the groove to bind the substrate, ostensibly preventing the accessibility of substrates.^13,14^ These structural findings corroborated the initial observation that mature PCSK9 was secreted alongside its cleaved inhibitory prodomain as a complex with noncovalent interaction.^11^ Further evidence came when the PCSK9 prodomain was co-expressed with a catalytically inactive mature PCSK9 variant (S386A mutation), resulting in a fully functional, secreted PCSK9 capable of instigating LDLR degradation, just like its wild-type (WT) counterpart.^17^ This observation was then confirmed by Poirier et al., who used a PCSK9 mutant with the active site His226 mutated to Ala (H226A), resulting in similar apolipoproteinE receptor 2 (ApoER2) and very low-density lipoprotein receptor (VLDLR) degradation.^18^ Hence, PCSK9 can operate as a protease exclusively during its autocleavage of the precursor protein within ER.

As is well known, the LDLR serves as a pivotal receptor for PCSK9 in the regulation of lipid metabolism.^62^ Normally, plasma LDL-C binds to LDLR on hepatocyte surfaces, creating an LDLR-LDL-C complex that is then internalized. Inside hepatocytes, LDL-C detaches from LDLR in cytosolic endosomes and is subject to lysosomal degradation. The freed LDLR in the cytoplasm can then be recycled back to the cell surface for subsequent rounds of LDL-C transport and intracellular degradation.^72^ Pathologically, however, the breakdown of LDLR by PCSK9 has been found to start with the uptake of the PCSK9-LDLR-LDL-C complex into acidic cytosolic clathrin-coated endosomes^73–75^ (Fig. 1c). The catalytic domain of secreted or plasma PCSK9 creates a bond with LDLR’s epidermal growth factor-like repeat A (EGF-A) domain^76–79^ (Fig. 1b). This robust complex is subsequently guided to endosomes or lysosomes for decomposition through a mechanism that remains undetermined, which in turn stops LDLR recycling to transport LDL-C.^16,73,74,78^ PCSK9’s CHRD was found essential for triggering LDLR breakdown in vitro, although PCSK9 mutants lacking CHRD could still bind LDLR.^73,80–83^ Thus, it was further hypothesized that there could be a “protein X” binding the CHRD to guide PCSK9-LDLR complex to acidic compartments for degradation.^84^ Recently, two teams reported that cyclase associated actin cytoskeleton regulatory protein 1 (CAP1) and human leukocyte antigen (HLA)-C might be potential candidates for this “protein X”, respectively, which might play an important role in positively regulating PCSK9’s function on the LDLR. Jang et al. found that cytosolic CAP1, which can bind resistin, could bind the M1 and M3 modules of PCSK9’s CHRD, promoting the lysosomal degradation of the PCSK9-LDLR complex.^85,86^ Another group argued that HLA-C or a similar major histocompatibility complex class I (MHC-I) family member could guide the LDLR-PCSK9-CAP1 complex to degradation^86^ (Fig. 1b). In addition, PCSK9 might guide LDLR to decay directly from the late Golgi bodies, though this intracellular degradation route did not entirely align with the extracellular pathway.^87–90^ For reasons not yet entirely understood, the pathway involving extracellular or blood PCSK9 can be the predominant functional fashion in cells of the liver, pancreas, and small intestines.^78,91–95^ Accordingly, serum LDL-C levels show a direct correlation with circulating PCSK9 levels,^96–100^ and statins in part regulate LDL-C levels by increasing circulating PCSK9 levels in both humans and mice.^69,100,101^ Therefore, as a key regulator of cholesterol, targeting PCSK9 can be a promising therapeutic strategy for hypercholesterolemia to prevent CVD.^102^

Besides LDLR, various other receptors, ion channels, and enzymes can be regulated by PCSK9. For example, PCSK9 can regulate the breakdown of several other LDLR family members including LDLR related protein 1 (LRP1), LRP5, LRP6, ApoER2, and VLDLR, which participate in lipoprotein metabolism, triglyceride (TG) metabolism, and many other important biological processes.^18,103–106^ VLDLR and ApoER2 share a common EGF-A domain with LDLR, enabling PCSK9 to interact with both receptors and trigger their degradation, similar to LDLR^107^ (Fig. 1b). Though evidence is limited, LRPs’ interaction with PCSK9 might also depend on the EGF-like domain, a common feature of these proteins from the LDLR superfamily.^108^ Additionally, PCSK9 also plays a vital role in regulating targets involved in cholesterol metabolism outside the LDLR family, such as the cluster of differentiation 36 (CD36), ATP-binding cassette transporter A1 (ABCA1), and Niemann-Pick C1-like protein 1 (NPC1L1), which participate in fatty acid (FA) transportation, TG storage, and cholesterol efflux and absorption.^109–112^ Further, PCSK9 can also degrade CD81 which is an important entry receptor in the infection of hepatitis C virus (HCV), epithelial Na^+^ channel (ENaC) that modulates epithelial sodium reabsorption to regulate blood pressure, as well as β-site amyloid precursor protein (APP)-cleaving enzyme 1 (BACE1) that is a catalytic enzyme to generate amyloid β-peptide (Aβ) in Alzheimer’s disease (AD).^113–115^ Moreover, a recent study even revealed that PCSK9 could promote the cytosolic degradation of severe acute respiratory syndrome coronavirus 2 (SARS-CoV-2)’s key receptor, angiotensin-converting enzyme 2 (ACE2), via its binding to the pro/catalytic domains of mature PCSK9.^116^

**Fig. 2:**
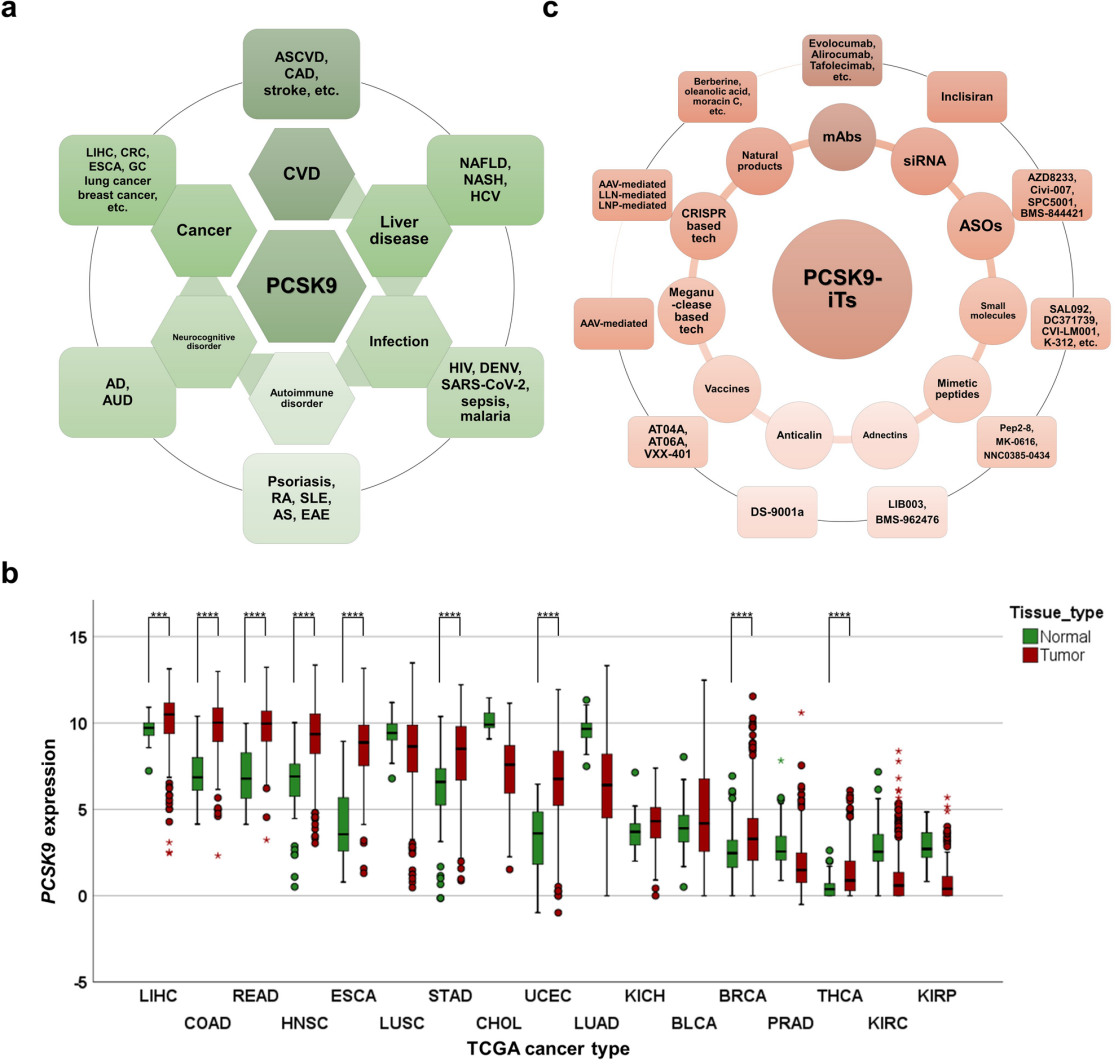
The role of PCSK9 in various disorders, its aberrant expression in cancers, and the current PCSK9-iTs. **a** PCSK9 plays an important role in various disorders including cardiovascular diseases (CVDs), liver diseases, infection, autoimmune disorders, neurocognitive disorders, and cancer. CRC colorectal cancer, GC gastric cancer. **b** PCSK9 mRNA expression across different types of cancer in TCGA datasets. LIHC liver hepatocellular carcinoma, COAD colon adenocarcinoma, READ rectum adenocarcinoma, HNSC head and neck squamous cell carcinoma, ESCA esophageal carcinoma, LUSC lung squamous cell carcinoma, STAD stomach adenocarcinoma, CHOL cholangiocarcinoma, UCEC uterine corpus endometrial carcinoma, LUAD lung adenocarcinoma, KICH kidney chromophobe, BLCA bladder urothelial carcinoma, BRCA breast invasive carcinoma, PRAD prostate adenocarcinoma, THCA thyroid carcinoma, KIRC kidney renal clear cell carcinoma, and KIRP kidney renal papillary cell carcinoma ***P < 0.001, ****P < 0.0001. **c** Current PCSK9-iTs include monoclonal antibodies (mAbs), small interfering RNA (siRNA), antisense oligonucleotide (ASO), small-molecule inhibitors, mimetic peptides, adnectin, anticalin, vaccines, meganuclease based gene editing technology, clustered regularly interspaced short palindromic repeats (CRISPR) based gene editing technology, and natural products. Panels were illustrated by IBM SPSS Statistics and Microsoft PowerPoint

Given PCSK9’s multifaceted functions in physiological and pathological activities, we will first explore its established and significant roles in CVD, liver diseases, infectious diseases, autoimmune and neurocognitive disorders, with detailed focus on its emerging mechanisms in malignancies thereafter (Fig. 2a).

## The role of PCSK9 in various disorders

### PCSK9 in CVD

#### The pivotal function of PCSK9 in triggering CVDs

As one of the key receptors regulated by PCSK9, the LDLR has been extensively investigated in numerous studies. Briefly, LDLR facilitates the absorption of LDL-C from blood into cells, crucial in humans as LDL is the primary cholesterol transporter.^117^ The liver is crucial in cholesterol metabolism and prominently expresses both PCSK9 and LDLR. The balance between LDL release and hepatocyte uptake determines circulating cholesterol levels. PCSK9 promotes LDLR degradation, resulting in elevated blood cholesterol, which heightens the risk of several CVDs such as strokes, ASCVD, and CAD. Genetic modifications in PCSK9 enhancing its LDLR-degrading function are linked to FH.^118^ In an epidemiological setting, an array of PCSK9’s single-nucleotide polymorphisms (SNPs) associated with average blood cholesterol levels that deviate from the age-adjusted normal references. They are classified as GOF or LOF, depending on their linkage to increased or decreased average blood cholesterol, respectively.^119^ Several SNPs have been experimentally proven to modify LDLR-degrading activity.^16,70^ GOF SNPs correlate with CVD risk, while LOF SNPs provide cardiovascular protection.^23,120^ Nonetheless, a clear connection may not yet be established between blood PCSK9 levels and the severity of subclinical atherosclerosis in patients who show no signs of CVD.^121^

In both human and animal studies, it has been revealed that the inherent lack of PCSK9 circulating in the bloodstream does not cause noticeable pathological conditions.^21,69–71,122,123^ Two unrelated Canadian patients suffering from FH and who were resistant to intensive statin intervention, exhibited the completely duplicated *PCSK9* gene.^124^ Individuals with elevated PCSK9 experienced substantial increases in LDL-C levels and early onset of CVEs, with one having PCSK9 levels about 20 times the standard. In contrast, those with non-functional PCSK9, specifically ΔR97/Y142X, C679X/C679X, and the monoallelic double variation R110C + V114A, were seemingly healthy and exhibited LDL-C levels nearly eight times lower than the standard.^23,70,71,125^ Genetic research in humans suggests that individuals without functional PCSK9 can also lead normal lives. Those with heterozygous complete PCSK9 LOF variations have an 88% lower risk of cardiovascular problems and CHD throughout their lifetime.^23^ Recent independent clinical studies have both shown that PCSK9 inhibitory therapies (PCSK9-iTs) could safely and effectively reduce LDL after heart transplantation, reducing patients’ risk of CVEs.^126,127^ Additionally, mice devoid of PCSK9 showed a 40–50% reduction in circulating cholesterol, approximately 80% less LDL-C, and about three to four times higher total liver LDLR levels.^69,122^ Studies using full-body and hepatocyte-specific PCSK9KO mice demonstrated that PCSK9 was only produced in hepatocytes in the liver, which was also the sole source of bloodstream PCSK9. The similar cholesterol profiles were displayed in LDLRKO or LDLR/PCSK9 double KO (DKO) mice, implying that PCSK9 primarily regulated plasma cholesterol homeostasis through the LDLR.^87,94,122^

Interestingly, PCSK9KO livers undergoing regeneration demonstrated necrotic lesions, which could be rescued by a high-cholesterol diet (HCD). This suggested that the deficiency of PCSK9 drastically diminished the levels of tissue cholesterol and provided resistance to hepatic steatosis.^122^ The initial evidence of PCSK9 contributing to the growth of atherosclerosis was observed in experimental mice fed HCD for 15 weeks. These mice, carrying the PCSK9 GOF mutation D374Y at normal levels, had significant atherosclerotic plaque formation in comparison to controls.^128^ This finding was subsequently corroborated by a single administration of recombinant adeno-associated viral (AAV) vectors encoding PCSK9 D374Y, which swiftly triggered atherosclerosis and abrogated the requirement to establish mouse models with germ-line genetic alterations.^129^ Feeding a high-fat diet (HFD) to experimental mice that have been modified with an AAV to overexpress the *PCSK9* gene resulted in not only hypercholesterolemia but also atherosclerosis.^130^ In a similar vein, transgenic pigs carrying the above-mentioned GOF D374Y mutation in the *PCSK9* gene showed a higher susceptibility to atherosclerosis compared to their WT counterparts when fed HFD and HCD.^131^ These results suggest that elevated levels or enhanced functionality of PCSK9 can advance atherosclerosis and potentially intensify inflammation. Conversely, studies involving PCSK9-deficient mice used models to assess accelerated atherosclerosis, encompassing standard, PCSK9KO, ApoEKO, and LDLRKO mice. The findings revealed a direct link between PCSK9 and atherosclerosis, with PCSK9 overexpression significantly inducing atherosclerosis and its deficiency providing cardiovascular protection.^128,132^

Mechanistic studies have unveiled that LincRNA-p21 binding to miR-221 enhanced the process of deacetylate PCSK9 by adversely impacting the expression of the silent information regulator sirtuin 1 (SIRT1). This process further bolstered proliferation, angiogenesis, and migration of arterial endothelial cells, to attenuate the progression of atherosclerosis.^133^ In cases of *ApoE*^−/−^ mice on an HFD treated with berberine, atherosclerotic plaques formation was counteracted through the inhibition of PCSK9 expression while the promotion of LDLR expression, mediated via the activation of the extracellular-signal-regulated kinase (ERK)1/2 pathway in hepatocytes.^134^ Similarly, for *ApoE*^−/−^ mice sustained on an HCD, the SIRT1 activator demonstrated an effect against atherosclerosis by lowering blood PCSK9 levels while augmenting LDLR levels.^135,136^ Moreover, PCSK9 has also been implicated in the processes of platelet activation and thrombosis.^137^ It has been evidenced that PCSK9 fosters platelet clustering, activation, and expansion as well as thrombosis by the interaction with CD36 on the surface of platelets and triggering a subsequent p38 mitogen-activated protein kinase (MAPK)/cytosolic phospholipase A2 (cPLA2)/cyclooxygenase 1 (COX-1)/thromboxane A2 (TXA2) signaling cascade.^137,138^ Mouse models indicated that an injection of PCSK9 accelerated the mesenteric artery thrombosis induced by ferric chloride through its interaction with CD36 in platelets. In the event of MI, PCSK9 induced ROS generation and induced the activation of CD36 in platelets, leading to the obstruction of microvessels and an increased size of heart infarct.^138^ Thus, employment of PCSK9-iTs may be able to mitigate cardiovascular risk by hampering platelet aggregation and coagulation through various potential mechanisms. For example, PCSK9-iTs could reduce cholesterol levels in the platelet cell membrane, thereby decreasing platelet activity.^139^ They can also potentially lower lectin-like oxLDL receptor 1 (LOX1) and oxLDL concentrations,^41,140^ which can also contribute to reducing platelet activity. In addition, they can lessen the levels of lipoprotein (a) (Lp[a]) (an independent risk factor for CVD) in the plasma, which subsequently diminishes platelet activity via peroxide-modified phospholipids.^141^ Lastly, they may be able to enhance the elimination of blood clotting factor VIII (FVIII), a crucial factor involved in the process of coagulation, by enhancing the expression of LRP1.^142^ These mechanisms of PCSK9-iTs influencing platelet activity and coagulation have been indisputably affirmed in a 2017 clinical trial utilizing Alirocumab and Evolocumab and were showed to be correlated to a reduced risk of CADs.^139^

#### The important role of PCSK9 in inflammation during CVD

Inflammation is recognized as a critical factor in the pathophysiology of CVD.^143,144^ As aforementioned, Denis and the team revealed that PCSK9-deficient mice had significantly lower aortic cholesteryl esters and less severe aortic lesions than those with normal or high levels of PCSK9. However, LDLR-deficient mice demonstrated similar levels of the accumulation of plasma cholesterol and cholesteryl ester, irrespective of PCSK9 levels, indicating that PCSK9’s influence on atherosclerosis is primarily through the LDLR.^132^ A limitation of this study is the absence of evaluations of inflammatory markers, which could elucidate the connection between PCSK9, cholesterol, and inflammation. This is notably significant since PCSK9 has been recognized as a marker of disease severity in patients with multiple traumatic injuries and is positively correlated with circulating levels of c-reactive protein (CRP).^145–151^ CRP is an acute inflammation indicator, and it has been found to promote the uptake of LDL-C into residential macrophages in the artery. It also serves as a more reliable predictor of CVD than the levels of blood LDL-C alone.^152,153^

Evidence from both experimental and clinical studies suggested that systemic inflammation could instigate the elevation of PCSK9 expression.^154^ Several theories highlight PCSK9’s role in perpetuating inflammation within atherosclerotic plaques, contributing to their enlargement and instability. Multiple studies show a correlation between PCSK9 levels and the activation of various proinflammatory genes that accelerate plaque development. Notably, in instances of MI, PCSK9 expression markedly rose in the border area of the infarction in both animal and human studies. This increase was paired with elevated expression of inflammatory factors, which could be substantially mitigated by inhibiting PCSK9.^155,156^ Krychtiuk and colleagues showed that increased circulating PCSK9 levels promoted the polarization of monocytes to a classical phenotype that has strong pro-inflammatory functions in patients with stable CAD.^157^ During chronic myocardial ischemia, raised levels of PCSK9 could also result in mtDNA damage, which activated the NLR family pyrin domain containing 3 (NLRP3) inflammasome signaling, secreted IL-1β and IL-18 to stimulate inflammation, and further promoted the pyroptosis dependent on caspase-1.^158^

In addition, PCSK9 has been demonstrated to elicit proinflammatory effects on monocytes and macrophages. For instance, the macrophages derived from THP-1 cells or primary cultured human cells incubated with human recombinant PCSK9, showed elevated mRNA expression of IL-1β, IL-6, TNF-α, C-X-C motif chemokine ligand 2 (CXCL2), and monocyte chemoattractant protein 1 (MCP-1).^159–162^ Further, Tang and colleagues also revealed that PCSK9-specific small interfering RNA (siRNA) could suppress the upregulation of proinflammatory cytokine expression in THP-1-derived macrophages triggered by oxLDL. This group also demonstrated that PCSK9 siRNA could guard against inflammation by inhibiting the activation of NF-κB in oxLDL-stimulated THP-1-derived macrophages.^161^ In 2017, Tang et al. explored the in vivo impact of PCSK9 on the expression of toll-like receptor 4 (TLR4) and nuclear factor kappa B (NF-кB) in atherosclerotic aortas, utilizing PCSK9 silencing. They observed a significant reduction of both factors in the aortas of the PCSK9 shRNA group compared to controls. This suggested that PCSK9 might modulate the release of inflammation-associated cytokines by activating the TLR4/NF-кB pathway in RAW264.7 macrophages, and the AT04A anti-PCSK9 vaccine could lower levels of NLRP3 and other inflammatory markers in these macrophages.^160,163,164^ In a recent study, PCSK9KO mice, compared to their WT counterparts, displayed reduced infarction sizes and improved heart functions. This was attributed to the suppression of M1-polarized macrophages. The inhibition of the TLR4/myeloid differentiation primary response 88 (MyD88)/NF-κB pathway was deemed crucial in this process, corroborating prior findings.^46^ Furthermore, Badimon et al. highlighted the crucial roles of PCSK9 and LRP5 in lipid uptake in human monocytes and macrophages. The silencing of LRP5 led to decreased intracellular cholesterol accumulation in macrophages, underlining LRP5’s role in lipid uptake. Significant cholesterol ester accumulation necessitated the presence of both proteins, evidenced by a notable reduction in their absence. These proteins created a complex in lipid-laden macrophages, influencing TLR4/NF-κB signaling. The absence of PCSK9 resulted in the downregulation of this pathway, underscoring its significant role in modulating inflammation.^105^ All these findings underscore that the TLR4/NF-кB signaling pathway could be a key mechanism linking PCSK9 to the inflammatory process during the development of atherosclerosis.

Moreover, Giunzioni and colleagues reported that the inflammation induced by PCSK9 in the formation of atherosclerosis was linked to the recruitment of massive amounts of inflammatory monocytes and their subsequent transformation into macrophages, which was relied on the existence of LDLR. LPS could promote this process, resulting in elevated levels of IL-1β and TNF-α while a reduction in anti-inflammatory factors such as arginase 1 (ARG1) and IL-10.^45^ The similar results were also observed by Barcena et al. that PCSK9 could also suppress the anti-inflammatory action mediated by VLDL in human macrophages by inhibiting VLDLR, whilst PCSK9-iTs could reverse the pro-inflammatory function in the experimental studies.^165,166^ LRP5 and LRP6, as two potential PCSK9’s coreceptors, could also exacerbate atherosclerosis through the activation of the Wnt/β-catenin pathway, leading to the promotion of vascular SMCs (vSMCs) while suppressing anti-inflammatory macrophages.^105,106^ PCSK9-iTs could also act by decreasing the migration of monocytes into the plaques of atherosclerosis, which was linked to increased levels of the anti-inflammatory cytokine IL-10, resulting in a reduction in the expression of TNF-α and C-C chemokine receptor 2 (CCR2), both of which controlled the entrance of monocytes into those plaques.^167–169^ In addition, elevated PCSK9 levels have been shown to foster the maturation of dendritic cells (DCs) and drive the evolution of naive CD4^+^ T cells into the Th1 and Th17 lymphocytes, leading to an uptick in the secretion of cytokines IFNγ and IL-17A. Conversely, PCSK9-iTs could lead naive CD4^+^ T cells to differentiate into regulatory T cells (Tregs) and stimulate the synthesis and discharge of IL-10 and transforming growth factor beta (TGF-β), thus countering inflammation. This contributed to a reduction in inflammatory activities and an optimistic outcome for ASCVDs.^170^

Despite these studies’ limitations, they do not discount the possibility of LDLR family members being the major receptors that mediate PCSK9’s inflammatory stimulation. However, the involvement of PCSK9 in inflammatory activities can still remain debatable due to inconclusive results from clinical studies regarding PCSK9-iTs’ effects on inflammation markers and the inconsistent relationship between PCSK9 concentrations and the evolution of atherosclerosis in the general population.^43,72,171–175^ More research is warranted to fully examine PCSK9’s role in bridging cholesterol and inflammation and the scope of its cholesterol-independent regulatory function during inflammation.^176–178^

### PCSK9 in liver diseases

#### PCSK9 and non-alcoholic fatty liver disease (NAFLD)/non-alcoholic steatohepatitis (NASH)

As is well known, liver is the predominant organ to produce and clear PCSK9 in the body.^122,179^ NAFLD is a globally common liver condition, impacting approximately 25–30% of the global population, which is a disorder characterized by the unusual build-up of TG in hepatocytes. It is identified as hepatic steatosis when over 5% of hepatocytes exhibit TG droplets. Further, NAFLD has the potential to advance into NASH, typified by inflammation, and eventually to cirrhosis, which is a substantial risk factor for hepatocellular carcinoma (HCC).^180^ There are suggestions of an association between PCSK9’s role in TG metabolism at the intestinal level and the NAFLD pathogenesis due to its plasma concentration.^181,182^ Recent discoveries have underscored the significance of PCSK9 in managing liver lipid balance.^183^

Various preclinical and clinical studies have already identified a relationship between PCSK9 and NAFLD, suggesting that bloodstream PCSK9 is able to limit lipid uptake and their subsequent accumulation in the liver. Evidence showed that HFD could induce liver steatosis and raise both circulating and hepatic PCSK9 levels in mice.^184^ Research by Demers and colleagues revealed PCSK9’s ability to regulate CD36 expression, a key influencer of FA uptake and a contributing factor to liver steatosis. Further studies also suggested that PCSK9 could control FA uptake in immortalized hepatocytes, dependent on CD36. Additionally, *Pcsk9*^−/−^ mice showed an increase in liver lipid accumulation and CD36 expression, and when subjected to HFD, these mice developed severe liver steatosis and fibrosis.^109,185^ This ground-breaking research suggested that PCSK9 could degrade CD36 through interaction with its extracellular loop and mediation of its internalization. PCSK9 inhibition models observed an increase in hepatic TG both in cellular and animal models, indicating that a rise in hepatic levels of CD36 could increase NAFLD susceptibility.^109,185^ Recently, Ioannou and colleagues also confirmed this finding in their experiments, showing that PCSK9 deletion exacerbated murine NASH. After nine months of HFD/HCD, PCSK9-deficient mice displayed elevated crystallization levels of hepatic cholesterol, increased crown-like structures developed in macrophages, heightened levels of apoptosis and inflammation, and a rocketing 11-fold elevation in liver fibrosis versus control groups.^186^ Interestingly, current anti-PCSK9 monoclonal antibodies (mAbs), whether used alone, combined with diet, or other lipid-lowering agents, cause an increase in blood PCSK9 levels by inhibiting its interaction and degradation with the LDLR.^187^ Furthermore, the overexpressed PCSK9 Q152H LOF variant that can be retained in the ER by adenovirus in mice surprisingly protected against liver damage,^21^ aligning with findings from an animal study of alcoholic liver disease where Alirocumab significantly reduced PCSK9 levels, consequently reducing infiltrating fat, inflammatory activities, oxidative stress, and liver injuries.^188^ Conversely, there is contradictory evidence suggesting that specific overexpression of human PCSK9 in mice liver could lead to NAFLD as well as fibrosis when subjected to a diet challenge.^189^ E2F1 was also demonstrated as a critical regulator of PCSK9, as *E2f1*^−/−^ mice on HCD exhibited increased liver lipid accumulation and fibrosis that could be reversed by re-expressing hepatic PCSK9.^190^

The role of PCSK9 in promoting steatosis, a condition characterized by the buildup of fat in the liver, is widely supported by preclinical evidence. However, clinical findings remain disputed. Research by Lai and colleagues revealed that hepatic PCSK9 expression levels increased with the seriousness of steatosis.^190^ Among severely obese patients, an inverted relationship was observed between hepatic PCSK9 expression levels and the extent of fat accumulation, whereas circulating PCSK9 levels displayed a positive correlation with the severity of liver steatosis.^191^ This finding was further supported by a larger study involving 698 participants that revealed a strong connection between circulating PCSK9 levels and all plasma indicators to determine hepatic function, as well as the existence of the steatosis in the liver.^180^ Contrary to these observations, a clinical study involving 201 patients reported that in those morbidly obese patients with steatosis undergoing bariatric surgery, there was a positive correlation between hepatic PCSK9 expression and blood PCSK9 levels.^192^ Yet, in another clinical study of 478 patients with type 2 diabetes (T2D) or metabolic syndrome by Wargny and colleagues, no connection was found between blood PCSK9 and hepatic enzymes in obese patients, nor was there a relationship between hepatic fat and blood PCSK9 concentrations or hepatic PCSK9 mRNA levels.^193^ To identify PCSK9’s specific effects on liver health, there have been three independent clinical investigations to examine the impact of PCSK9 LOF variations on hepatic steatosis as well as liver functions.^21,189,194^ Among these studies, subjects carrying the PCSK9 Q152H LOF variation showed normal hepatic activities and functions in spite of PCSK9’s lifelong retention,^21^ potentially indicating that the Q152H variant mitigates hepatic impairment. However, PCSK9 R46L LOF variant was initially investigated to show that its carriers could have a twice elevation in the incidence rate of steatosis in the liver,^194^ whilst the latest findings suggested that PCSK9’s R46L variation could protect NAFLD patients from hepatic impairment.^189^ Although the association between PCSK9 LOF variations and NAFLD still remains unclear, PCSK9-iTs may represent a potential alternative therapy for patients diagnosed with NAFLD/NASH in the clinic. Scicali et al. found that PCSK9-iTs significantly improved many biomarkers for steatosis in FH patients and achieved an elevated high-density lipoprotein (HDL)/TG ratio.^195^ Besides, the favorable impact of PCSK9-iTs on hepatic activities and functions in NAFLD patients was also observed in a retrospective clinical analysis.^196^ Further, Sekhon et al. reported that Evolocumab led to an over 80% reduction in liver transaminases in a NASH patient, and the subsequent liver biopsy revealed normalized histology.^197^

#### PCSK9 and HCV

The LDLR has been suggested as a factor involved in HCV’s entry into hepatocytes, although this role is still debated.^198,199^ A recent study utilizing hepatocytes from induced pluripotent stem cells (iPSCs) of a patient with non-functional LDLR found that these cells were vulnerable to HCV infection, with a surge in viral production upon reintroduction of functional LDLR. This indicates that LDLR may have a more crucial role in HCV packaging and its interaction with cellular lipid metabolism than in facilitating viral entry.^200^ Interferon (IFN)-free direct-acting antivirals (DAAs) have dramatically improved the sustained virological response rate in HCV therapy.^201^ In response to the therapy based on IFN and supplemented with DAAs, patients who responded well exhibited a significant rise in plasma PCSK9 levels. This implied PCSK9’s protective role in preventing HCV’s infectious activities in Huh-7.5.1 HCC cells.^202^ Moreover, patients suffering from chronic infection of HCV genotype 3 (HCV-G3) displayed lower concentrations of blood PCSK9 and LDL-C, likely due to enhanced LDLR activities. This contrasts with the observations from HCV-G1-infected patients, as HCV-G1 relied more on the scavenger receptor class B type I (SR-B1) for viral entry.^203^ Nevertheless, it is still needed to be examined whether blood PCSK9 levels can be used as a reliable biomarker for HCV infection. Instead, a more accurate evaluation of circulating PCSK9 activity could be achieved by assessing the ratios of not only phosphorylated versus non-phosphorylated PCSK9 but also active versus inactive PCSK9 using mass spectrometry.^26^ Consequently, the clinical application of PCSK9-iTs in the patient population with HCV infection should be approached with caution, as it could increase hepatic LDLR levels and possibly promote HCV’s packaging and infectious activities in the host.^200,204,205^ In addition, PCSK9 has been observed to degrade some of the crucial surface receptors that could mediate the entry of HCV into hepatocytes, such as VLDLR and CD81. This finding implies that PCSK9 could potentially hinder HCV infection in certain instances.^113,206^

In summary, liver diseases can have diverse triggers, and PCSK9 expression may be associated with specific types of liver diseases, for example, NAFLD/NASH and HCV. PCSK9 expression may also correlate with liver diseases at specific stages and may function in different ways. Although some studies imply that PCSK9-iTs could be beneficial in treating hepatic disorders such as steatosis, caution should be exercised when prescribing PCSK9-iTs for patients with HCV infection. Therefore, more extensive, well-organized, and large-scale clinical trials are required to validate these findings among different liver diseases.

### PCSK9 in infectious diseases

#### PCSK9 and viral infection

It is noted that the important roles of PCs, particularly Furin and SKI-1/S1P, in enhancing the ability of enveloped viruses to invade host cells and increase their infectivity has been well-established.^207^ PCSK9, as the latest member of PC family, has also been associated with the infectivity of several viruses, including HCV, human immunodeficiency virus (HIV), Dengue virus (DENV), as well as potentially SARS-CoV-2.^208^ Since the association between PCSK9 and HCV has already been introduced in the previous section, we will mainly focus on the association of PCSK9 and the other three viral infections in the following section. Clinically, dyslipidemia is prevalent in HIV patients, elevating their risk for CVD, potentially due to both HIV infection and certain antiretroviral therapies (ART). Managing lipid alterations in such patients is challenging. Circulating PCSK9 levels are noted to be around 65% higher in HIV patients on ART, correlating with endothelial dysfunction.^209^ HIV-related atherosclerosis is marked by heightened vascular inflammation, impaired endothelial cell function, and a prevalence of non-calcified plaques. This condition may be rapidly reversible with Evolocumab administration.^210^

Furthermore, as a positive-stranded RNA virus, DENV is responsible for over 400 million infections and approximately 25,000 deaths annually.^211^ A study highlighted elevated PCSK9 levels in the blood of patients infected with DENV, correlating to heightened levels of DENV viremia and significantly severe plasma leakage.^212^ Subsequent findings demonstrated that DENV infection escalated PCSK9 expression in hepatocytes, leading to decreased surface LDLR levels and hindered intracellular LDL-C uptake. This facilitated the de novo synthesis of cholesterol via the SREBP-2 signaling pathway,^213^ potentially exploited by DENV for viral packaging.^212^ Elevated ER cholesterol post-DENV infection was found to significantly suppress the antiviral type I IFN response, due to cholesterol-induced reduction and impairment of activated stimulator of IFN genes (STING).^212^ Testing with Alirocumab in the DENV context revealed enhanced LDLR levels and reduced viremia, indicating that PCSK9-iTs could offer therapeutic advantages in DENV patients by revitalizing antiviral IFN responses.^212^ Moreover, Li et al. proposed that proPCSK9 might diminish cellular IFNβ levels by inhibiting activating transcription factor 2 (ATF2) functions in the ER. However, the interaction between ER-localized proPCSK9 and cytosolic ATF2 remains unclear. If validated, this suggested dual mechanism—proPCSK9 inhibiting IFNβ via ATF2 suppression and mature PCSK9 enhancing ER cholesterol leading to STING inhibition—would emphasize PCSK9’s substantial role as an IFN expression suppressor. This would solidify PCSK9’s potential as a target for managing viral infections.^214^ Hence, further delicate clinical studies are needed to test this hypothesis in the future.

Recently, in the midst of the COVID-19 pandemic, some animal studies reported that statins could elevate the levels of SARS-CoV-2 receptor, ACE2.^215,216^ However, statins have also been observed to substantially improve the outcomes of COVID-19 patients over 65 years old.^217–219^ This may be attributed to statins’ ability to enhance endothelial cell functions and reduce vascular coagulation and inflammation through both cholesterol-dependent and independent mechanisms.^220^ Given that PCSK9 mAbs can further significantly decrease LDL-C by about 60% and substantially reduce the incidence of CVEs in patients treated by statin therapy, it has been suggested to also administer PCSK9-iTs to selected COVID-19 patients who could benefit from increased IFN levels.^221^ This is particularly related to patients who carry LOF variants in the TLR3- and IFN regulatory factor 7 (IRF7)-dependent IFN immunity.^222^ In the recent IMPACT-SIRIO 5 pilot clinical trial (NCT04941105), 60 severe COVID-19 patients were randomized to receive either a single 140 mg dose of Evolocumab or a placebo. The findings indicated that within 30 days, the Evolocumab group experienced lower mortality or intubation rates (23.3%) compared to the placebo group (53.3%), and also exhibited a notable reduction in IL-6 levels (−56%) compared to the placebo group (−21%).^223^ Particularly, the patients with higher initial IL-6 levels demonstrated lower mortality when treated with Evolocumab, suggesting that the intensity of inflammation might dictate the therapeutic benefits. As a result, PCSK9-iT appeared to reduce the mortality or requirement for intubation and inflammatory activities in patients with severe SARS-CoV-2 infection.^223,224^

Therefore, conducting more large-scale and well-organized clinical studies will be essential to corroborate and advocate for the further application of PCSK9-iTs in the management of viral infections in the future.

#### PCSK9 and sepsis

As a severe and life-threatening complication of bacterial infection, sepsis can be frequently induced by diverse bacterial and pathogenic entities that instigate a runaway systemic inflammation, leading to the failure of multiple organs.^225^ Despite the prevalent use of antibiotics, no other effective treatments for septic shock exist to date.^226^ Over the past decade, a few studies in animals and human subjects have investigated the potential beneficial role of PCSK9 deficiency in sepsis. Remarkably, PCSK9KO mice showed resistance to septic shock caused by LPS exposure,^227^ whereas PCSK9 LOF variants were associated with fewer instances of septic shocks and organ failures,^228,229^ unlike the scenario in those transgenic mice with high levels of PCSK9 expression.^230^ Further, the LDLR is known to rid the system of gram-positive lipoteichoic acid as well as gram-negative LPS, which are identified pathophysiological exacerbators for sepsis, through an LDL-dependent manner.^231^

Given that LDLRKO mice did not exhibit the protective effects seen when PCSK9 was absent, it has been hypothesized that PCSK9 deficiency or the application of PCSK9-iTs could boost the clearance of pathogenic lipids via LDLR recycling.^232,233^ In sepsis experimental models, like cecal ligation and puncture, PCSK9KO mice exhibited reduced bacterial presence in circulation, lungs, and peritoneal cavity fluid compared to WT counterparts, enhancing the containment and clearance of bacterial infections without PCSK9.^230^ Validating this hypothesis, clinical studies revealed that patients with three PCSK9 LOF variants (R46L, A53V, and I474V) displayed a survival rate increase of over 50% after one year and demonstrated reduced susceptibility to recurrent infections, likely due to improved infection resolution and/or bacterial clearance. This suggested that potent, possibly combined, PCSK9 LOF variants might be beneficial, whereas single weak variants might not suffice for protection.^234,235^ PCSK9 is also implicated in routing ApoER2, VLDLR, and CD36 to their degradation in lysosomes, especially in adipose tissues that express high levels of those receptors.^18,109^ Additionally, it is revealed that LPS could be retained in adipose tissue through the VLDLR. Notably, a homozygous intronic GOF variant of VLDLR has been linked to improved survival rates from sepsis, particularly in patients with a body mass index (BMI) < 25.^236^

Despite these findings, there are challenges in extrapolating the results from animal studies to clinical practice.^237^ These issues must be addressed before PCSK9-iTs can be routinely prescribed to patients with sepsis. Moreover, sepsis intricacies differ across species, leading to skepticism about the universal applicability of rodent sepsis models.^238^ It is worth noting that rodents are significantly more resistant to sepsis than humans, although humanized mouse sepsis models have been developed to somewhat counter this limitation.^239^ Furthermore, while PCSK9-iTs may improve survival rates for adult sepsis patients, children or infants with sepsis might not benefit from PCSK9-iTs since PCSK9 LOF has been linked to poor survival in young mice and children. Until the clarification of this PCSK9 LOF paradoxical effect, it is advised that children should not participate in clinical trials to investigate PCSK9-iTs for sepsis.^240^ Indeed, it has been observed that patients with septic shock who exhibited lower levels of blood PCSK9 (within the first quartile) on the first day after onset had the highest 28- and 90-day death rates in comparison to patients in other quartiles.^241^ This suggested that lower circulating PCSK9 levels on the first day following the initiation of sepsis did not relate to a more optimistic outcome.^242,243^ Nonetheless, these findings do not rule out the potential preventive applications of PCSK9-iTs, such as their administration before surgery to neutralize free PCSK9 in the circulation and thereby elevate LDLR levels, which may help prevent the occurrence of sepsis. Furthermore, PCSK9 failed to substantially affect blood HDL levels,^12^ an important factor since septic patients with decreasing blood HDL levels displayed an increased risk of organ failure and mortality.^244^

A recent meta-analysis of 20 double-blind, randomized, placebo-controlled trials, encompassing 64,984 participants, was conducted to determine the impact of PCSK9-iTs on the occurrence of sepsis and other severe infections. The analysis revealed no significant association between PCSK9-iTs usage and the risk of sepsis, serious systemic infections, or severe organ-specific infections compared to a placebo. These results, indicating no increase or decrease in the incidence of serious infectious events with PCSK9-iTs, affirmed their safety for patients concerned about potential infection-related side effects.^245^

#### PCSK9 and parasitic infection

Parasites are entities that are dependent on cholesterol for their growth and development. However, they lack the innate capability to synthesize cholesterol, therefore, they source it from their hosts.^246^ In a study of 752 Malian children, Arama et al. reported that the children possessing PCSK9 GOF mutations were susceptible to a more severe disease trajectory of malaria,^247^ whereas Fedoryak and colleagues revealed that PCSK9 LOF mutations were linked to a decrease in malaria-related mortality.^248^ These findings give rise to the proposition that PCSK9-iTs could potentially serve as therapeutic and preventive measures for malaria. Nonetheless, as of now, there is no empirical data supporting a relationship between PCSK9-iT and malaria progression. Further extensive studies are warranted in this area.

### PCSK9 in autoimmune diseases

#### PCSK9 and psoriasis

Several previous studies have shown an elevated occurrence of related diseases like obesity, lipid disorders, T2D, arterial hypertension, and impaired liver function in patients with psoriasis.^249–251^ Evidence indicates that individuals with psoriasis may experience a reduced lifespan by approximately five years, primarily due to MI and thromboembolic events. This is largely due to psoriasis’ connection with abnormalities in lipid metabolic regulation, a crucial factor in initiating and advancing atherosclerosis.^252^ In addition, patients with psoriasis have been observed to possess higher LDL and TG levels while lower HDL levels.^253^ OxLDL, absent in the healthy skin of psoriasis patients, has been found in psoriatic epidermis. A correlation has been established between specific antibodies against these altered lipoproteins as well as the seriousness of psoriasis.^254^ The link between psoriasis and lipid metabolism disorders is multifactorial, involving genetic, environmental, and immunological elements, with inflammation playing a significant role. For example, adipocytes, under the influence of inflammatory modulators found in psoriasis, can produce CRP, highlighting the association between abundant adipose cells and chronic inflammatory skin lesions.^255^ Additionally, clinical therapeutics, such as statins, for the treatment of hypercholesterolemia, have been found to enhance the effectiveness of psoriasis treatment.^256^

Several studies exploring the link between PCSK9 and psoriasis have pinpointed the protein as a potential contributor to psoriasis susceptibility and progression. A study led by Merleev identified a potential locus at 1p32.3, associated with psoriasis susceptibility, situated within PCSK9 (rs662145 C > T). It was discovered that the homozygous PCSK9 SNP rs662145 C > T correlates with reduced PCSK9 expression but elevated IL36G expression in both in vitro keratinocytes and nonlesional human skin tissue, compared to its heterozygous counterparts.^257^ A study by Luan et al. noted elevated PCSK9 expression in psoriatic skin lesions, with PCSK9 mRNA levels approximately five times higher in psoriatic plaques than in normal skin. They also examined PCSK9 expression in mice treated with imiquimod (IMQ), which can induce psoriasis-like lesions, observing increased PCSK9 expression in IMQ-treated mice, suggesting that IMQ may provoke PCSK9 expression and the formation of psoriatic lesions. However, mice with PCSK9 knockdown (KD) did not develop psoriatic lesions after IMQ treatment.^258^ This hints at a significant role of PCSK9 in psoriatic plaque formation, which might be associated with the relationship between PCSK9, Janus kinase (JAK), and ERK signaling pathway. An increase in ERKs in psoriatic skin lesions was observed, with ERKs activity normalizing upon clearance of psoriasis.^259^

Further research has explored the relationship between serum PCSK9 levels and various aspects of psoriasis, including disease severity, inflammation, metabolic syndrome, and the effects of systemic therapies. One study found increased PCSK9 levels in psoriasis patients, irrespective of disease severity. Importantly, it revealed that methotrexate (MTX) treatment for psoriasis reduced PCSK9 levels, while acitretin treatment increased them, suggesting that different systemic therapies may have varied effects on PCSK9 levels, potentially influencing lipid metabolism and cardiovascular risk.^260^ Moreover, research conducted by Garshick et al. further demonstrated a substantial connection between bloodstream PCSK9 levels and both initial and late stages of atherosclerosis in patients with psoriasis, independent of blood cholesterol levels.^261^ Therefore, targeting PCSK9 could be an emerging treatment choice for psoriasis patients, which was suggested by Zhao and colleagues that genetically mediated inhibition of PCSK9 was linked with a reduced risk of psoriasis in their Mendelian randomization study.^262^

#### PCSK9 and rheumatoid arthritis (RA)

RA, with a prevalence of 0.5–1%, not only puts a strain on individuals but also carries significant societal costs.^263^ The prognosis has significantly improved due to the introduction of biological treatments, among which the antagonists of TNF-α were the first to be introduced, often combined with MTX and other prescribed disease-modifying medications.^264^ It is noteworthy that the use of combination therapy in RA is quite common. Despite the availability of other biologics with diverse effects to inhibit various cytokines to counter inflammation during RA, approximately 30% of RA patients remain non-responsive.^265,266^ Similar to several other autoimmune disorders, RA patients face an elevated incidence of atherosclerotic events and their complications. Arida et al. found that the plasma concentration of PCSK9, as well as the ratio of PCSK9 to LDLR, demonstrated a positive correlation with the onset and progression of atherosclerosis in many RA patients. This risk could potentially be reduced through the use of biological therapies.^267–270^ In RA patients receiving TNF-α antagonists, an inverse correlation was found between initial PCSK9 levels and disease activity extent. Those in the lowest 25% of PCSK9 measurements had a four-fold increased chance of achieving remission, marked by the absence of active symptoms. This suggests the potential role of PCSK9 as a marker to predict non-responsiveness to biological treatments in RA.^271^ Moreover, in a recent study involving 89 RA patients and 50 controls, higher blood PCSK9 levels were observed in RA patients, showing a positive correlation with Th17 cells, Th17/Treg ratio, CRP, and disease activity score (DAS), but not with Th1, Th2 cells, or Th1/Th2 ratio. Remarkably, PCSK9 levels decreased in patients treated with conventional synthetic disease-modifying anti-rheumatic drugs (csDMARDs), with larger reductions correlating with a higher likelihood of response and remission. This underscored the potential of PCSK9 as a reliable marker in RA management to predict csDMARD outcomes.^272^

In RA, the joint’s synovium is invaded by lymphocytes, neutrophils, and activated macrophages, all of which play a direct role in disease progression. Additionally, signs of activation can be seen in synovial cells, contributing to persistent inflammation.^273,274^ It has been demonstrated that PCSK9, at physiological levels, can induce macrophages to release IL-1ß and TNF-α, key contributors to the inflammatory processes in RA, a fact underscored by the effectiveness of biologics that specifically target these cytokines.^275^ PCSK9 could also induce the secretion of MCP-1 from human synoviocytes in vitro. MCP-1 is believed to be an essential player in the pathological process of RA development, including by attracting macrophages. When MCP-1 was blocked, it was observed to lessen arthritis in rat models, and elevated MCP-1 levels have been found in RA patients.^276–278^ As a result, PCSK9-triggered MCP-1 could be involved in attracting mononuclear leukocytes. PCSK9 antibodies have been shown to inhibit TNF-α and IL-1ß from macrophages, as well as MCP-1 from synoviocytes.^275^ Therefore, PCSK9-iTs may offer therapeutic benefits in RA, potentially more so in patients with high PCSK9 levels. Furthermore, many foam cells containing oxLDL were also identified in RA, indicating possible shared mechanisms with atherosclerosis. Increased oxLDL levels are associated with RA and are linked to cardiovascular diseases in RA.^279–281^ The induction of immune inhibitory Tregs is generally considered beneficial in RA.^282^ Hence, if oxLDL plays a role in RA, the ways in which PCSK9 mitigates oxLDL’s proinflammatory and immune-activating properties could be relevant to this disease.

#### PCSK9 and systemic lupus erythematosus (SLE)

In 1970s, it was demonstrated that there was an escalated risk of CVDs in patients suffering from SLE.^283^ Current estimates suggest that their risk is 2–10 times greater compared to the general population. For example, the incidence of MI in premenopausal women SLE patients can increase by 50 times compared to healthy individuals. However, the underlying mechanisms contributing to these observations remain to be fully understood.^284^ These may be partially explained by the existence of standard cardiovascular factors, such as dyslipidemia, but the unchanged cardiovascular risk in SLE patients post-statin treatment suggests otherwise.^285–287^ There is also an increased focus on the impact of inflammation to enhance the risk of atherosclerosis in SLE patients.^285^

In a study by Fang et al., serum PCSK9 levels were examined in 90 SLE patients undergoing varied pharmaceutical treatments and compared to 50 control subjects. All SLE patients were administered hydroxychloroquine, with subsets receiving additional medications. There were no significant differences in traditional cardiovascular risk factors between the SLE and control groups. However, SLE patients exhibited significantly elevated serum PCSK9 levels compared to controls, suggesting a potential role of PCSK9 in the increased incidence of CVEs in SLE patients. The exact mechanisms and pathways of PCSK9’s impact in this context are yet to be elucidated.^145^

Moreover, in a study by Liu et al., involving 109 SLE patients and 91 controls, serum PCSK9 levels, intima/media complex thickness, atherosclerosis presence in the jugular arteries, and PCSK9’s influence on the differentiation of monocytes into DCs were investigated. Unlike Fang et al., this study found no elevated serum PCSK9 levels in SLE patients; however, participants in Liu’s study were notably younger. A significant correlation was found between PCSK9 concentrations and SLE severity as measured by the systemic lupus activity measure (SLAM) and SLE disease activity index (SLEDAI). The study highlighted a mechanism by which PCSK9 may influence SLE progression, revealing elevated oxLDL levels in SLE patients and its role in stimulating the activation and maturation of DCs as PCSK9-dependent antigen-presenting cells (APCs), offering insights into PCSK9’s role in SLE progression.^288^

Furthermore, A comprehensive study by Mok et al., involving 539 SLE patients, corroborated previous findings, exploring the correlation between blood PCSK9 levels, disease activity, and major adverse cardiovascular events (MACEs). The study revealed that higher PCSK9 levels were associated with increased SLEDAI scores and a pronounced incidence of MACEs over five years, with an HR of 2.51 (95%CI 1.11–5.70). The link between elevated PCSK9 concentrations and MACEs remained significant after adjusting for various factors, showing an independent association with all-cause death rate and vascular mortality. This research emphasized the influential role of PCSK9 levels in assessing disease activity and cardiovascular risk in SLE patients.^289^ Therefore, serum PCSK9 concentrations are evidently correlated with the severity of disease activity in SLE. Elevated PCSK9 levels are associated with a heightened risk of CVEs and mortality in SLE patients, suggesting the potential of PCSK9 as a promising target for developing future therapeutic strategies for SLE.

#### Other autoimmune disorders

In a comprehensive study by Cai et al., 89 active ankylosing spondylitis (AS) patients, 20 osteoarthritis patients, and 20 healthy individuals were examined. The findings revealed significantly higher blood PCSK9 concentrations in AS patients compared to controls, correlating positively with CRP and disease activity, but not with other clinical markers. Additionally, a unique association was identified between PCSK9 and both Th17 cells and IL-17A, which was not observed with IFNγ, Th1, or Th2 cells. Notably, PCSK9 levels generally diminished from baseline to the 12^th^ week in AS patients, with a more pronounced decrease in responders than in non-responders. These results suggest a potential link between serum PCSK9 and disease activity and Th17 cells in AS, with short-term reductions possibly indicating a positive treatment response.^290^ In further research, two clinical studies proposed that PCSK9 mAbs might present a secure, enduring alternative for lowering cholesterol, avoiding necrotizing myositis in patients afflicted with statin-associated immune-mediated myopathy. This ailment is typically linked with heightened expression of 3-hydroxy-3-methylglutaryl coenzyme A reductase (HMGCR) and elevated antibodies against HMGCR levels.^291,292^ However, in a mouse model of experimental autoimmune encephalomyelitis (EAE), Alirocumab application appeared to have no effect on EAE progression or immune response, implying that blood cholesterol levels may not directly affect neuro-inflammatory diseases and that the protective benefits of statins may not be related to levels of circulating cholesterol.^293^

### PCSK9 in neurocognitive disorders

The prominence of PCSK9 in relation to the CNS is unquestionable, given its initial designation as NARC1. The brain, intriguingly, is the repository for the highest concentration of cholesterol, containing nearly a quarter of the total cholesterol in the human body. PCSK9 facilitates the modulation of LDL uptake into brain endothelial cells via its regulatory influence over the LDLR.^294^ At the same time, glial cells produce HDL for cerebral use, and the main component of its apolipoprotein is ApoE, a protein whose regulation is also under the purview of PCSK9.^295^ Although the blood-brain barrier (BBB) customarily prohibits the transportation of cholesterol and PCSK9 into the brain, the levels of PCSK9 can experience an upregulation and dynamic regulation in the CNS under specific disease states.^296–300^ PCSK9, initially identified for its role in neuronal apoptosis, is characterized by high expression levels in telencephalon neurons. Here, it fosters neuronal differentiation and manages cellular apoptosis during the process of neurogenesis.^12,27^ Indeed, there has been increasing evidence suggesting a link between lipid metabolism, especially cholesterol homeostasis, and the pathogenesis of neurodegenerative diseases, for example, AD. It is well-documented that cholesterol plays a crucial role in the brain, contributing to myelin formation, synaptogenesis, and neurotransmission.^301^ Observations have indicated that a dysfunctional lipid metabolism can precipitate neuromuscular junction denervation, impair neuronal transport, and engender mitochondrial and cytoskeletal dysfunction.^302^ Given these insights, increasing researchers have started to explore PCSK9’s potential involvement in neurocognitive disorders. As the understanding of PCSK9’s role in the CNS deepens, it could potentially offer new avenues for the treatment of these debilitating neurocognitive disorders.

#### PCSK9 and AD

First identified by Wu and colleagues, PCSK9 was found to promote neuronal cell death by increasing caspase activity and suppressing ApoER2 expression.^303^ Consistent with this finding, the silencing of PCSK9 in mice provided a defense against neuronal apoptosis induced by cerebral ischemia, thereby mitigating the advancement of brain damage.^304^ However, the exact association between PCSK9 and AD remains nebulous. A defining attribute of AD is the aggregation of Aβ plaques, resulting from modifications in the regulatory pathways of Aβ, managed by APP and BACE1.^305^ Due to BACE1’s role in Aβ clearance, it is a noteworthy element in the progression of AD. Recent explorations into AD pathology have highlighted a crucial link between dyslipidemia and AD. PCSK9 has been implicated in reducing brain cholesterol intake by breaking down LRP1, thereby decreasing the clearance of Aβ in the CNS.^305,306^ Inhibiting PCSK9 in mice led to diminished Aβ accumulation in brain areas such as the prefrontal cortex and hippocampus, but this effect was not observed in *Lrp1*^−/−^ mice.^307^ A study by Abuelezz et al. revealed an association between the use of Alirocumab and improvements in cognitive performance, cholesterol regulation, and a reduction in neuro-inflammation. Alirocumab demonstrated the ability to boost hippocampal LRP1 expression and reduce various molecules such as brain cholesterol, hippocampal BACE1, Aβ(42), high-mobility-group-box-1 protein (HMGB1), receptor for advanced-glycation-end-products (RAGE), and TLR4. This was concomitant with a consequent decline in various inflammatory modulators including IL-1β, IL-6, NF-κB, and TNF-α.^305^ Similar observations were made by Hendawy and the team in a rat-based study exploring PCSK9 inhibition and depressive-like behavior. They found that Alirocumab could alleviate alterations in hippocampal kynurenine/tryptophan levels and the pattern of pro-inflammatory cytokines such as IL-1β, IL-2, IL-6, and TNF-α, which were induced by chronic unpredictable mild stress (CUMS). Alirocumab was also found to favorably modulate NF-κB, indoleamine 2,3-dioxygenase 1 (IDO-1), HMGB1/RAGE/TLR4 axis, and NLRP3 inflammasome complex in the hippocampal brain region of CUMS-affected rats.^308^

In addition, research by Zimetti and associates reported dramatically higher PCSK9 levels in the cerebrospinal fluid (CSF) of AD patients in comparison to non-AD controls,^300^ while Courtemanche et al. argued that increased CSF PCSK9 concentrations could be a common feature of many neurodegenerative disorders, not only AD.^309^ Another recent animal study demonstrated that PCSK9-iT helped protect against the loss of dendritic spines by impeding the formation of amyloid plaque and neuroinflammation.^310^ However, some studies failed to ascertain a direct impact of PCSK9 on the BACE1 expression or Aβ levels in animal models, indicating that the influence of PCSK9 on AD could be tissue-dependent, or necessitate additional modulation by other regulatory elements, or require a longer exposure period.^311,312^

Further, examining brain samples post-mortem offered animal evidence supporting PCSK9’s role in AD progression. Picard and colleagues documented that the patients with late-onset AD displayed higher levels of PCSK9 mRNA and protein in the frontal cortices than healthy individuals.^313^ Additionally, elevated levels of PCSK9 were detected in the CSF, showing a positive correlation with apolipoprotein concentrations in both patients diagnosed with AD and those susceptible to the disease.^313^ PCSK9 was found to be associated with ApoE4 levels in AD patients, while ApoB, ApoE, and ApoJ showed interconnections with PCSK9 in cognitively normal individuals at risk.^313^ These findings indicated that abnormalities in PCSK9 might be detectable prior to the emergence of AD, thereby offering a potential new approach to assess the risk of this disorder. Subsequent studies established links between PCSK9 and well-defined biomarkers for AD, such as Aβ(42), phospho-Tau (p-Tau), and total Tau (t-Tau).^300,313^ In addition, considerable elevation in PCSK9 levels was observed in both individuals with AD and those suffering from other neurodegenerative disorders, relative to patients without these diseases and healthy controls.^309^ This association between PCSK9 and AD’s biomarkers like Aβ (1–42), p-Tau, and t-Tau remained even when adjusting for AD’s diagnosis, pointing towards the possible role of preceding pathways.^309^ Nevertheless, genetic investigations delving into the connection between LOF or GOF variants of PCSK9 and the risk of AD have yet to yield conclusive outcomes.^314–317^

Therefore, given its critical role in modulating lipid homeostasis, PCSK9 might emerge as a potential diagnostic biomarker and therapeutic target for AD, but more long-term, comprehensive, and mechanistic studies are warranted to investigate its potential role in AD.

#### PCSK9 and alcohol use disorder (AUD)

Previous studies have demonstrated that alcohol intake is related to hepatic lipid droplet accumulation, dyslipidemia, altered lipid and lipoprotein levels, and a consequent decline in liver function.^318–321^ It has been discovered that alcohol can enhance PCSK9 expression in the CNS, with CSF PCSK9 levels found to be elevated in patients suffering from AUD. Moreover, a positive relationship was observed between blood and CSF PCSK9 levels in AUD patients, despite the fact that PCSK9 typically struggles to cross the BBB under normal circumstances.^188^ Considering the link between PCSK9 and alcohol as well as metabolic phenotypes, suppressing PCSK9 might alleviate the metabolic effects caused by chronic alcohol consumption. In an experiment using a rat model simulating chronic alcohol consumption, administration of Alirocumab was shown to boost LDLR protein levels and decrease liver inflammation induced by alcohol, implying a crucial role of PCSK9 in these effects.^299^ Future investigations should focus on pinpointing the exact molecular function of PCSK9 and its interaction with alcohol in relation to the pathology of CNS.

### PCSK9 in malignancies

#### The role of PCSK9 in different cancer types

PCSK9, already recognized as an important factor in cholesterol metabolism related to CVD, plays a role in numerous biological processes, as previously discussed.^84^ An increasing body of evidence supports the significant role of PCSK9 in determining cancer prognosis and its abnormal expression in a variety of malignancies. This highlights the potential utility of PCSK9 as a diagnostic biomarker and a viable therapeutic target in cancer.^322,323^ In fact, PCSK9 has been shown to be deeply involved in various cancer-related processes such as cancer cell growth and death, invasion, spread, resistance to radiation, and tumor immunity.^84,322,324^

We initiated our examination by scrutinizing the Cancer Genome Atlas (TCGA) RNA sequencing (RNA-seq) data procured from cbioportal.org.^325,326^ This analysis aimed to reveal the altered PCSK9 expression in cancers and their matched normal samples across a spectrum of different malignancies. It was found that, out of the 17 available cancer datasets, PCSK9 mRNA significantly overexpressed in nine cancer types. These included five cancers of the digestive system: liver hepatocellular carcinoma (LIHC), colon adenocarcinoma (COAD), rectum adenocarcinoma (READ), esophageal carcinoma (ESCA), and stomach adenocarcinoma (STAD). Other cancer types with heightened PCSK9 expression were head and neck squamous cell carcinoma (HNSC), uterine corpus endometrial carcinoma (UCEC), breast invasive carcinoma (BRCA), and thyroid carcinoma (THCA). Conversely, PCSK9 mRNA expression was substantially lower in lung cancers (lung adenocarcinoma [LUAD] and squamous cell carcinoma [LUSC]), kidney cancers (kidney renal clear cell carcinoma [KIRC] and papillary cell carcinoma [KIRP]), and prostate adenocarcinoma (PRAD) (Fig. 2b).

Moreover, many prior studies have already reported abnormal blood PCSK9 levels in patients with various types of cancer including liver, stomach, breast, and thyroid, indicating potential systemic changes in PCSK9 levels in those with cancer.^327–333^ Additionally, several clinical studies have probed the connection between PCSK9 and the prognosis of cancer.^334–338^ In this section, we meticulously evaluate the current body of research that highlights the potential of PCSK9 as a significant biomarker and promising therapeutic intervention for a variety of cancer types, including liver cancer, colorectal cancer (CRC), esophageal and gastric cancer (GC), as well as lung and breast cancer.

LIHC, the second primary cause of cancer-related mortalities throughout the world,^339^ is often correlated with viral hepatitis and cirrhosis. A prior investigation revealed that patients infected with HCV showed augmented blood PCSK9 levels compared to their HCV-negative counterparts. Though a similar tendency was noticed even among LIHC patients, it is noteworthy that PCSK9 levels significantly diminished in patients carrying HCV.^329^ Further, immunohistochemical (IHC) staining of LIHC tissues unveiled considerably lower PCSK9 levels relative to those in the adjacent cirrhotic liver tissues. Interestingly, patients diagnosed with LIHC exhibited markedly higher blood PCSK9 concentrations compared to patients dealing with non-cancerous chronic liver diseases.^330^ These clinical findings somewhat diverge from our analysis utilizing the TCGA-LIHC dataset, which suggested LIHC samples showed substantially higher PCSK9 mRNA expression. This discrepancy might hint at the multifaceted causes and intrinsic heterogeneity of liver cancers. However, it is crucial to note that PCSK9 expression was markedly elevated in five prevalent human liver cancer cell lines, including Bel-7402, Hep3B, HepG2, Huh-7, and SKHep1.^338^ A similar conflict has also been reported among different LIHC clinical analyses. Sun et al. and Zhang et al. asserted that reduced PCSK9 expression, both mRNA and protein, corresponded with improved overall survival (OS) in both studies and disease-free survival (DFS) in Zhang et al.’s study.^336,338^ Conversely, He et al. proposed that heightened PCSK9 expression predicted a better OS and recurrence-free survival (RFS).^340^ These conflicting results underline the necessity for additional fundamental research and larger clinical studies across multiple institutions. This collective effort would contribute to a more definitive examination of PCSK9 as a potential biomarker and target for LIHC.

CRC has a pronounced global prevalence, with colon cancer holding the fifth position among the leading causes of new cancer deaths, while rectal cancer ranks ninth as of 2020.^339^ Our TCGA analyses showed that PCSK9 mRNA expression was substantially elevated in both COAD and READ tumors compared to corresponding normal samples. An extensive analysis of 843 CRC patients by Tao et al. identified *PCSK9* as one of the five differentially expressed genes (DEGs) showing upregulation in CRC samples.^341^ Further, in a study focused on the *APC Regulator Of WNT Signaling Pathway/KRAS Proto-Oncogene, GTPase (APC/KRAS*)-mutant CRC, a particularly therapy-resistant subtype of CRC, Wong et al. also identified *PCSK9* as the top upregulated cholesterol-related gene. They observed that PCSK9 depletion led to a suppression of growth in the *APC/KRAS*-mutant CRC both in cell and animal models, while overexpression of PCSK9 stimulated its carcinogenesis.^342^ This finding was bolstered by an independent tumorigenesis study demonstrating that *APC*^Min/+^ PCSK9 knockin (KI) mice exhibited significantly more and larger CRCs, with 83.3% of these mice developing CRC as opposed to 16.7% of *APC*^Min/+^ mice.^343^ Moreover, several preclinical studies have substantiated that inhibition of PCSK9 through genetic depletion,^34,342,344^ mAbs,^34,343,345,346^ small molecule inhibitor,^342,346^ nanoliposomal vaccine,^347^ or siRNA^348^ could effectively suppress tumor growth. This efficacy was observed when used as a single treatment approach or in combination with immune checkpoint inhibitors (ICIs) in murine spontaneous or subcutaneous CRC models. The cumulative evidence thus points to a compelling potential for targeting PCSK9 in the treatment of CRC in future therapeutic strategies.

In the global context, gastric and esophageal cancers respectively represented the 3^rd^ and 6^th^ leading causes of cancer-related deaths among all 36 cancer types in 2020.^339^ In our TCGA analysis, we observed a significantly elevated mRNA expression of PCSK9 in the tumor tissues of both ESCA and STAD. This elevated expression could potentially underscore its importance in the carcinogenesis of these two cancer types. Ito et al. pinpointed the presence of PCSK9 in the cytoplasm and nucleus of ESCA tissue, a location unoccupied in normal tissue. They also documented a significant surge in serum anti-PCSK9 antibody concentrations in patients grappling with esophageal, gastric, and several other types of cancer. Among these cancers, esophageal cancer showcased the maximum concentration of these antibodies. Intriguingly, esophageal cancer patients manifesting high levels of anti-PCSK9 antibodies demonstrated a superior prognosis after surgery.^337^ In the context of GC, a quantitative proteomic study utilizing stable isotope labeling by amino acids in cellular culture displayed a 13-fold augmentation in PCSK9 expression in the secretome of GC, when compared with non-neoplastic gastric epithelial tissue. This outcome was subsequently validated by IHC staining of GC tumor tissue.^349^ A separate proteomics-focused study echoed these findings, noting plasma PCSK9 concentrations to be 1.25-fold higher in the individuals with early-stage GC compared to those in good health.^328^ In a parallel vein, Xu and colleagues observed that serum PCSK9 concentrations in GC patients were notably higher than in their healthy counterparts. They also registered an upsurge in both PCSK9 mRNA and protein expression in GC cancerous tissues compared to neighboring normal tissues. The higher the PCSK9 expression in these tissues, the poorer the prognosis for the patients.^327^ Collectively, these insights highlight the potential role of PCSK9 as a valuable biomarker for diagnosis and prognosis in esophageal and gastric cancers.

Lung cancer, representing the 2^nd^ most prevalent cancer type, is the leading cause of cancer-related fatalities globally.^339^ In several studies, *PCSK9* has been identified as a critical gene associated with unfavorable prognoses in lung adenocarcinoma.^334,350,351^ Elevated PCSK9 expression has also been reported to be instrumental in promoting the metastasis and invasiveness of lung cancer and melanoma (specifically within the lung).^352,353^ In addition, a clinical cohort study involving 44 elderly patients with non-small-cell lung cancer (NSCLC), who were treated with the ICI, nivolumab, revealed that NSCLC patients with lower levels of blood PCSK9 ( < 95 ng/mL) enjoyed a significantly prolonged OS during the second therapeutic cycle.^354^ These findings were reinforced in a recent report where advanced NSCLC patients exhibiting low baseline plasma PCSK9 levels gained the most benefit from ICI therapy.^355^ In another retrospective analysis involving 115 advanced NSCLC patients receiving anti-programmed cell death 1 (PD-1) treatment, it was found that the PCSK9^lo^ group (patients with low PCSK9 expression) had a significantly longer median progression-free survival (mPFS) than the PCSK9^hi^ group (patients with high PCSK9 expression), 8.1 months versus 3.6 months, respectively, with a hazard ratio (HR) of 3.450 and 95% confidence interval (CI) between 2.166 and 5.496. Additionally, the PCSK9^lo^ group also demonstrated a better objective response rate (ORR) and disease control rate (DCR) compared to the PCSK9^hi^ group (54.4% versus 34.5% and 94.7% versus 65.5%, respectively.^356^ These results emphasize the need for broader investigations into PCSK9 levels, not only in cancer tissues but also in circulation, with an aim to establish PCSK9 as a potential biomarker for the development, progression, and metastasis of lung cancers.

According to the Global Cancer Statistics 2020, breast cancer ranks as the most prevalent cancer worldwide.^339^ A proteomic analysis conducted on a murine model of breast cancer showed a progressive elevation in PCSK9 levels, corresponding with tumor advancement. Interestingly, upon tumor regression, PCSK9 levels returned to almost normal, akin to the control level.^331^ This observation is in harmony with a recent clinical study involving 46 cases, which suggested that PCSK9 levels could escalate in proportion to the severity of breast disease, with the lowest levels found in benign disorders while the highest in stage III breast cancer.^333^ Such evidence accentuates the potential utility of PCSK9 as a diagnostic and prognostic biomarker in breast cancer.

Aside from the aforementioned cancer types, PCSK9 could potentially exert a significant influence across a spectrum of other malignancies. An analysis of the TCGA bladder cancer dataset recognized PCSK9 as a potential biomarker, with a strong correlation to OS of bladder carcinoma patients. The study found that elevated cancerous PCSK9 mRNA expression was associated with worse outcomes.^357^ A separate investigation by Yang et al. observed an upregulation of PCSK9 expression in HNSC tissues, with a higher PCSK9 expression indicative of a poorer prognosis in HNSC patients.^358^ Montero-Calle et al. noted a significant overexpression of PCSK9 in their aggressive endometrial cancer model, indicating a possible role for PCSK9 as a diagnostic biomarker for the disease.^359^In addition, a case-control clinical study illustrated an increase in PCSK9 levels in patients with differentiated thyroid cancer compared to control subjects.^332^ Another examination of ovarian cancer cell lines and patient-derived cancer cells by Sanz et al. proposed that PCSK9 might bolster the survival of ovarian cancer cells, while PCSK9 inhibition could notably impair ovarian cancer survival.^360^ Further, Sun et al. discovered a connection between genetically induced PCSK9 inhibition and a lower risk of prostate cancer.^361^ This result was corroborated by another study that established a robust link between genetically induced PCSK9 inhibition and a decreased risk of both overall and early-onset prostate cancer, where Lp(a) could act as a contributory factor.^362^ Nonetheless, two other studies found that genetically induced PCSK9 inhibition was tied to a higher risk of oral cancer, oropharyngeal cancer, and renal cell carcinoma in men.^363,364^ In addition, Ha et al. hinted at a potential dual role of PCSK9 in the formation of familial pancreatic cancer, proposing that elevated blood PCSK9 levels could be linked to the physiological evolution of precancerous tissue, while reduced PCSK9 levels might be related to progression from lesions to histologically significant lesions.^365^

While the essence of PCSK9 in these cancers still remains unclear, PCSK9 could potentially act as a valuable biomarker for evaluating clinical outcomes in patients with these cancers. Additionally, our earlier Kaplan-Meier survival analyses using TCGA datasets displayed a significant correlation between high PCSK9 mRNA expression and poor OS in various common types of cancer, such as LIHC, pancreatic adenocarcinoma (PAAD), skin cutaneous melanoma (SKCM), bladder urothelial carcinoma (BLCA), LUAD, and ovarian serous cystadenocarcinoma (OV).^34^ These findings suggest that PCSK9 may have a crucial role in carcinogenesis, and a significant number of cancer patients could potentially benefit from PCSK9-iTs.

#### The potential mechanisms of PCSK9 in carcinogenesis and cancer immunology

**Fig. 3:**
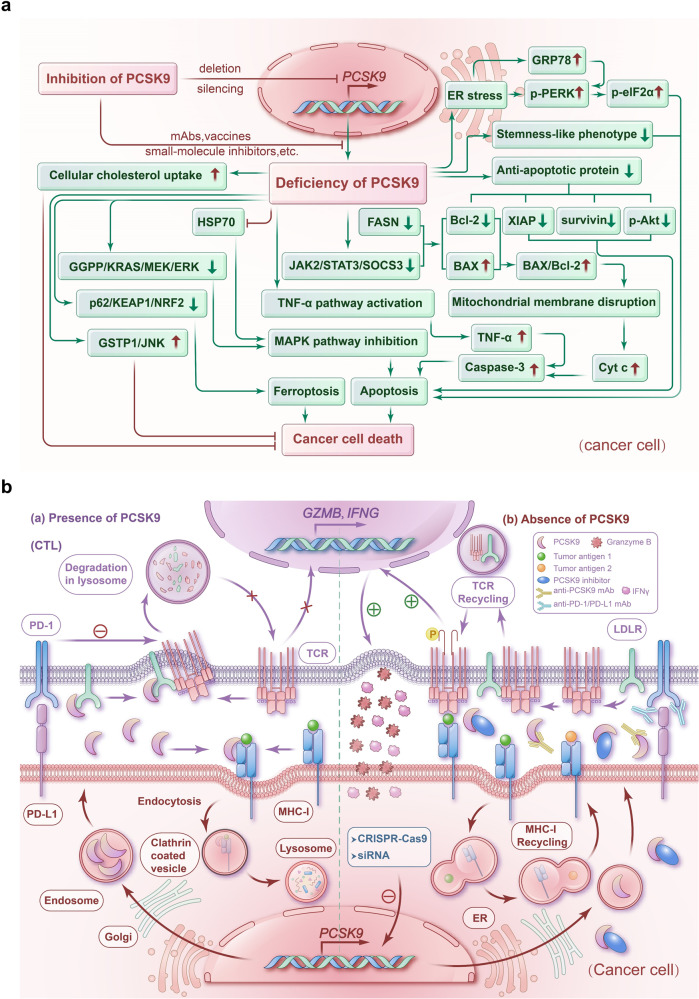
The potential mechanisms of PCSK9 on the regulation of cancer cell death and cancer immunity. **a** PCSK9 deficiency induces ER stress, leading to the dissociation of ER chaperone GRP78 from PERK, which causes downstream phosphorylation of eIF2α to trigger ER stress-induced apoptosis. PCSK9 deficiency can also suppress the development of stemness-like phenotype of cancer. In addition, deficiency of PCSK9 inhibits FA synthase (FASN) or Janus kinase 2/signal transducer and activator of transcription 3/suppressor of cytokine signaling 3 (JAK2/STAT3/SOCS3) pathway to downregulate Bcl-2 levels and upregulate Bax levels to increase Bax/Bcl-2 ratio leading to mitochondrial membrane disruption and subsequent release of cytochrome c (Cyt c) to activate caspase-3 in the cytosol, which can initiate caspase-dependent apoptosis. The caspase-3-initiated apoptotic signaling can also be initiated by the activation of the TNF-α pathway resulting from PCSK9 deficiency. In addition, a deficiency of PCSK9 can downregulate anti-apoptotic proteins X-linked inhibitor of apoptosis protein (XIAP), survivin, and phospho-protein kinase B (p-Akt) to cause cancer cell apoptosis. A deficiency of PCSK9 can also promote cancer cell apoptosis through the inhibition of the MAPK pathway via downregulating heat shock protein 70 (HSP70) levels or the geranylgeranyl diphosphate (GGPP)/KRAS/mitogen-activated extracellular signal-regulated kinase (MEK)/ERK signaling. Moreover, PCSK9 deficiency can downregulate the sequestome 1/Kelch-like ECH-associated protein 1/nuclear factor erythroid 2-related factor 2 (p62/KEAP1/NRF2) signaling pathway to cause cancer cell ferroptosis. However, PCSK9 deficiency may increase LDLR expression and cause cellular cholesterol uptake to increase, or upregulate the Jun N-terminal kinase (JNK) signaling via glutathione S-transferase Pi 1 (GSTP1), which may suppress cancer cell death. **b** (a) PCSK9’s presence can help cancer cells escape from T-cell recognition and elimination. In cancer cells, PCSK9 binds to MHC-I and facilitates its degradation through the endosomal/lysosomal pathway, thereby impeding its recycling to the cell surface. In cytotoxic T lymphocytes (CTLs), PCSK9 binds to LDLR in CTLs, which subsequently binds to the CD3 subunits of the T-cell receptor (TCR) complex and inhibits the recycling of the LDLR-TCR complex to the plasma membrane. Meanwhile, the interaction of programmed cell death 1 (PD-1) and programmed cell death ligand 1 (PD-L1) can drive CTLs to apoptosis or into a regulatory phenotype to lose killing function. (b) Instead, the absence of PCSK9 induced by antibodies, small-molecule inhibitors, or genetic depletion can restore the immune surveillance from CTLs against cancer cells. The recycling of MHC-I of cancer cells and the TCR complex of CTLs can proceed unimpededly, thereby maintaining their elevated levels on the cell surface, respectively. Therefore, cancer cells can more effectively present tumor-specific antigens, which in turn are more readily recognized by CTLs, to perform their antitumor activity. Moreover, if combined with the immune checkpoint inhibitor (ICI) to block the PD-1/PD-L1 axis, PCSK9-iTs can boost an enhanced synergistic antitumor immunity for significant cancer elimination. Panels were illustrated by Adobe Illustrator and Microsoft PowerPoint

The essential hallmarks of cancer are well-established as resistance to cell death and sustaining proliferative signaling.^366^ PCSK9 may play a crucial role in multiple cancer processes, as indicated by studies on lung, neuroglioma, pancreatic neuroendocrine neoplasm (p-NEN), colon, and liver cancers. PCSK9 deficiency seems to inhibit cancer cell proliferation and promote apoptosis through various pathways, including activating caspase-3 and caspase-9, altering the Bcl-2-associated X protein (BAX) / B-cell lymphoma 2 (Bcl-2) ratio to engage mitochondrial apoptotic signaling, and deactivating anti-apoptotic proteins like survivin, X-linked inhibitor of apoptosis protein (XIAP), and phospho-protein kinase B (p-Akt). It also induces ER stress-related factors like glucose-regulated protein (GRP)78, GRP94, phosphorylated protein kinase R (PKR)-like endoplasmic reticulum kinase (p-PERK), and phosphorylated eukaryotic translation initiation factor 2α (p-eIF2α) and influences cholesterol metabolism by regulating FA synthase (FASN) and LDLR.^330,336,343,344,367–369^ (Fig. 3a).

In recent studies on colorectal and liver cancers, more novel roles for PCSK9 in carcinogenesis have been revealed. Wong et al. demonstrated that PCSK9 could boost APC Regulator of WNT Signaling Pathway/KRAS proto-oncogene, GTPase (*APC/KRAS)*-mutant CRC via geranylgeranyl diphosphate (GGPP)-KRAS/ mitogen-activated extracellular signal-regulated kinase (MEK)/ERK signaling pathway, in which PCSK9 deficiency suppressed *APC/KRAS*-mutant CRC both in vitro and in vivo while PCSK9 overexpression promoted carcinogenesis.^342^ Sun et al. uncovered that palmitoylated PCSK9, mediated by zinc finger DHHC-type palmitoyltransferase 16 (ZDHHC16), could direct phosphatase and tensin homolog (PTEN) to the lysosome for degradation, thereby causing sorafenib resistance in HCC. They developed a peptide inhibitor that sensitizes HCC to sorafenib by obstructing PCSK9 palmitoylation, as the currently available PCSK9-iTs do not prevent PCSK9 palmitoylation.^338^ In liver cancer cells (HepG2, Huh6, and Huh7), Alannan et al. found that targeting PCSK9 could induce lipid metabolic exhaustion and cell death through ferroptosis via disruption of the sequestome 1/Kelch-like ECH-associated protein 1/nuclear factor erythroid 2-related factor 2 (p62/KEAP1/NRF2) antioxidative axis.^370,371^ However, another research team stated that PCSK9 could impede cell proliferation, cell cycle progression, and apoptosis by promoting the dissociation of dimers of glutathione S-transferase Pi 1 (GSTP1) and inactivation of the Jun N-terminal kinase (JNK) signaling in HCC, leading to a reduced intracellular cholesterol concentration to suppress HCC cell viability, which could be enhanced by the treatment of *Actinidia chinensis* planch root extract.^340,372^ In prostate cancer (PCa) cells, PCSK9 siRNA demonstrated a protective effect against the cell death induced by ionizing radiation (IR) through reducing apoptosis and inhibiting matrix metalloproteinases (MMPs),^373^ signifying the crucial role of PCSK9-iT in radioresistance in PCa cells. Upon exposure to the liver carcinogen diethylnitrosamine, PCSK9KO mice on HCD exhibited a greater propensity to develop liver cancer in comparison to their WT counterparts.^186^ Further, Yang et al. reported that PCSK9-iTs could notably inhibit the stem-like properties of HNSC cells, an effect that is dependent on the presence of LDLR.^358^ Therefore, further investigation is necessary to demonstrate the potentially critical role of PCSK9 in carcinogenesis and apoptosis, especially for liver cancer. (Fig. 3a).

Aside from these potential roles in carcinogenesis and apoptosis, PCSK9 has been found to orchestrate another fundamental hallmark of cancer: invasion and metastasis.^366^ For example, Bai et al. showed that silencing PCSK9 in the human p-NEN cellular model BON-1 could markedly inhibit invasion.^369^ In a study on mouse melanoma, liver metastases of B16F1 mouse melanoma were cut by half in *Pcsk9*^−/−^ mice compared to WT mice, suggesting that PCSK9 promotes tumor metastasis, which might also be attributed to the apoptotic microenvironment in PCSK9-deficient liver caused by the activation of TNF-α pathway.^374^ In addition, Xu et al. showed that PCSK9 silencing substantially curbed the invasion and migration of GC cells through downregulating phosphorylated levels of several MAPKs, which could be partially reversed by a heat shock protein 70 (HSP70) agonist, while PCSK9 overexpression promoted the invasion and migration of GC cells via the activation of the MAPK signaling. Subsequently, PCSK9KD SGC-7901 GC cells displayed a significantly limited ability to metastasize to the lungs in nude mice, which could also be reversed by the HSP70 agonist.^327^ Similarly, PCSK9KD reduced proliferation, migration, and invasion of colon cancer cells, and inhibited metastasis in vivo. PCSK9 appears to stimulate the progression and metastasis of colon cancer by inducing tumor cell epithelial-mesenchymal transition (EMT) and upregulating the phosphoinositide 3-kinases (PI3K)/p-Akt signaling pathway.^344^ Moreover, a PCSK9-enriched microenvironment induced by either mechanical ventilation or Ahnak could significantly facilitate melanoma cells to metastasize into the lung in mouse B16F10 melanoma models, which could be rescued by genetic deletion of PCSK9 or an anti-PCSK9 mAb.^352,353^ Hence, targeting PCSK9 may be a robust and novel strategy to effectively restrict invasion and metastasis in various cancers. (Fig. 3a).

Over the past twenty years, it has been discerned that cancer has the ability to manipulate tumor-promoting inflammation for its survival, thereby evading immune destruction.^366^ In this context, the role of PCSK9 has been meticulously explored in recent studies due to its multifaceted impact on cancer development. Specifically, PCSK9 can serve as a pivotal factor in the regulation of MHC-I recycling, T-cell receptor (TCR) recycling, and tumor-associated macrophages (TAMs). This underscores the importance of PCSK9 in the orchestration of cancer immunity.

In our previous study, we reported that syngeneic implantation of recipient mice with PCSK9KO mouse melanoma, breast, and colon cancer cells led to substantially suppressed tumor growth compared with PCSK9 vector-control (VC) tumors in mice, which was independent of host LDLR and cholesterol levels.^34^ Similarly, Gu and colleagues also suggested a negative correlation between PCSK9 expression and survival probability in melanoma patients. They further illustrated that PCSK9 promoted the proliferation and migration of B16 mouse melanoma cells along with their growth in vitro and tumor formation in C57BL/6 mice.^375^ Further, PCSK9-iT showed synergistic effects with anti-PD-1 treatment in mouse models, as did treatments with either anti-PCSK9 mAbs (Evolocumab and Alirocumab) or genetic deletion. Flow cytometric analyses revealed an increased intratumoral infiltration by various lymphocytes, including CD8^+^ cytotoxic T lymphocytes (CTLs), CD4^+^ helper T cells, γδ T cells, and natural killer (NK) cells in PCSK9KO B16 melanoma-bearing mice. Additionally, the ratio of CD8^+^ CTLs to Tregs increased in these PCSK9KO tumors. Among those increased CTLs, IFNγ^+^ and granzyme B (GZMB)^+^ subsets were found to be more abundant. Interestingly, when CD8^+^ T cells were depleted, the tumor growth suppression effect from PCSK9 depletion was negated.^34^ Similar findings were reported by Gao et al. in their NSCLC mouse models. The combination of Evolocumab and CD137 agonist significantly prolonged host mouse survival. This effect was associated with a noticeable increase in CD8^+^ and GZMB^+^CD8^+^ T cells and a reduction in Tregs.^356^ In a 4MOSC1 syngeneic mouse HNSC model, inhibiting PCSK9 boosted the infiltration of CTLs in tumor immune microenvironment (TIME) and decreased the myeloid-derived suppressor cells (MDSCs). This action worked in conjunction with anti-PD-1 treatment, creating a synergistic effect.^358^ From a mechanistic viewpoint, we discovered that PCSK9 can reduce the presentation of MHC-I on the tumor cell surface by physically interacting with the complex and guiding them towards lysosomal degradation. PCSK9-iT can prevent this degradation, leading to the increased presentation of tumor antigens for recognition by CTLs, thereby enhancing their cancer-killing activities.^34^ Our understanding of the PCSK9-MHC-I interaction was further supported by a recent study suggesting a potentially crucial role for HLA-C or a similar candidate from the MHC-I family in directing the PCSK9-LDLR complex towards lysosomes for degradation by interacting with PCSK9’s M2 domain.^86^ Therefore, our investigation revealed a potential non-canonical role of PCSK9 in regulating MHC-I levels to modulate TIME, indicating that PCSK9 may be a robust target to enhance cancer immunotherapy (Fig. 3b).

Further, Yuan et al. revealed another significant non-canonical role for the PCSK9/LDLR axis in enhancing CTL-mediated antitumor activity by interacting with CD3 subunits of the TCR complex, affecting TCR recycling, signaling, and overall antitumor capability of CTLs. Overexpression of PCSK9 in tumor cells diminished LDLR and TCR levels in CTLs within TIME, hindering the recycling of the LDLR-TCR complex to the T-cell surface and impeding the CTLs’ functionality. A small-molecule PCSK9 inhibitor counteracted this, enhancing antitumor activity and notably inhibiting tumor progression, especially when combined with anti-PD-1 therapy.^346^ Moreover, a recent report independently validated the combined antitumor efficacy of PCSK9-iT and ICI in CRC models,^345^ whereas another study reiterated that PCSK9 could facilitate melanoma pathogenesis and immune evasion through a network manipulating cancer immunity.^375^ Hence, ensuing in-depth investigations are essential to further explore the distinct PCSK9’s roles in modulating T-cell-mediated antitumor immunity within TIME. (Fig. 3b).

In addition to T cells, previous cardiovascular studies have highlighted the essential role of PCSK9 in inflammation processes, where macrophages, the most abundant effector immune cells, are involved.^84,160–162,376^ Within the context of cancer, macrophages infiltrating TIME are known as tumor-associated macrophages (TAMs), a prevalent type of immune cell in TIME. These TAMs can be categorized into two types: M1-polarized TAMs, which promote anti-tumor responses and cytotoxicity, and M2-polarized TAMs, which facilitate tumor progression and inhibit effective adaptive anti-tumor immunity.^377^ A colon cancer study showed that the M2-polarization of macrophages was inhibited while M1-polarization was promoted by the reduction of lactate, protein lactylation, and the levels of macrophage migration inhibitory factor (MIF) when THP-1-derived macrophages were co-cultured with PCSK9KD HCT116 or HT-29 human colon cancer cells.^344^ However, in a liver cancer study, it was proposed that PCSK9 could suppress the M2-polarization of TAMs through the regulation of OX40L secretion from HCC cells.^378^ Further, in PCSK9KO mice on HCD, a multitude of macrophages were seen amassing around hepatocytes containing cholesterol crystals and lipid droplets, forming crown-like configurations. These structures were accompanied by elevated levels of apoptosis and hepatic inflammation and fibrosis, phenomena not observed in WT mice.^186^ Therefore, it is necessary to investigate the mechanisms of PCSK9 in the regulation of the function and polarization of macrophages in additional cancer models and clinical studies.

## PCSK9 as a potential target for multiple disorders

### PCSK9 as a promising therapeutic target for CVD

Medications that modify lipids are frequently recommended to lower atherogenic lipid quantities and to protect against ASCVD.^379^ Given the significant influence of PCSK9 deficiency upon the significant decrease in blood LDL-C levels, along with the safety demonstrated by the absence or complete lack of PCSK9 in animal models and human studies,^23,69–71,122,125^ it seemed promising that PCSK9-iT could be an effective strategy for LDL-C reduction. Strategies for the development of PCSK9-iT target its synthesis, processing, and binding in order to manage blood levels of LDL-C.^380,381^ Although mAbs and siRNA have been at the vanguard for pioneering the clinical use, many other drug modalities including antisense oligonucleotides (ASO), small-molecule inhibitors, mimetic peptides, adnectin, anticalin, vaccine, meganuclease based gene editing technology, and Clustered regularly interspaced short palindromic repeats (CRISPR)-based gene editing technology have also displayed promising benefits such as greater specificity, potency, and affordability, providing more options for drug administration.^380,382–397^ (Fig. 2c, Table 1).

**Table 1:** Current pharmaceutical strategies to target PCSK9

	Mechanism of action	Advantages	Disadvantages	Administration	Candidates	Development status	Sponsor/Reference
mAbs	To block the PCSK9-LDLR interaction or neutralize PCSK9 activity	High specificity, low toxicity, efficient and safe	Frequent and parental administration, relatively short shelf life, high cost	Subcutaneous injection	Evolocumab	Approved (EMA and US FDA, 2015)	Amgen
					Alirocumab	Approved (EMA and US FDA, 2015)	Sanofi/Regeneron
					Tafolecimab (IBI306)	Approved (China’s NMPA, 2023)	Innovent Biologics (Suzhou)
					Bococizumab	NCT02458287, Phase 3, completed	Pfizer
					Recaticimab (SHR-1209)	NCT04849000, Phase 3, completed	Jiangsu HengRui Medicine
					Ongericimab (JS002)	NCT05325203, Phase 3, recruiting	Shanghai Junshi Bioscience
					Ebronucimab (AK102)	NCT04358432, Phase 2, completed	Akeso/AD Pharmaceuticals
					LY3015014	NCT01890967, Phase 2, completed	Lilly
					RG7652 (MPSK3169A)	NCT01609140, Phase 2, completed	Genentech/Roche
					Lodelcizumab (LGT209)	NCT01859455, Phase 1, completed	Novartis
					MEDI4166	NCT02524782, Phase 1, completed	MedImmune
					Ralpancizumab (PF-05335810)	NCT01720537, Phase 1, completed	Pfizer
					SAL003	ChiCTR2000031373, Phase 1, ongoing	Shenzhen Salubris Pharmaceuticals
siRNA	To target PCSK9 mRNA and inhibit translation	High specificity, infrequent dosing, long-term effect, and safe	Parental injection	Subcutaneous injection	Inclisiran	Approved (EMA, 2020; US FDA, 2021)	Novartis
ASOs	To silence PCSK9 mRNA, leading to its degradation	High specificity	High cost, parental administration	Subcutaneous injection (AZD8233 may be orally available; BMS-844421 can also be intravenously injected.)	AZD8233	NCT04641299, Phase 2, completed	AstraZeneca
					Civi-007	NCT04164888, Phase 2, completed	Civi Biopharma, Inc.
					BMS-844421	NCT01082562, Phase 1, Terminated	BMS
					SPC5001	NCT01350960, Phase 1, Terminated	Santaris Pharma A/S
Small molecules	To block the synthesis and interaction of PCSK9 and enzyme or receptor	Oral administration, easy synthesis, low cost	Low selectivity, non-tissue specific effect, narrow therapeutic window	Orally	CVI-LM001	NCT04438096, Phase 2, Unkonwn status	CVI Pharmaceuticals
					DC371739	NCT04927221, Phase 1, completed	Guangzhou JOYO Pharma
					K-312	NCT02676596, Phase 1, completed	Kowa Research Institute
					SAL092	Phase 1 trial pending in China	Shenzhen Salubris Pharmaceuticals
					DRP	Preclinical	^380^
					NYX-330	Preclinical	^380^
					PF-06446846	Preclinical	^391^
Mimetic peptides	To block PCSK9-LDLR interaction	High specificity, easy synthesis, low cost	Instable in plasma, parental administration	Orally (MK-0616, NNC0385-0434), Subcutaneous injection (Pep2-8)	MK-0616	NCT05261126, Phase 2, completed	Merck Sharp & Dohme
					NNC0385-0434 (NN-6434)	NCT04992065, Phase 2, completed	Novo Nordisk A/S
					Pep2-8	Preclinical	^392,501^
Adnectins	To block PCSK9-LDLR interaction	High specificity, easy synthesis, low cost	short plasma half-life	Subcutaneous injection (Lerodalcibep); Subcutaneous or intravenous injection (BMS-962476)	Lerodalcibep (LIB003)	NCT04790513, Phase 3, completed	LIB Therapeutics/Medpace
					BMS-962476	NCT01587365, Phase 1, completed	BMS
Anticalin	To block PCSK9-LDLR interaction	Ab mimetic but smaller, low cost	Hard to design and screen	Subcutaneous or intravenous injection	DS-9001a	Preclinical	Daiichi Sankyo/Pieris Pharmaceuticals^502^
Vaccines	To induce anti-PCSK9 autoantibodies	Long-term effect, infrequent dosing, easy synthesis, low cost	Autoimmune disorder risk	Subcutaneous injection (AT04A and AT06A); Intramuscular injection (VXX-401)	AT04A	NCT02508896, Phase 1, completed	AFFiRis
					AT06A	NCT02508896, Phase 1, completed	AFFiRis
					VXX-401	NCT05762276, Phase 1, recruiting	Vaxxinity/Novotech
Meganuclease based gene editing technology	To disrupt PCSK9 gene	Infrequent dosing, long-term durable effect	Off-target potential, liver injury, integration of viral vector into the genome	Intravenous injection	AAV-mediated	Preclinical (NHP)	^389,390^
CRISPR based gene editing technology	To disrupt PCSK9 gene	Infrequent dosing, long-term durable effect	Off target potential, liver injury, integration of viral vector into the genome (only for virus mediated editing)	Intravenous injection	Adenovirus based	Preclinical (mouse)	^393^
					AAV-mediated	Preclinical (mouse)	^394^
					LLN-mediated	Preclinical (mouse)	^395^
					LNP-mediated (ABE8.8)	Preclinical (NHP)	^396^
					LNP-mediated (ABEmax)	Preclinical (NHP)	^388^
Natural products	To block the interaction and function of PCSK9 and enzyme or receptor	Oral administration, easy synthesis, low cost	Low selectivity, non-tissue specific effect	Orally	Berberine and monacolin K	NCT03470376, Phase 4, completed	University Of Perugia
					Curcumin, Moracin C, Polydatin, etc.	Preclinical	^397^

*AAV* adeno-associated virus, *EMA* European Medicines Agency, *LLN* lipid-like nanoparticle, *LNP* lipid nanoparticle, *NHP* nonhuman primate, *NMPA* National Medical Products Administration, *US FDA* Food and Drug Administration of the United States

**Fig. 4:**
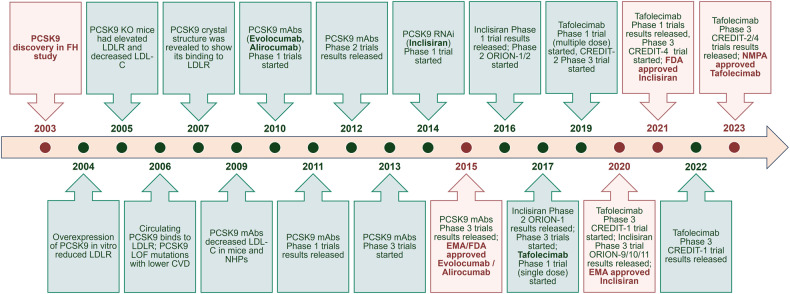
The timeline of the development of PCSK9-iTs. The timeline of the discovery of PCSK9 and the development of its inhibitors. From the initial discovery in 2003, the function of PCSK9 and its inhibitors have been investigated in numerous preclinical and clinical studies over the past two decades, leading to the approval of three specific inhibitors of PCSK9, including three mAbs (Evolocumab and Alirocumab, EMA and US FDA in 2015; Tafolecimab, China’s NMPA in 2023) and one RNA interference (RNAi) (Inclisiran, EMA in 2020, and US FDA in 2021) to treat refractory hyperlipidemia in the clinic. NHP nonhuman primate. Panels were illustrated by Microsoft PowerPoint

Substantial efforts by pharmaceutical industries have resulted in the successful creation of two types of unique, robust, and safe PCSK9-iTs: (i) PCSK9 mAbs (Evolocumab, Alirocumab, and Tafolecimab) that block the interaction between PCSK9 (catalytic domain) and LDLR (EGF-A domain) to negate PCSK9’s activity against LDLR recycling^398–400^ (Fig. 1b), and (ii) siRNA (Inclisiran) that selectively degrades PCSK9 mRNA to suppress its translation and is enclosed in lipid nanoparticles (LNPs) with a specifically hepatic transportation, preventing the synthesis of PCSK9, thus nullifying PCSK9 functions.^401,402^ Further, the chemical structure of Inclisiran was modified to protect against endonucleases, and triantennary N-acetylgalactosamine (GalNAc) was included to augment liver-specific uptake through interaction with the hepatic asialoglycoprotein receptor. This allowed for effective lower dosing.^387^ Evolocumab and Alirocumab were approved by European Medicines Agency (EMA) and the Food and Drug Administration of the United States (US FDA) in 2015,^403^ while Inclisiran was approved by EMA in 2020 and the US FDA in 2021 for clinical use to reduce blood LDL-C level.^404^ Tafolecimab was approved by China’s National Medical Products Administration (NMPA) for the treatment of adult patients with primary hypercholesterolemia and mixed dyslipidemia in August 2023^405^. The mAb method necessitates regular injections every 2 to 6 weeks, which equates to 9 to 26 injections annually, while the siRNA treatment is only needed biannually. Both therapeutic approaches lead to a further 50–60% reduction in LDL-C above that accomplished by the single therapy of statins.^400^^,^^402,403,405^^,406^ Currently, both strategies (Tafolecimab’s long-term safety data pending) appear safe after 2 to 5 years of clinical application,^405^^,407^, however, a comprehensive evaluation of the enduring effects of the reduction of PCSK9 in the liver versus in circulation will require more extended treatment periods. Overall, since the discovery of PCSK9 in 2003, it took less than two decades for four pharmaceutical PCSK9-iTs to be approved for clinical use. This breakthrough is undoubtedly one of the most shining achievements of successful “bench-to-bedside” translational research in modern biomedical history (Fig. 4).

### The emerging of other PCSK9 inhibitors

The development of PCSK9 inhibitors is an inspiring tale of the successful integration of genetics and biotechnology to create highly effective treatments for lowering LDL-C levels. Nevertheless, the clinical implementation into routine care has not progressed as rapidly or smoothly as hoped. A major hindrance is the limited availability of therapies, primarily due to concerns about their cost to healthcare providers.^387^ In addition, oral medications provide the benefit of simpler administration and avoid potential risks and discomfort associated with injections. Therefore, during the past decade, various small molecule compounds have been developed to inhibit the function of PCSK9 (Table 2). For instance, Winston-McPherson et al. discovered a difluoro-2,3’-diindolylmethane (DFDIM) skeleton small molecule inhibitor that can decrease the expression of PCSK9 with half-maximal inhibitory concentration (IC50) ≈ 200 nM in cell line model.^408^ Wang et al. successfully identified an effective small-molecule of PCSK9 inhibitor 7030B-C5 (IC50 = 1.61 μM), which significantly reduced plasma cholesterol and TG levels and retarded atherosclerosis progression in vivo.^61,409^ Nagiec et al. identified a series of novel PCSK9 antagonists such as BRD8518 (half-maximal effective concentration [EC50] = 0.23 μM) by screening the diversity-oriented synthesis (DOS)-derived small-molecule library for compounds that upregulated expression of lipid metabolic gene *TRIB1*.^410^

**Table 2:** Chemical structures of representative small-molecule PCSK9 inhibitors, natural small molecules with PCSK9 inhibitory potential, and peptidomimetic PCSK9 inhibitors

Inhibitor type	Name	Structure	Reference
Small-molecule PCSK9 inhibitor	3 f		^412^
	7030B-C5		^61,409^
	BRD8518		^410^
	Carboxylic acid 9		^411^
	CVI-LM001		^382^
	DC371739		^414^
	DFDIM		^408^
	PF-06446846		^413^
	PF-06815345		^382^
Natural small molecules with PCSK9 inhibitory potential	5a,6a-epoxy-(22E,24 R)-ergosta-8(14),22-diene-3b,7b-diol		^397^
	(2 R,3 R)-3,7,2’-trihydroxy-5-mathoxy-flavanone		^397^
	Adenosine		^397^
	Berberine		^415^
	Curcumin		^415^
	Dihydrocucurbitacin B		^420^
	Erybraedin D		^419^
	Hanabiratakelide A		^397^
	Manglisin E		^397^
	Moracin C		^397^
	Naringin		^424^
	Neolignan		^421^
	Norsesquiterpene		^418^
	Pinostrobin		^397,415^
	Polydatin		^499^
	Pseurotin A		^422^
	Quercelin-3-glucoside		^397^
	Schisandrin C		^397^
	Schisandrol B		^397^
	Skullcapflavone II		^397^
	Sparoside A		^397^
	Tanshinone IIA		^397^
	Vitamin C		^423^
Peptidomimetic PCSK9 inhibitors	13PCSK9i		^427^
	Cyclic-tetramer C12		^428^
	LDLL-1dinr		^426^
	MeIm		^425^
	MK-0616		^382^
	tetra-P9-38		^429^

*Amax* absorption maximum, *EC50* half-maximal effective concentration, *IC50* half-maximal inhibitory concentration, *KD* dissociation constant, *MW* molecular weight

Further, a co-crystal structure-based drug discovery revealed that carboxylic acid 9 (inhibition constant [Ki] = 59 nM) was binding in an allosteric pocket located between the catalytic and C-terminal domain of PCSK9.^411^ Evison et al. identified a compound 3f that disrupted the PCSK9-LDLR interaction at nanomolar levels in vitro (IC50 = 537 nM) and restored LDL uptake in liver cells at sub-micromolar levels.^412^ Pfizer developed compound PF-06446846 (IC50 = 2.2 μM) which was shown to stimulate uptake of LDL-C in hepatoma cells by the mechanism of inhibiting the translation of pro-PCSK9 protein.^413^ Structural modifications were made to improve pharmacokinetic properties and potency, resulting compound PF-06815345 (IC50 = 0.3 μM) which was entered in a clinical trial (NCT02654899), but it was discontinued by Pfizer.^382^ Notably, CVI Pharmaceuticals disclosed a compound CVI-LM001 which reduced LDL-C levels by 26.3%, TC by 20.1%, and ApoB by 17.4% in the phase 1b study (CTR20160744). Currently, CVI Pharmaceuticals is recruiting participants for a phase 2 clinical trial to further assess the effectiveness of CVI-LM001 for hypercholesterolemia treatment (NCT04438096).^382^ Moreover, Wang et al. recently reported a compound DC371739 which suppressed PCSK9 mRNA expression and reduced the plasma levels of total cholesterol (TC), LDL-C, and TG in animal models, and DC371739 showed preliminary positive results in a phase 1 trial (NCT04927221).^414^ (Table 2).

Interestingly, some natural products, such as berberine, erybraedin D, curcumin, schisandrin C, polydatin, sparoside A, naringin, etc., have been reported to suppress the function of PCSK9 directly or indirectly (Table 2).^397,415–418^ For instance, erybraedin D inhibited PCSK9 protein synthesis (IC50 = 7.8 μM) by activating adenosine 5’-monophosphate (AMP)-activated protein kinase (AMPK).^419^ Dihydrocucurbitacin B promoted Dil-LDL uptake in HepG2 cells by upregulating LDLR protein and decreased the serum LDL-C levels in animal model.^420^ Neolignan suppressed PCSK9 mRNA expression (IC50 = 5.13 μM) and increased LDLR expression and LDL-C uptake.^421^ Pseurotin A lowered PCSK9 secretion in HepG2 cells and inhibited the PCSK9-LDLR interaction (IC50 = 1.20 μM).^422^ Vitamin C inhibited PCSK9 expression in cell lines and reduced serum PCSK9 and LDL-R levels in mice by activating FOXO3a and SREBP2.^423^ Naringin down-regulated expression of PCSK9 and decreased plasma 8-isoprostane, fat weight, liver weight, hepatic total cholesterol, hepatic TG, as well as plasma leptin, insulin, and LDL-C in mice model.^424^

Furthermore, several PCSK9-targeted peptidomimetics with high affinity have been developed in recent years (Table 2). For example, Stucchi et al. synthesized a N-methyl tetraimidazole derivative MeIm which inhibited the binding of PCSK9 with LDLR (IC50 = 11.2 μM) and increased LDL-uptake in cells (EC50 = 6.04 μM).^425^ Using the exploring key orientations (EKO) method, Taechalertpaisarn et al. designed and synthesized a peptidomimetic LDLL-1dinr which bound to PCSK9 (Kd = 24.8 μM) and effectively enhanced the LDLR levels and LDL-uptake without cytotoxicity up to 100 μM.^426^ Novartis discovered a potent cyclic peptide 13PCSK9i (dissociation constant [Kd] ≈ 300 pM, EC50 = 0.170 μM, absorption maximum [Amax] = 77% [LDL uptake]) which inhibited PCSK9 with low-nanomolar affinity and reduced plasma TC levels by 43% and enhanced hepatic LDLR by 90% in male C57BL/6 mice.^427^ Merck produced several low-molecular-weight cyclic tetramers such as Cyclic-tetramer C12 which inhibited PCSK9 with an IC50 value of 152 pM in a PCSK9 time-resolved fluorescence resonance energy transfer (FRET) assay.^428^ Tombling et al. designed a tetravalent dendrimer of PCSK9-targeted peptidomimetic tetra-P9-38 (IC50 = 180 pM) which fully restored the LDLR levels and LDL uptake in PCSK9-treated HepG2 cells.^429^ Recently, two peptide PCSK9 inhibitors, MK-0616 (Ki = 2.39 pM) from Merck and NN-6434 (structure undisclosed) from Novo Nordisk, have progressed into phase 2 clinical trials to assess the effectiveness of hypercholesterolemia treatment (NCT05261126, NCT04992065)^382^ (Table 1).

### Strategies of the development of emerging PCSK9 inhibitors

**Fig. 5:**
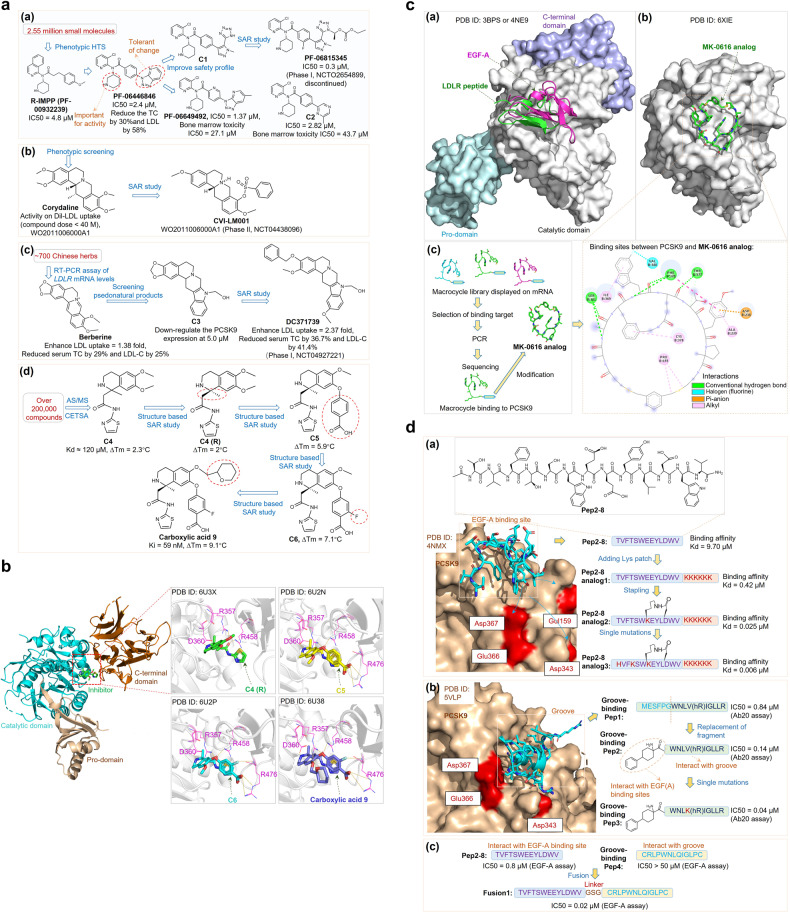
The strategies to develop emerging PCSK9 inhibitors. **a** Strategies for the development of four representative PCSK9 inhibitors: PF-06815345 (a), CVI-LM001 (b), DC371739 (c), and carboxylic acid 9 (d). **b** The crystal structures of PCSK9 with small-molecule inhibitors. Crystal structures were adapted from https://www.rcsb.org/ (PDB ID: 6U3X, 6U2N, 6U2P, 6U38). **c** The co-crystal structures of PCSK9 with EGF-A (pink), LDLR peptide (green) (a) or PCSK9 with MK-0616 analog (b), and the strategy of development of macrocyclic PCSK9 inhibitor from mRNA display (c). Crystal structures were adapted from https://www.rcsb.org/ (PDB ID: 3BPS, 4NE9, 6XIE). **d** The crystal structures of the complex of Pep2–8 (a) and groove-binding Pep1 (b) with PCSK9 and strategies to develop the indicated PCSK9 inhibitors (a-c). Crystal structures were adapted from https://www.rcsb.org/ (PDB ID: 4NMX, 5VLP). AS/MS affinity selection/mass spectrometry, CETSA cellular thermal shift assay, SAR structure-activity relationships, ∆Tm melting temperature shift, Kd dissociation constant, Ki inhibition constant, Ab20 PCSK9-binding antibody 20, EGF-A epidermal growth-factor-like domain A, IC50 half-maximal inhibitory concentration. Panels were illustrated by ChemDraw and Microsoft PowerPoint

Several strategies have been used to develop PCSK9 inhibitors. Of them, phenotypic screening and structure-based drug design are commonest strategies for PCSK9 inhibitor discovery.^382,411,416^ For instance, to search for small molecules that inhibit the function of PCSK9, a phenotypic high-throughput screen was performed in a Chinese hamster ovary (CHO)-K1 cell line overexpressing the recombinant ProLabel-tagged PCSK9. Surprisingly, R-IMPP (PF-00932239) was isolated as a lead compound of anti-PCSK9 from 2.55 million compounds (Fig. 5a-(a)).^413^ To improve pharmacokinetic properties of R-IMPP, structural modifications were made and orally bioavailable PF-06446846 was discovered (Fig. 5a-(a)). To achieve a better safety profile, PF-06815345 and PF-06649492 were subsequently developed by different modifications (Fig. 5a-(a)). PF-06815345 was finally selected as a drug candidate for clinical trial by Pfizer (NCT02654899), but the clinical trial was discontinued due to unknown reasons.^413^ In addition, a promising PCSK9 inhibitor CVI-LM001 (phase 2, NCT04438096) was modified from a natural compound corydaline (Fig. 5a-(b)). CVI-LM001 was identified by phenotypic screening by the mechanism of suppressing *PCSK9* gene expression.^382^ Another drug candidate DC371739 (phase I, NCT04927221) was derived from berberine.^414^ Berberine has been identified from ~700 Chinese herbs through semi-quantitative RT-PCR assay to search compounds that increases in the expression of LDLR mRNA.^430^ DC371739 was subsequently developed by screening pseudonatural products and structure-activity relationships (SAR) studies (Fig. 5a-(c)).^414^

Structure-based drug design is an efficient method to develop PCSK9 inhibitors. Petrilli et al. initially identified a PCSK9 binder C4 (Fig. 5a-(d)) by affinity selection/mass spectrometry (AS/MS) and cellular thermal shift assay (CETSA) from 200,000 compounds.^411^ Fortunately, the X-ray co-crystal structure of C4 bound to PCSK9 was obtained (Fig. 5b). Then, the activities of C4 were improved by a structure-based drug design strategy (Fig. 5a-(d), 5b). The co-crystal structure revealed that R enantiomer but not S enantiomer of C4 strongly interacted with PCSK9 (Fig. 5a-(d), 5b). Adding carboxylic acid group at compound C5 led a salt bridge between R476 of PCSK9, thus the binding ability between C5 and PCSK9 was increased. Subsequently, a hydrogen bond was engendered between R476 when adding a fluoro group to the acid moiety at compound C6 that further improved the stabilization of PCSK9. Finally, carboxylic acid 9 was developed by adding a tetrahydropyran moiety that significantly improved the stabilization of PCSK9 because of the formation of a hydrogen bond between tetrahydropyran oxygen and R357 of PCSK9 (Fig. 5a-(d), 5b).^411^

Remarkably, peptide-based modification is another burgeoning strategy for the development of PCSK9 inhibitors. Since the co-crystal structure of PCSK9/EGF-A and PCSK9/LDLR peptide complex has been obtained (Fig. 5c-(a)),^77,79,431^ various potent peptidomimetic PCSK9 inhibitors have been developed accordingly.^382,432^ Recently, Merck developed an orally bioavailable peptide-based PCSK9 inhibitor MK-0616 derived from an mRNA display selection (Fig. 5c-(b), (c)).^432–435^ Based on the co-crystal structure,^432,433^ MK-0616 or its analogs can form hydrogen bonds with Thr377, Phe379, and Ser381 of PCSK9 protein (Fig. 5c-(c)). MK-0616 inhibited PCSK9 with an IC50 (2.5 ± 0.1 nM) in human plasma and displayed sufficient safety and oral bioavailability in preclinical study.^435^ In a Phase 1 clinical trial in healthy adults, MK-0616 reduced a maximum 61% geometric mean (95% CI, 43%-85%) in LDL-C. MK-0616 was generally well tolerated and no deaths or serious adverse events (AEs) were reported during the study.^435^ In a Phase 2 study in adults with hypercholesterolemia, all doses of MK-0616 demonstrated statistically significant (P < 0.001) differences in least squares mean percentage change in LDL-C from baseline (NCT05261126).^436^

In addition, Pep2-8 that binds to EGF-A binding site of PCSK9 was identified using a randomized linear peptide phage library screening (Fig. 5d-(a)).^382,392^ Interestingly, the modification of Pep2-8 by employing stapled peptide and structure-inducing probes technology produced some potent PCSK9 antagonists (Fig. 5d). The addition of six lysine residues to the C-terminal of Pep2-8 significantly improved the binding affinity because the negatively charged aa residues (Glu159, Asp343, Glu366, and Asp367) on the surface of PCSK9 is near Pep2-8 binding site. The binding affinity was further improved by adding a lactam bridge at Glu7 and Asp11 residues or introducing single mutations at the N-terminal of Pep2-8 (Fig. 5d-(a)). Of note, Zhang et al. reported that a cryptic groove near the EGF-A binding site on PCSK9 is an attractive target site (Fig. 5d-(b)).^437,438^ The groove-binding peptides have been discovered simultaneously.^382^ Surprisingly, connecting the peptides that occupied at groove and EGF-A binding site either by intruding an organic part or by linking two peptides to be a fusion are powerful strategies to develop PCSK9 inhibitors (Fig. 5d-(b), (c)).^382,437,439^ Although several strategies are emerging for discovery of PCSK9 inhibitors, challenges are still existed with the current peptide-based approach for developing efficiently oral anti-PCSK9 drugs.

### Targeting PCSK9 for cancer treatment

Recent investigations have identified PCSK9 as a promising target for the treatment of various malignancies. It has been observed that the inhibition or deficiency of PCSK9 can yield therapeutic benefits in cancer treatment, by not only curbing tumorigenesis but also enhancing anti-tumor immunity.^440,441^ Encouragingly, several therapeutic strategies designed to directly inhibit PCSK9 have already been rigorously explored in the context of hyperlipidemia management. This rigorous investigation resulted in the approval of three mAbs and one siRNA agent that can obstruct both intracellular and extracellular PCSK9 function.^441^ In this segment, we consolidate the existing approaches developed to inhibit PCSK9 in the context of cancer treatment.

#### Anti-PCSK9 mAbs

Two mAbs that neutralize PCSK9, Evolocumab and Alirocumab, have garnered approval from EMA and the US FDA for the clinical intervention of refractory hyperlipidemia, thereby paving the way for utilizing PCSK9 as a target in cancer therapy. Our preceding in vivo experiments illustrated that both mAbs, when combined with anti-PD-1 therapy, elicited a significant synergistic effect in syngeneic mouse models of colon cancer. Approximately 40–50% of the treated tumor-bearing mice demonstrated a long-term complete response (CR), notwithstanding the fact that these mAbs, when used individually, could slow tumor growth. Further, we discovered that both mAbs could revitalize immune checkpoint therapy even after tumor cells had developed resistance to it.^34^ As such, anti-PCSK9 mAbs, used either independently or in combination with ICI or alternative immunotherapies, possess considerable potential for antitumor therapy. Currently, seven clinical trials have been registered to investigate this approach (Table 3).

**Table 3:** Important clinical trials for the development of PCSK9-iTs

PCSK9-iT for CVD	NCT Number	Study Name	Study Title	Phases	Study Status	Conditions	Interventions	Sponsor	Start Date	Primary Completion Date
**Evolocumab** Anti-PCSK9 mAb Approved by EMA and the US FDA in 2015	NCT01516879	DESCARTES	Durable Effect of PCSK9 Antibody CompARed wiTh placEbo Study	3	COMPLETED	Hypercholesterolemia	BIOLOGICAL: Evolocumab BIOLOGICAL: Placebo DRUG: Atorvastatin DRUG: Ezetimibe OTHER: Diet Only	Amgen	Jan-12	Oct-13
	NCT01588496	TESLA	Trial Evaluating PCSK9 Antibody in Subjects With LDL Receptor Abnormalities	2/3	COMPLETED	Homozygous Familial Hypercholesterolemia	BIOLOGICAL: Evolocumab DRUG: Placebo	Amgen	Apr-12	Jan-14
	NCT01624142	TAUSSIG	Trial Assessing Long Term USe of PCSK9 Inhibition in Subjects With Genetic LDL Disorders	2/3	COMPLETED	Severe Familial Hypercholesterolemia	BIOLOGICAL: Evolocumab	Amgen	Jun-12	May-18
	NCT01763827	MENDEL-2	Monoclonal Antibody Against PCSK9 to Reduce Elevated LDL-C in Subjects Currently Not Receiving Drug Therapy for Easing Lipid Levels-2	3	COMPLETED	Hyperlipidemia	BIOLOGICAL: Evolocumab DRUG: Ezetimibe BIOLOGICAL: Placebo to Evolocumab OTHER: Placebo to Ezetimibe	Amgen	Jan-13	Oct-13
	NCT01763866	LAPLACE-2	LDL-C Assessment With PCSK9 Monoclonal Antibody Inhibition Combined With Statin Therapy-2	3	COMPLETED	Hyperlipidemia	BIOLOGICAL: Evolocumab DRUG: Ezetimibe DRUG: Placebo to Evolocumab DRUG: Placebo to Ezetimibe DRUG: Atorvastatin DRUG: Rosuvastatin DRUG: Simvastatin	Amgen	Jan-13	Nov-13
	NCT01763905	GAUSS-2	Goal Achievement After Utilizing an Anti-PCSK9 Antibody in Statin Intolerant Subjects -2	3	COMPLETED	Hyperlipidemia	BIOLOGICAL: Evolocumab DRUG: Placebo to Evolocumab DRUG: Ezetimibe DRUG: Placebo to Ezetimibe	Amgen	Jan-13	Nov-13
	NCT01763918	RUTHERFORD-2	Reduction of LDL-C With PCSK9 Inhibition in Heterozygous Familial Hypercholesterolemia Disorder Study-2	3	COMPLETED	Hyperlipidemia	BIOLOGICAL: Evolocumab DRUG: Placebo	Amgen	Feb-13	Nov-13
	NCT01764633	FOURIER	Further Cardiovascular Outcomes Research With PCSK9 Inhibition in Subjects With Elevated Risk	3	COMPLETED	Dyslipidemia	BIOLOGICAL: Evolocumab DRUG: Placebo	Amgen	Feb-13	Nov-16
	NCT01813422	GLAGOV	GLobal Assessment of Plaque reGression With a PCSK9 antibOdy as Measured by intraVascular Ultrasound	3	COMPLETED	Hypercholesterolemia	BIOLOGICAL: Evolocumab DRUG: Placebo	Amgen	Apr-13	July-16
	NCT01849497	n/a	Study to Assess In-home Use of Evolocumab (AMG 145) Using a Prefilled Syringe or a Prefilled Autoinjector/Pen	3	COMPLETED	Primary Hypercholesterolemia|Mixed Dyslipidemia	BIOLOGICAL: Evolocumab Pre-filled Syringe BIOLOGICAL: Evolocumab AI/pen	Amgen	Apr-13	Sep-13
	NCT01854918	OSLER-2	Open-label Extension Study of Evolocumab (AMG 145) in Adults With Hyperlipidemia and Mixed Dyslipidemia	3	COMPLETED	Hyperlipidemia and Mixed Dyslipidemia	BIOLOGICAL: Evolocumab DRUG: Standard of Care	Amgen	Apr-13	May-18
	NCT01879319	n/a	Study to Assess in Home Use of Evolocumab (AMG 145) Administration Using Either an Automated Mini-doser or a Prefilled Autoinjector/Pen	3	COMPLETED	Primary Hypercholesterolemia|Mixed Dyslipidemia	BIOLOGICAL: Evolocumab AMD BIOLOGICAL: Evolocumab AI/pen	Amgen	Jul-13	Nov-13
	NCT01953328	AMG145	Study of Low-Density Lipoprotein Cholesterol (LDL-C) Reduction Using Evolocumab (AMG 145) in Japanese Patients With Advanced Cardiovascular Risk	3	COMPLETED	Hyperlipidemia or Mixed Dyslipidemia at High Risk for Cardiovascular Events	DRUG: Atorvastatin BIOLOGICAL: Evolocumab OTHER: Placebo to Evolocumab	Amgen	Oct-13	Jun-14
	NCT01984424	GAUSS-3	Goal Achievement After Utilizing an Anti-PCSK9 Antibody in Statin Intolerant Subjects-3	3	COMPLETED	Hyperlipidemia	DRUG: Atorvastatin DRUG: Placebo to Atorvastatin OTHER: Placebo to Ezetimibe DRUG: Ezetimibe OTHER: Placebo to Evolocumab DRUG: Evolocumab	Amgen	Dec-13	Nov-15
	NCT02189837	FLOREY	Effects on Lipoprotein Metabolism From PCSK9 Inhibition Utilizing a Monoclonal Antibody	3	COMPLETED	Primary Hyperlipidemia and Mixed Dyslipidemia	BIOLOGICAL: Evolocumab DRUG: Atorvastatin DRUG: Placebo to Evolocumab DRUG: Placebo to Atorvastatin	Amgen	Jul-14	Feb-15
	NCT02207634	EBBINGHAUS	Evaluating PCSK9 Binding antiBody Influence oN coGnitive HeAlth in High cardiovascUlar Risk Subjects	3	COMPLETED	Dyslipidemia	BIOLOGICAL: Evolocumab DRUG: Placebo DRUG: Background Statin Therapy	Amgen	Sep-14	Nov-16
	NCT02304484	n/a	Open-label Extension (OLE) Study to Assess Safety and Efficacy of Evolocumab	3	COMPLETED	Hypercholesterolemia	BIOLOGICAL: Evolocumab	Amgen	Nov-14	Mar-18
	NCT02392559	HAUSER-RCT	Trial Assessing Efficacy, Safety and Tolerability of Proprotein Convertase Subtilisin/Kexin Type 9 (PCSK9) Inhibition in Pediatric Subjects With Genetic Low-Density Lipoprotein (LDL) Disorders	3	COMPLETED	Heterozygous Familial Hypercholesterolemia	DRUG: Evolocumab DRUG: Placebo	Amgen	Mar-16	Nov-19
	NCT02585895	n/a	Evolocumab Compared to LDL-C Apheresis in Patients Receiving LDL-C Apheresis Prior to Study Enrollment	3	COMPLETED	Hypercholesterolemia	BIOLOGICAL: Evolocumab PROCEDURE: Low-density Lipoprotein Cholesterol (LDL-C) Apheresis	Amgen	Dec-15	Sep-16
	NCT02624869	HAUSER-OLE	Safety, Tolerability and Efficacy of Evolocumab (AMG 145) in Children With Inherited Elevated Low-density Lipoprotein Cholesterol (Familial Hypercholesterolemia)	3	COMPLETED	Familial Hypercholesterolemia	BIOLOGICAL: Evolocumab	Amgen	Sep-16	Jun-21
	NCT02634580	GAUSS-4	Goal Achievement After Utilizing an Anti-PCSK9 Antibody in Statin Intolerant Subjects-4	3	COMPLETED	Hypercholesterolemia	BIOLOGICAL: Evolocumab DRUG: Ezetimibe DRUG: Placebo to Evolocumab DRUG: Placebo Ezetimibe	Amgen	Feb-16	Aug-17
	NCT02662569	BERSON	Safety and Efficacy of Evolocumab in Combination With Statin Therapy in Adults With Diabetes and Hyperlipidemia or Mixed Dyslipidemia	3	COMPLETED	Diabetes, Hyperlipidemia, Mixed Dyslipidemia	BIOLOGICAL: Evolocumab DRUG: Atorvastatin OTHER: Placebo	Amgen	Apr-16	Dec-17
	NCT02729025	ANITSCHKOW	Effects of Proprotein Convertase Subtilisin/Kexin Type 9 (PCSK9) Inhibition on Arterial Wall Inflammation in Patients With Elevated Lipoprotein(a) (Lp(a))	3	COMPLETED	Subjects With Hyperlipidemia, Dyslipidemia	DRUG: Evolocumab DRUG: Placebo	Amgen	Apr-16	Apr-18
	NCT02739984	BANTING	Evaluation of Evolocumab (AMG 145) Efficacy in Diabetic Adults With Hypercholesterolemia/Mixed Dyslipidemia	3	COMPLETED	Hypercholesterolemia Mixed Dyslipidemia Type 2 Diabetes	BIOLOGICAL: Evolocumab DRUG: Placebo to Evolocumab	Amgen	May-16	Aug-17
	NCT02833844	n/a	Safety, Tolerability and Efficacy on Low Density Lipoprotein Cholesterol (LDL-C) of Evolocumab in Participants With Human Immunodeficiency Virus (HIV) and Hyperlipidemia/Mixed Dyslipidemia	3	COMPLETED	Subjects With Hyperlipidemia, Dyslipidemia and HIV Infection	DRUG: Evolocumab DRUG: Placebo	Amgen	May-17	Jul-19
	NCT02867813	FOURIER OLE	Further Cardiovascular Outcomes Research With PCSK9 Inhibition in Subjects With Elevated Risk Open-label Extension	3	COMPLETED	Dyslipidemia	BIOLOGICAL: Evolocumab	Amgen	Sep-16	Mar-22
	NCT03403374	RAMAN	Safety and Tolerability of Repatha®(Evolocumab) in Indian Participants With Homozygous Familial Hypercholesterolemia	4	COMPLETED	Homozygous Familial Hypercholesterolemia HoFH	DRUG: evolocumab	Amgen	Aug-18	Nov-19
	NCT03570697	n/a	Imaging of Coronary Plaques in Participants Treated With Evolocumab	3	COMPLETED	Coronary Artery Disease (CAD)	DRUG: Evolocumab DRUG: Placebo DRUG: Statin therapy	Amgen	Nov-18	Dec-20
	NCT03872401	VESALIUS-CV	Effect of Evolocumab in Patients at High Cardiovascular Risk Without Prior Myocardial Infarction or Stroke	3	ACTIVE_NOT_RECRUITING	Coronary Heart Disease (CHD)	DRUG: Evolocumab DRUG: Placebo	Amgen	Jun-19	Jul-25
	NCT05284747	EVOLVE-MI	EVOLVE-MI: EVOLocumab Very Early After Myocardial Infarction	4	RECRUITING	Cardiovascular Disease Myocardial Infarction Stroke Coronary Revascularization	DRUG: Evolocumab DRUG: Routine Lipid Management	Amgen	Oct-22	Jun-27
**Alirocumab** Anti-PCSK9 mAb Approved by EMA and the US FDA in 2015	NCT01507831	ODYSSEY Long Term	Long-term Safety and Tolerability of Alirocumab (SAR236553/REGN727) Versus Placebo on Top of Lipid-Modifying Therapy in High Cardiovascular Risk Patients With Hypercholesterolemia (ODYSSEY Long Term)	3	COMPLETED	Hypercholesterolemia	DRUG: Placebo (for alirocumab) DRUG: Alirocumab DRUG: Lipid-Modifying Therapy (LMT)	Sanofi/Regeneron	Jan-12	Nov-14
	NCT01617655	ODYSSEY HIGH FH	Efficacy and Safety of Alirocumab (SAR236553/REGN727) Versus Placebo on Top of Lipid-Modifying Therapy in Patients With Heterozygous Familial Hypercholesterolemia (ODYSSEY HIGH FH)	3	COMPLETED	Hypercholesterolemia	DRUG: Alirocumab DRUG: Placebo (for alirocumab) DRUG: Lipid Modifying Therapy (LMT)	Sanofi/Regeneron	Jun-12	May-14
	NCT01623115	ODYSSEY FH I	Efficacy and Safety of Alirocumab (SAR236553/REGN727) Versus Placebo on Top of Lipid-Modifying Therapy in Patients With Heterozygous Familial Hypercholesterolemia Not Adequately Controlled With Their Lipid-Modifying Therapy	3	COMPLETED	Hypercholesterolemia	DRUG: Alirocumab DRUG: Placebo (for alirocumab) DRUG: Lipid Modifying Therapy (LMT)	Sanofi/Regeneron	Jul-12	Apr-14
	NCT01644175	ODYSSEY COMBO I	Efficacy and Safety of Alirocumab (SAR236553/REGN727) Versus Placebo on Top of Lipid-Modifying Therapy in Patients With High Cardiovascular Risk and Hypercholesterolemia (ODYSSEY COMBO I)	3	COMPLETED	Hypercholesterolemia	DRUG: Placebo (for alirocumab) DRUG: Alirocumab DRUG: Lipid-Modifying Therapy (LMT)	Sanofi/Regeneron	Jul-12	Apr-14
	NCT01644188	ODYSSEY COMBO II	Efficacy and Safety of Alirocumab (SAR236553/REGN727) Versus Ezetimibe on Top of Statin in High Cardiovascular Risk Patients With Hypercholesterolemia (ODYSSEY COMBO II)	3	COMPLETED	Hypercholesterolemia	DRUG: Alirocumab DRUG: Placebo (for alirocumab) DRUG: Ezetimibe DRUG: Placebo (for ezetimibe) DRUG: Lipid Modifying Therapy (LMT)	Sanofi/Regeneron	Aug-12	May-14
	NCT01644474	ODYSSEY MONO	Efficacy and Safety of Alirocumab (SAR236553/REGN727) Versus Ezetimibe in Patients With Hypercholesterolemia	3	COMPLETED	Hypercholesterolemia	DRUG: Alirocumab DRUG: Ezetimibe DRUG: Placebo (for Alirocumab) DRUG: Placebo (for Ezetimibe)	Sanofi/Regeneron	Jul-12	Jul-13
	NCT01663402	ODYSSEY Outcomes	ODYSSEY Outcomes: Evaluation of Cardiovascular Outcomes After an Acute Coronary Syndrome During Treatment With Alirocumab	3	COMPLETED	Atherosclerotic Cardiovascular Disease	DRUG: Alirocumab DRUG: Placebo DRUG: LMT	Sanofi/Regeneron	Oct-12	Jan-18
	NCT01709500	ODYSSEY FH II	Study of Alirocumab (REGN727/SAR236553) in Patients With heFH (Heterozygous Familial Hypercholesterolemia) Who Are Not Adequately Controlled With Their LMT (Lipid-Modifying Therapy)	3	COMPLETED	Heterozygous Familial Hypercholesterolemia	DRUG: LMT (atorvastatin, simvastatin, or rosuvastatin) DRUG: alirocumab DRUG: Placebo	Regeneron/Sanofi	#########	May-14
	NCT01709513	ODYSSEY ALTERNATIVE	Study of Alirocumab (REGN727/SAR236553) in Patients With Primary Hypercholesterolemia and Moderate, High, or Very High Cardiovascular (CV) Risk, Who Are Intolerant to Statins (ODYSSEY ALTERNATIVE)	3	COMPLETED	Hypercholesterolemia	DRUG: Atorvastatin DRUG: Ezetimibe DRUG: Alirocumab DRUG: Placebo	Regeneron/Sanofi	Sep-12	May-14
	NCT01730040	ODYSSEY OPTIONS I	Study of the Efficacy and Safety of Alirocumab (REGN727/SAR236553) in Combination With Other Lipid-modifying Treatment (LMT) (ODYSSEY OPTIONS I)	3	COMPLETED	Hypercholesterolemia	DRUG: Alirocumab DRUG: Atorvastatin DRUG: Ezetimibe DRUG: Rosuvastatin DRUG: Placebo	Regeneron/Sanofi	Oct-12	Apr-14
	NCT01730053	ODYSSEY OPTIONS II	Study of Alirocumab (REGN727/SAR236553) added-on to Rosuvastatin Versus Other Lipid Modifying Treatments (LMT) (ODYSSEY OPTIONS II)	3	COMPLETED	Hypercholesterolemia	DRUG: Alirocumab DRUG: Rosuvastatin DRUG: Ezetimibe DRUG: Placebo	Regeneron/Sanofi	Nov-12	Apr-14
	NCT01926782	ODYSSEY CHOICE 1	Study to Evaluate the Efficacy and Safety of an Every Four Weeks Treatment Regimen of Alirocumab (REGN727/ SAR236553) in Patients With Primary Hypercholesterolemia (ODYSSEY CHOICE 1)	3	COMPLETED	Hypercholesterolemia	DRUG: Placebo (for alirocumab) DRUG: Alirocumab DRUG: Statin	Regeneron/Sanofi	Sep-13	Sep-14
	NCT01954394	ODYSSEY OLE	Open Label Study of Long Term Safety Evaluation of Alirocumab	3	COMPLETED	Hypercholesterolemia	DRUG: Alirocumab DRUG: Lipid-Modifying Therapy (LMT)	Sanofi/Regeneron	Dec-13	Jun-17
	NCT02023879	ODYSSEY CHOICE II	Phase III Study To Evaluate Alirocumab in Patients With Hypercholesterolemia Not Treated With a Statin (ODYSSEY CHOICE II)	3	COMPLETED	Hypercholesterolemia	DRUG: Alirocumab DRUG: Placebo (for Alirocumab) DRUG: Non-statin LMT OTHER: Diet Alone	Sanofi/Regeneron	Dec-13	Oct-14
	NCT02107898	ODYSSEY JAPAN	Efficacy and Safety Evaluation of Alirocumab in Patients With Heterozygous Familial Hypercholesterolemia or High Cardiovascular Risk Patients With Hypercholesterolemia on Lipid Modifying Therapy (ODYSSEY JAPAN)	3	COMPLETED	Hypercholesterolemia	DRUG: Placebo (for alirocumab) DRUG: Alirocumab DRUG: Lipid-Modifying Therapy (LMT)	Sanofi/Regeneron	Mar-14	Jan-15
	NCT02289963	n/a	Evaluation of Alirocumab in Addition to Lipid-Modifying Therapy in Patients With High Cardiovascular Risk and Hypercholesterolemia in South Korea and Taiwan	3	COMPLETED	Hypercholesterolemia	DRUG: Placebo (for Alirocumab) DRUG: Alirocumab DRUG: Lipid-Modifying Therapy (LMT)	Sanofi/Regeneron	Jan-15	Apr-16
	NCT02326220	ODYSSEY ESCAPE	Study of Alirocumab (REGN727/SAR236553) in Patients With Heterozygous Familial Hypercholesterolemia (HeFH) Undergoing Low-density Lipoprotein (LDL) Apheresis Therapy	3	COMPLETED	Heterozygous Familial Hypercholesterolemia	DRUG: Placebo DRUG: Alirocumab	Regeneron/Sanofi	Mar-15	Jan-16
	NCT02476006	ODYSSEY APPRISE	Safety, Tolerability, and Effect of Alirocumab in High Cardiovascular Risk Patients With Severe Hypercholesterolemia Not Adequately Controlled With Conventional Lipid-modifying Therapies (ODYSSEY APPRISE)	3	COMPLETED	Hypercholesterolemia	DRUG: ALIROCUMAB SAR236553 (REGN727) DRUG: placebo (for injection training only) DRUG: ezetimibe DRUG: atorvastatin DRUG: rosuvastatin DRUG: simvastatin	Sanofi/Regeneron	Jun-15	Apr-19
	NCT02584504	ODYSSEY-NIPPON	Efficacy and Safety of Alirocumab in Patients With Hypercholesterolemia Not Adequately Controlled With Non-statin Lipid Modifying Therapy or the Lowest Strength of Statin	3	COMPLETED	Hypercholesterolemia	DRUG: Alirocumab DRUG: Placebo DRUG: Atorvastatin DRUG: Non-statin Lipid-Modifying Therapy OTHER: Diet Alone	Sanofi/Regeneron	Nov-15	Apr-17
	NCT02585778	ODYSSEY DM - Insulin	Efficacy and Safety of Alirocumab Versus Placebo on Top of Maximally Tolerated Lipid Lowering Therapy in Patients With Hypercholesterolemia Who Have Type 1 or Type 2 Diabetes and Are Treated With Insulin (ODYSSEY DM - Insulin)	3	COMPLETED	Hypercholesterolemia	DRUG: Alirocumab DRUG: Placebo DRUG: Lipid-Modifying Therapy (LMT) DRUG: Antihyperglycemic Drug	Sanofi/Regeneron	Oct-15	Apr-17
	NCT02642159	ODYSSEY DM-Dyslipidemia	Efficacy and Safety of Alirocumab Versus Usual Care on Top of Maximally Tolerated Statin Therapy in Patients With Type 2 Diabetes and Mixed Dyslipidemia (ODYSSEY DM-Dyslipidemia)	4	COMPLETED	Dyslipidemia	DRUG: Alirocumab DRUG: Statins DRUG: Ezetimibe DRUG: Fenofibrate DRUG: Nicotinic acid DRUG: Omega-3 fatty acids DRUG: Antihyperglycemic Drug	Sanofi/Regeneron	Mar-16	Mar-17
	NCT02715726	ODYSSEY EAST	Evaluation of Alirocumab Versus Ezetimibe on Top of Statin in Asia in High Cardiovascular Risk Patients With Hypercholesterolemia	3	COMPLETED	Hypercholesterolemia	DRUG: Alirocumab DRUG: Placebo for alirocumab DRUG: ezetimibe DRUG: placebo for ezetimibe DRUG: atorvastatin DRUG: rosuvastatin DRUG: simvastatin	Sanofi/Regeneron	Jul-16	Aug-18
	NCT02957682	n/a	Evaluating Effect of the Study Drug Praluent (Alirocumab) on Neurocognitive Function When Compared to Placebo	4	COMPLETED	Hypercholesterolemia	DRUG: Praluent (Alirocumab) DRUG: Placebo	Regeneron/Sanofi	Nov-16	Mar-20
	NCT02984982	ODYSSEY J-IVUS	Evaluation of Effect of Alirocumab on Coronary Atheroma Volume in Japanese Patients Hospitalized for Acute Coronary Syndrome With Hypercholesterolemia	4	COMPLETED	Hypercholesterolemia Acute Coronary Syndrome	DRUG: Alirocumab SAR236553 DRUG: Atorvastatin DRUG: Rosuvastatin DRUG: Fenofibrate DRUG: Bezafibrate DRUG: Ezetimibe DRUG: Antiplatelets DRUG: Anticoagulants	Sanofi/Regeneron	Nov-16	Jul-18
	NCT03156621	ODYSSEY HoFH	Study in Participants With Homozygous Familial Hypercholesterolemia (HoFH)	3	COMPLETED	Homozygous Familial Hypercholesterolemia	DRUG: Alirocumab DRUG: Placebo	Regeneron/Sanofi	Oct-17	Sep-19
	NCT03415178	n/a	Usability Study of the Commercial Auto-injector Device and the New Auto-injector Device (SYDNEY) in Patients With High or Very High CV Risk With Hypercholesterolemia Not Adequately Controlled With Their Lipid-Modifying Therapy	3	COMPLETED	Hypercholesterolemia	DRUG: Alirocumab SAR236553 DEVICE: Current auto-injector device (AI) DEVICE: New auto-injector device (SYDNEY) DRUG: Atorvastatin DRUG: Rosuvastatin	Sanofi/Regeneron	Mar-18	Aug-18
	NCT03510715	n/a	An Efficacy and Safety Study of Alirocumab in Children and Adolescents With Homozygous Familial Hypercholesterolemia	3	COMPLETED	Hypercholesterolemia	DRUG: Alirocumab SAR236553 (REGN727) DRUG: Atorvastatin DRUG: Simvastatin DRUG: Fluvastatin DRUG: Pravastatin DRUG: Lovastatin DRUG: Rosuvastatin DRUG: Ezetimibe DRUG: Cholestyramine DRUG: Nicotinic acid DRUG: Fenofibrate DRUG: Omega-3 fatty acids	Sanofi/Regeneron	Aug-18	Feb-20
	NCT03510884	n/a	An Efficacy and Safety Study of Alirocumab in Children and Adolescents With Heterozygous Familial Hypercholesterolemia	3	COMPLETED	Hypercholesterolemia	DRUG: Alirocumab SAR236553 (REGN727) DRUG: Rosuvastatin DRUG: Atorvastatin DRUG: Simvastatin DRUG: Pravastatin DRUG: Lovastatin DRUG: Fluvastatin DRUG: Ezetimibe DRUG: Cholestyramine DRUG: Nicotinic acid DRUG: Fenofibrate DRUG: Omega-3 fatty acids DRUG: Placebo	Sanofi/Regeneron	May-18	Jan-21
**Inclisiran** A PCSK9-specific siRNA approved by EMA in 2020, and the US FDA in 2021	NCT02314442	n/a	A Phase 1 Study of an Investigational Drug, ALN-PCSSC, in Subjects With Elevated Low Density Lipoprotein Cholesterol (LDL-C)	1	COMPLETED	Hypercholesterolemia	DRUG: ALN-PCSSC DRUG: Sterile Normal Saline (0.9% NaCl)	Alnylam Pharmaceuticals	Dec-14	May-15
	NCT02597127	ORION-1	Trial to Evaluate the Effect of ALN-PCSSC Treatment on Low Density Lipoprotein Cholesterol (LDL-C)	2	COMPLETED	Atherosclerotic Cardiovascular Disease Familial Hypercholesterolemia Diabetes	DRUG: ALN-PCSSC DRUG: Normal Saline	The Medicines Company	Jan-16	Jun-17
	NCT02963311	ORION-2	A Study of ALN-PCSSC in Participants With Homozygous Familial Hypercholesterolemia (HoFH)	2	COMPLETED	Homozygous Familial Hypercholesterolemia	DRUG: ALN-PCSSC DRUG: Standard of Care	The Medicines Company	Dec-16	Oct-18
	NCT03060577	ORION-3	An Extension Trial of Inclisiran in Participants With Cardiovascular Disease and High Cholesterol	2	COMPLETED	Atherosclerotic Cardiovascular Disease Symptomatic Atherosclerosis Type2 Diabetes Familial Hypercholesterolemia	DRUG: Inclisiran DRUG: Evolocumab	Novartis Pharmaceuticals	Apr-17	Dec-21
	NCT03159416	ORION-7	A Study of Inclisiran in Participants With Renal Impairment Compared to Participants With Normal Renal Function (ORION-7)	1	COMPLETED	Renal Impairment	DRUG: Inclisiran	The Medicines Company	Jun-17	Mar-18
	NCT03397121	ORION-9	Trial to Evaluate the Effect of Inclisiran Treatment on Low Density Lipoprotein Cholesterol (LDL-C) in Subjects With Heterozygous Familial Hypercholesterolemia (HeFH)	3	COMPLETED	Heterozygous Familial Hypercholesterolemia Elevated Cholesterol	DRUG: Inclisiran DRUG: Placebo	The Medicines Company	Nov-17	Aug-19
	NCT03399370	ORION-10	Inclisiran for Participants With Atherosclerotic Cardiovascular Disease and Elevated Low-density Lipoprotein Cholesterol	3	COMPLETED	ASCVD Elevated Cholesterol	DRUG: Inclisiran Sodium DRUG: Placebo	The Medicines Company	Dec-17	Sep-19
	NCT03400800	ORION-11	Inclisiran for Subjects With ASCVD or ASCVD-Risk Equivalents and Elevated Low-density Lipoprotein Cholesterol	3	COMPLETED	ASCVD Risk Factor, Cardiovascular Elevated Cholesterol	DRUG: Inclisiran Sodium DRUG: Placebo	The Medicines Company	Nov-17	Jul-19
	NCT03705234	ORION-4	A Randomized Trial Assessing the Effects of Inclisiran on Clinical Outcomes Among People With Cardiovascular Disease	3	ACTIVE_NOT_RECRUITING	Atherosclerotic Cardiovascular Disease	DRUG: Inclisiran DRUG: Placebo	University of Oxford	Oct-18	Jul-26
	NCT03814187	ORION-8	Trial to Assess the Effect of Long Term Dosing of Inclisiran in Subjects With High CV Risk and Elevated LDL-C	3	COMPLETED	ASCVD Elevated Cholesterol Heterozygous Familial Hypercholesterolemia Homozygous Familial Hypercholesterolemia	DRUG: Inclisiran Sodium	Novartis Pharmaceuticals	Apr-19	Feb-23
	NCT03851705	ORION-5	A Study of Inclisiran in Participants With Homozygous Familial Hypercholesterolemia (HoFH)	3	COMPLETED	Homozygous Familial Hypercholesterolemia	DRUG: Inclisiran Sodium for injection DRUG: Placebo DRUG: Placebos	Novartis Pharmaceuticals	Feb-19	Mar-20
	NCT04652726	ORION-16	Study to Evaluate Efficacy and Safety of Inclisiran in Adolescents With Heterozygous Familial Hypercholesterolemia	3	ACTIVE_NOT_RECRUITING	Familial Hypercholesterolemia - Heterozygous	DRUG: Inclisiran DRUG: Placebo	Novartis Pharmaceuticals	Jan-21	Nov-23
	NCT04659863	ORION-13	Study to Evaluate Efficacy and Safety of Inclisiran in Adolescents With Homozygous Familial Hypercholesterolemia	3	ACTIVE_NOT_RECRUITING	Familial Hypercholesterolemia - Homozygous	DRUG: Inclisiran DRUG: Placebo	Novartis Pharmaceuticals	Feb-21	Nov-23
	NCT04666298	ORION-15	Study of Efficacy and Safety of Inclisiran in Japanese Participants With High Cardiovascular Risk and Elevated LDL-C	2	COMPLETED	Hypercholesterolemia Heterozygous Familial Hypercholesterolemia	DRUG: Inclisiran sodium DRUG: Placebo	Novartis Pharmaceuticals	Jan-21	Apr-22
	NCT04765657	n/a	Study of Efficacy and Safety of Inclisiran in Asian Participants With Atherosclerotic Cardiovascular Disease (ASCVD) or ASCVD High Risk and Elevated Low Density Lipoprotein Cholesterol (LDL-C)	3	ACTIVE_NOT_RECRUITING	Hypercholesterolemia	DRUG: inclisiran sodium DRUG: Placebo	Novartis Pharmaceuticals	Mar-21	Jun-22
	NCT04774003	ORION-14	Study of Pharmacokinetics, Pharmacodynamics, Safety and Tolerability of Inclisiran in Chinese Participants With Elevated Serum LDL-C	1	COMPLETED	Hyperlipidemia	DRUG: 100 mg inclisiran sodium (equivalent to 94.5 mg inclisiran) DRUG: Placebo DRUG: 300 mg inclisiran sodium (equivalent to 284 mg inclisiran)	Novartis Pharmaceuticals	Feb-21	Oct-21
	NCT04807400	SPIRIT	Study in Primary Care Evaluating Inclisiran Delivery Implementation + Enhanced Support	3	COMPLETED	Atherosclerotic Cardiovascular Disease Atherosclerotic Cardiovascular Disease Risk Equivelents Elevated Low Density Lipoprotein Cholesterol	DRUG: Inclisiran BEHAVIORAL: Behavioral Support	Novartis Pharmaceuticals	Jul-21	Jan-23
	NCT04873934	VICTORION-INCEPTION	Management of LDL-cholesterol With Inclisiran + Usual Care Compared to Usual Care Alone in Participants With a Recent Acute Coronary Syndrome	3	RECRUITING	Acute Coronary Syndrome	DRUG: Inclisiran	Novartis Pharmaceuticals	Jun-21	Aug-24
	NCT04929249	VICTORION-INITIATE	A Randomized Study to Evaluate the Effect of an “Inclisiran First” Implementation Strategy Compared to Usual Care in Patients With Atherosclerotic Cardiovascular Disease and Elevated LDL-C Despite Receiving Maximally Tolerated Statin Therapy (VICTORION-INITIATE)	3	ACTIVE_NOT_RECRUITING	Atherosclerotic Cardiovascular Disease	DRUG: Inclisiran	Novartis Pharmaceuticals	Jun-21	Sep-23
	NCT05030428	VICTORION-2PREVENT	Study of Inclisiran to Prevent Cardiovascular (CV) Events in Participants With Established Cardiovascular Disease	3	RECRUITING	Atherosclerotic Cardiovascular Disease	DRUG: Inclisiran sodium 300 mg DRUG: Placebo	Novartis Pharmaceuticals	Nov-21	Oct-27
	NCT05192941	VICTORION-DIFFERENCE	Study of Efficacy, Safety, Tolerability and Quality of Life of Inclisiran (KJX839) vs Placebo, on Top of Ongoing Individually Optimized Lipid-lowering Therapy, in Participants With Hypercholesterolemia	4	RECRUITING	Hypercholesterolemia	DRUG: Inclisiran Sodium DRUG: Placebo	Novartis Pharmaceuticals	Apr-22	Feb-25
	NCT05360446	VICTORION-PLAQUE	Coronary Computed Tomography Study to Assess the Effect of Inclisiran in Addition to Maximally Tolerated Statin Therapy on Atherosclerotic Plaque Progression in Participants With a Diagnosis of Non-obstructive Coronary Artery Disease Without Previous Cardiovascular Events	3	RECRUITING	Coronary Artery Disease	DRUG: Inclisiran sodium 300 mg DRUG: Placebo	Novartis Pharmaceuticals	Jul-22	Jan-26
	NCT05399992	VICTORION-REAL	Study Evaluating Effectiveness and Adherence of Inclisiran Plus Standard of Care (SoC) Lipid-lowering Therapy Compared to SoC in ASCVD	n/a	RECRUITING	Primary Hypercholesterolemia Mixed Dyslipidemia	OTHER: Inclisiran	Novartis Pharmaceuticals	Sep-22	Apr-27
	NCT05682378	VICTORION-PEDS-OLE	Long-term Safety and Tolerability of Inclisiran in Participants With HeFH or HoFH Who Have Completed the Adolescent ORION-16 or ORION-13 Studies	3	RECRUITING	Heterozygous or Homozygous Familial Hypercholesterolemia	DRUG: Inclisiran	Novartis Pharmaceuticals	Feb-23	Dec-27
	NCT05739383	n/a	A Study of Inclisiran to Prevent Cardiovascular Events in High-risk Primary Prevention Patients.	3	RECRUITING	Primary Prevention of Atherosclerotic Cardiovascular Disease	DRUG: Inclisiran sodium 300 mg (equivalent to 284 mg inclisiran) in 1.5 mL DRUG: Placebo in 1.5 ml	Novartis Pharmaceuticals	Mar-23	Apr-29
	NCT05763875	VICTORION-Mono	Efficacy and Safety of Inclisiran as Monotherapy in Patients With Primary Hypercholesterolemia Not Receiving Lipid-lowering Therapy.	3	RECRUITING	Hypercholesterolemia	DRUG: Inclisiran DRUG: Ezetimibe DRUG: Matching Placebo for Inclisiran DRUG: Matching Placebo for Ezetimibe	Novartis Pharmaceuticals	Mar-23	Aug-24
	NCT05888103	VICTORION-Mono China	Efficacy and Safety of Inclisiran as Monotherapy in Chinese Adults With Low or Moderate ASCVD Risk and Elevated Low-density Lipoprotein Cholesterol.	3	NOT_YET_RECRUITING	Primary Hypercholesterolemia or Mixed Dyslipidemia	DRUG: Inclisiran DRUG: Matching Placebo for Inclisiran	Novartis Pharmaceuticals	Jul-23	Apr-24
**Tafolecimab** (IBI306) Anti-PCSK9 mAb Approved by China’s NMPA in 2023	NCT03366688	n/a	Single Ascending Dose Study of PCSK-9 Inhibitor (IBI306) in Healthy Subjects.	1	COMPLETED	Hypercholesterolemia	DRUG: IBI306 | DRUG: placebo	Innovent Biologics (Suzhou) Co. Ltd.	Nov-17	Nov-18
	NCT03815812	n/a	Multiple Ascending Dose Study of PCSK-9 Inhibitor (IBI306) in Chinese Patients With Hypercholesterolemia	2	COMPLETED	Hypercholesterolemia	DRUG: IBI306 | DRUG: placebo	Innovent Biologics (Suzhou) Co. Ltd.	Mar-19	Dec-19
	NCT04031742	n/a	A Study to Evaluate Safety and Efficacy of IBI306, a PCSK9 Monoclonal Antibody in Chinese Subjects With Homozygous Familial Hypercholesterolemia	2/3	COMPLETED	Homozygous Familial Hypercholesterolemia	BIOLOGICAL: IBI306 | BIOLOGICAL: IBI306	Innovent Biologics (Suzhou) Co. Ltd.	Sep-19	Dec-21
	NCT04179669	CREDIT-2	Safety and Efficacy of IBI306 in HeFH Patients	3	COMPLETED	Heterozygous Familial Hypercholesterolemia	DRUG: IBI306 | DRUG: placebo	Innovent Biologics (Suzhou) Co. Ltd.	Dec-19	Jun-21
	NCT04289285	CREDIT-1	Safety and Efficacy of IBI306 in Chinese Subjects With Non-familial Hypercholesterolemia	3	COMPLETED	Hypercholesterolemia	DRUG: IBI306 450 mg SC Q4W | DRUG: Placebo SC Q4W | DRUG: IBI306 600 mg SC Q6W | OTHER: Placebo SC Q6W	Innovent Biologics (Suzhou) Co. Ltd.	Apr-20	Feb-22
	NCT04709536	CREDIT-4	A Study of IBI306 in Participants With Hypercholesterolemia	3	UNKNOWN	Hypercholesterolemia	DRUG: IBI306 | DRUG: Placebo	Innovent Biologics (Suzhou) Co. Ltd.	Feb-21	May-21
	NCT04759534	n/a	Application of PCSK9 Inhibitors in Patients With Heterozygous Familial Hypercholesterolemia	3	UNKNOWN	Efficacy and Safety|Heterozygous Familial Hypercholesterolemia|PCSK9	BIOLOGICAL: protein convertase subtilisin/kexin type 9 inhibitor	Shenzhen People’s Hospital	Sep-20	Oct-21
	NCT04948008	n/a	Evaluate the Efficacy and Safety of IBI306 in Subjects With Homozygous Familial Hypercholesterolemia	2/3	UNKNOWN	Familial Hypercholesterolemia - Homozygous|Lipid Metabolism Disorders|Proprotein Convertase Subtilisin/Kexin 9	BIOLOGICAL: IBI306	Shenzhen People’s Hospital	Nov-19	Jul-20
	NCT05792917	n/a	Bioequivalence Study of Tafolecimab Injections in Chinese Healthy Male Volunteers	1	COMPLETED	Healthy Male Subjects	DRUG: tafolecimab (a modified manufacturing process)|DRUG: tafolecimab (a original manufacturing process)	Innovent Biologics (Suzhou) Co. Ltd.	Mar-23	Mar-23
**PCSK9-iT for other disorders**	**NCT Number**	**Study Title**		**Phases**	**Study Status**	**Conditions**	**Interventions**	**Sponsor**	**Start Date**	**Primary Completion Date**
**Infection**	NCT02833844	Safety, Tolerability and Efficacy on Low Density Lipoprotein Cholesterol (LDL-C) of Evolocumab in Participants With Human Immunodeficiency Virus (HIV) and Hyperlipidemia/Mixed Dyslipidemia		3	COMPLETED	Subjects With Hyperlipidemia, Dyslipidemia and HIV Infection	DRUG: Evolocumab DRUG: Placebo	Amgen	May-17	Jul-19
	NCT03139630	COPANA - A09 PCSK 9 Substudy: Impact of Protease Inhibitors on PCSK9 Levels in Naive HIV-Infected Patients		n/a	COMPLETED	HIV Seropositivity Dyslipidemias PCSK9	n/a	Franck Boccara	Mar-16	Sep-16
	NCT03207945	Effect of PCSK9 Inhibition on Cardiovascular Risk in Treated HIV Infection (EPIC-HIV Study)		3	RECRUITING	Dyslipidemias Cardiovascular Diseases HIV Infections	DRUG: Alirocumab OTHER: Placebo	University of California, San Francisco	Apr-18	Jul-24
	NCT03500302	Effect of Evolocumab on Coronary Endothelial Function		2	COMPLETED	Human Immunodeficiency Virus Coronary Artery Disease	DRUG: Evolocumab	Johns Hopkins University	May-18	Nov-19
	NCT03634293	Treatment of Severe Infection With Antihyperlipidemia Drug		2/3	UNKNOWN	Sepsis Septic Shock	DRUG: Alirocumab Injectable Product DRUG: Saline Solution	Wolfson Medical Center	Jan-19	Jan-21
	NCT03869073	Evolocumab for PCSK9 Lowering in Early Acute Sepsis (The PLEASe Study)		2	UNKNOWN	Sepsis	DRUG: Evolocumab DRUG: Placebo	University of British Columbia	Feb-19	Feb-21
	NCT04941105	Impact of PCSK9 Inhibition on Clinical Outcome in Patients During the Inflammatory Stage of the COVID-19		3	COMPLETED	Sars-CoV-2 Infection	DRUG: Evolocumab DRUG: Saline solution	Collegium Medicum w Bydgoszczy	Jun-21	May-22
	NCT05469347	Alirocumab in Patients With Sepsis		1	RECRUITING	Sepsis	DRUG: Alirocumab DRUG: Placebo	Jonathan Sevransky	Jan-23	Apr-24
**Autoimmune disorder**	NCT05191342	Proprotein Convertase Subtilisin Kexin 9 in Rheumatoid Arthritis		n/a	RECRUITING	PCSK9	DIAGNOSTIC_TEST: Enzyme-linked immunosorbent assay for PCSK9	First Affiliated Hospital of Harbin Medical University	Nov-21	Nov-22
**Alcohol use disorder (AUD)**	NCT04781322	Safety, Tolerability, and Bioeffects of Alirocumab in Non-treatment Seeking Heavy Drinkers		1	RECRUITING	Alcohol Associated Liver Disease Heavy Drinking Behavior	DRUG: Alirocumab OTHER: Placebo	National Institute on Alcohol Abuse and Alcoholism (NIAAA)	Oct-21	Dec-23
**Cancer**	NCT03337698	A Study Of Multiple Immunotherapy-Based Treatment Combinations In Participants With Metastatic Non-Small Cell Lung Cancer (Morpheus- Non-Small Cell Lung Cancer)		1/2	RECRUITING	Carcinoma, Non-Small-Cell Lung	DRUG: Atezolizumab DRUG: Cobimetinib DRUG: RO6958688 DRUG: Docetaxel DRUG: CPI-444 DRUG: Pemetrexed DRUG: Carboplatin DRUG: Gemcitabine DRUG: Linagliptin DRUG: Tocilizumab DRUG: Ipatasertib DRUG: Bevacizumab DRUG: Sacituzumab Govitecan OTHER: Radiation DRUG: Evolocumab DRUG: Tiragolumab DRUG: XL092 DRUG: Camonsertib	Hoffmann-La Roche	Jan-18	Aug-25
	NCT04862260	Cholesterol Disruption in Combination With FOLFIRINOX in Patients With Advanced Pancreatic Adenocarcinoma		early 1	RECRUITING	Pancreatic Ductal Adenocarcinoma Pancreatic Cancer Pancreas Cancer Metastatic Cancer	DRUG: Cholesterol metabolism disruption	CHU de Quebec-Universite Laval	Oct-21	Jan-25
	NCT04937413	The PCSK9i Inhibitor Evolocumab - a Surgical Trial of Pharamcodynamics and Kinetics Evaluation		early 1	RECRUITING	Malignant Glioma Glioblastoma	DRUG: Evolocumab	Duke University	Oct-21	Jun-25
	NCT05128539	A Study Explore JS001 + JS002 in Patients With Advanced Cancer		1	RECRUITING	Advanced Cancer	DRUG: JS001(Toripalimab)+JS002	Shanghai Junshi Bioscience Co., Ltd.	Dec-21	Feb-24
	NCT05144529	A Randomized Pilot Study of Evolocumab Plus Nivolumab/Ipilimumab in Treatment-Na??ve Patients With Metastatic NSCLC		2	RECRUITING	Lung Cancer Metastatic	DRUG: Nivolumab DRUG: Ipilimumab DRUG: Evolocumab	Scott Antonia	Mar-22	Dec-23
	NCT05553834	PCSK9 Inhibitor and PD-1 Inhibitor in Patients With Metastatic, Refractory To Prior Anti PD-1 Non-small Cell Lung		2	RECRUITING	Non-small Cell Lung Cancer (NSCLC)	COMBINATION_PRODUCT: Alirocumab and Cemiplimab	Duke University	May-23	Jan-27
	NCT05976893	Study on the Composite Endpoint Event of PCSK9 Inhibitor in Patients With Very High Risk of ASCVD and Cancer		4	NOT_YET_RECRUITING	ASCVD|Cancer	DRUG: Evolocumab DRUG: Statin	Xiang Xie	Aug-23	Dec-25

#### Small-molecule PCSK9 inhibitors

Despite the slower progress of small-molecule PCSK9 inhibitors compared to mAbs, there has been a notable increase in research exploring diverse types of small-molecule PCSK9 inhibitors in recent years, as previously discussed.^397^ Lintner et al. reported the identification of a selective PCSK9 inhibitor, PF-06446846, which was capable of substantially inhibiting PCSK9 translation by inducing a stall in the ribosome around codon 34. This action effectively negated PCSK9 function and led to a significant reduction in bloodstream PCSK9 and the total cholesterol in rats when administered orally.^391^ Further, in a syngeneic mouse MC38 colon cancer model, Yuan et al. demonstrated the synergistic effect of combining PF-06446846 and an anti-PD1 antibody, resulting in marked suppression of tumor growth and a significant extension of OS for the treated mice.^346^ Nonetheless, the clinical applicability of PF-06446846 is constrained by its narrow therapeutic window between PCSK9 lowering and hematopoietic effects. As such, additional efforts are warranted to improve this agent’s safety profile and expand its therapeutic window.

#### Anti-PCSK9 vaccines

This approach aims to prompt the host’s immune response to produce autologous high-affinity anti-PCSK9 antibodies, potentially mimicking the therapeutic effects of externally administered monoclonal antibodies. Advantages of this method include fewer required injections and a decreased risk of unforeseen immune responses to foreign proteins.^386^ Momtazi-Borojeni and team developed a novel anti-PCSK9 vaccine using an immunogenic fused PCSK9-tetanus (IFPT) peptide. This involved combining a small PCSK9 peptide, which serves as a B-cell recognizable epitope, with a tetanus toxin peptide, an epitope for T-helper cells. This combined entity was then bonded to liposomal nanoparticles to enhance the vaccine’s capability to invoke specific immunity significantly.^442^ The anti-PCSK9 vaccine effectively triggered the production of PCSK9-specific antibodies and inhibited PCSK9 levels and functionality in various mouse cancer models. However, it only moderately suppressed tumor growth and marginally extended overall survival, with no significant effect on tumor behavior.^347,443,444^ Further, in a phase 1 clinical trial, Zeitlinger et al. reported that an anti-PCSK9 vaccine, AT04A, demonstrated significant LDL-C lowering activity, accompanied by a safe and immunogenic profile.^445^ Thus, future comprehensive studies should be centered around the optimization of anti-PCSK9 vaccines, amalgamation with optimal adjuvants, and alteration of the immunization scheme, all in a bid to investigate its antitumor efficacy.

#### CRISPR-based PCSK9 gene editing technology

Leveraging innovative technology, PCSK9 depletion in four mouse cancer cell lines significantly reduced their tumorigenic abilities compared to PCSK9VC cells in syngeneic mice. Importantly, reintroducing PCSK9 restored the tumorigenic abilities of PCSK9KO B16F10 cells, negating potential off-target effects of CRISPR-Cas9 editing as the cause of the observed delayed tumor growth.^34^ These experimental findings suggest that PCSK9 depletion using CRISPR-Cas9 technology holds potential as an effective antitumor strategy. In 2014, Ding et al. successfully utilized CRISPR-Cas9 technology to disrupt the *PCSK9* gene in mice with an effective rate exceeding 50%, significantly reducing blood cholesterol levels in mice without any detected off-target mutagenesis in 10 selected sites.^393^ Li et al. employed AAV to deliver CRISPR-Cas9 targeting PCSK9 into C57BL/6 J mice aged 4–6 weeks via tail vein injection, achieving roughly an 80% reduction in PCSK9 and about a 35% decrease in cholesterol levels. Additionally, a 20-fold reduction in off-target activity was noted 24 weeks post-administration, with no observed liver toxicity in the mice.^394^ In addition to the viral delivery system, Jiang et al. reported a non-viral CRISPR-Cas9 gene editing platform using lipid-like nanoparticles (LLNs) for therapeutically targeting hepatitis B virus (HBV) DNA and PCSK9 in mice, leading to a significant decrease of *PCSK9* levels.^395^ Furthermore, a recent report announced that a singular administration of a CRISPR adenine base editor using LNPs into the liver resulted in a significant reduction of both PCSK9 and LDL-C levels (90% and 60%), respectively, which persisted for an eight-month duration in cynomolgus monkeys.^396^ However, before the successful translation of this method into clinical applications for cancer treatment can be realized, it is imperative to conduct more exhaustive preclinical and clinical investigations. These must ensure the safety profile and therapeutic benefits of the technology.

#### PCSK9-specific siRNA

Due to its high specificity and robust gene silencing capabilities, RNA interference (RNAi) has emerged as a crucial approach for tackling a variety of diseases, including infections, cancer, and cardiovascular and neurodegenerative disorders. siRNA molecules enable the targeted and efficient knockdown of genes associated with diseases through post-transcriptional gene silencing.^446^ Xu et al. reported an antitumor effect of PCSK9-specific siRNA in A549 human lung cancer cells, which functions by eliciting mitochondrial-related apoptosis and ER-associated cancer cell death.^367^ A similar apoptotic response in human p-NEN BON-1 cells was reported by Bai et al., resulting from the administration of PCSK9-specific siRNA.^369^ Advancing this novel technique, Guo et al. cleverly developed a method to attach programmed cell death ligand 1 (PD-L1)-binding aptamer PL1 and PCSK9-specific siRNA to precisely constructed DNA tetrahedral nanoparticles (TDNs) via DNA hybridization. This demonstrated a successful and secure treatment alternative for CRC, increasing the infiltration of CTLs and strengthening their ability to produce substantial amounts of IFNγ and GZMB.^348^ In an encouraging advancement, Inclisiran has already received clinical approval for managing hyperlipidemia, though its antitumor efficacy is yet to be evaluated in preclinical or clinical trials. Given its several benefits, such as a low-dose regimen, simple subcutaneous administration, and sustained efficacy, there is a strong rationale to investigate its potential antitumor effectiveness in future studies, either alone or in conjunction with other treatments.^447^

#### Peptide-based PCSK9-iTs

Anti-PCSK9 mAbs have definitively demonstrated significant efficacy in reducing cholesterol levels. Nonetheless, their high cost limits their widespread use, fueling growing interest in alternatives to mAbs. Peptides are one such class of promising therapeutic agents. Like antibodies, they exhibit high specificity and potency toward their targets. Additionally, their smaller size allows for varied administration routes, reduced potential for immunogenic responses, and more cost-effective production.^384^ Zhang et al. reported that, in PCSK9-treated HepG2 liver cancer cells, Pep2–8 reinstated roughly 90% of LDL-C uptake ability, a feat unattained by the control peptide, highlighting its robust capability to neutralize PCSK9’s function.^392^ Currently, two peptide PCSK9-iTs, MK-0616 and NNC0385-0434, are under clinical examination for treating hypercholesterolemia.^382^ In a Phase 2b trial (NCT05261126), MK-0616 showed substantial success, yielding a significant, strong, dose-dependent decrease in LDL-C levels, up to 60.9% from the baseline at eight weeks. The treatment was well-tolerated throughout the 16 weeks, including eight weeks of treatment and an equal duration of follow-up.^436^ However, a thorough investigation into the antitumor efficacy and immunomodulatory effects of these peptide-based PCSK9-iTs is essential within cellular and animal preclinical cancer studies. This would reveal their potential applicability in cancer treatment.

#### Other strategies

Additional therapeutic strategies aiming at PCSK9 inhibition, such as ASOs, adnectin, anticalin, pseurotin A (PS), and various natural products, have been delineated and show profound potential for inhibiting PCSK9 function across cellular and animal cancer models. For instance, the adnectin BMS-962476 efficaciously blocked PCSK9’s biological function by averting its binding and co-internalization with LDLR, thereby reinstating LDLR recycling and amplifying the absorption of LDL in HepG2 liver cancer cells.^448^ PS, a γ-lactam alkaloid sourced from the fungus *Aspergillus fumigatus*, demonstrated not just the useful properties against inflammation and seizure, but also the ability to inhibit PCSK9. Contemporary research revealed the promising potential of orally administered PS to mitigate the progressive hormone-dependent breast cancer or recurrent prostate cancer by curbing PCSK9 levels.^422,449^ While it did not induce acute organ toxicity in mice, PS might incite sex-related toxicity at higher doses.^450^ Additionally, natural compounds such as moracin C, polydatin, tanshinone IIA, as well as an array of flavonoids and flavanones, significantly quell PCSK9 expression in HepG2 cells.^397^ Although a multitude of the therapeutic compounds have already been examined in clinical trials targeting hypercholesterolemia,^382^ the investigation into their prospective antitumor functions warrants further comprehensive cancer studies. Such research endeavors will elucidate their mechanisms of action on carcinogenesis, antitumor immunity, and therapeutic efficacy.

### Landmark clinical trials for the approved PCSK9-iTs against CVD

i) Further Cardiovascular Outcomes Research With PCSK9 Inhibition in Subjects With Elevated Risk (FOURIER), NCT01764633, Completed. (for Evolocumab) (Table 3).

In the randomized, double-blind, placebo-controlled FOURIER trial, 27,564 participants, averaging 62.5 years of age (40–85 years old) with 25% women, who were already on a minimum of 20 mg of atorvastatin with stable ASCVD and additional risk factors, were randomized to either receive Evolocumab (subcutaneous injection, 140 mg biweekly or 420 mg monthly) or a placebo. After 48 months, it was observed that Evolocumab effectively brought down LDL-C to an average of 30 mg/dL which was sustained in subsequent studies. Although a recent clinical study argued that after examining all potential confounding factors, higher levels of anti-PCSK9 autoantibodies were significantly related to the increased deaths among diabetes patients,^451^ Evolocumab has been reported to significantly decrease the risk of recurring CVEs in patients with pre-existing ASCVD and/or T2D.^452,453^ The relative risk (RR) for the main outcome reduced by 15%, with a HR of 0.85 (95% CI 0.79–0.92) over a median 2.2-year monitoring period. A crucial secondary endpoint, the combination of cardiovascular death, MI, or stroke, witnessed a relative decrease of 20%, particularly regarding the risk of MI.^454^ Generally, it was noted that patients with higher cardiovascular incidents, resulting from a variety of genetic cardiovascular risks or severe atherosclerotic diseases, benefitted the most in terms of absolute risk reduction (ARR). A 2.2-year administration of Evolocumab did not escalate the risk of diabetes development or aggravation of glycemia,^453^ corroborating latest studies on either total or β-cell specific PCSK9KO mice, as well as human pancreatic β-cells treated with PCSK9-specific siRNA silencing or Alirocumab.^94,95^ Patients suffering from metabolic syndrome, who had a higher incidence rate compared to those without the syndrome, demonstrated comparable decreases in LDL-C and risks for primary and important secondary outcomes, without an increase in newly developed diabetes, deterioration of the control of blood sugar levels, or any other significant safety complications.^455^ Collectively, during the FOURIER follow-up period, Evolocumab led to significantly reduced LDL-C levels and was not correlated to any AEs. However, although the analyses of the open-label extension of FOURIER (FOURIER-OLE, NCT02867813) showed that in patients with ASCVD, enduring achievement of lower LDL-C levels (<20 mg/dL or <0.5 mmol/L), was linked to a reduced risk of CVEs without any substantial safety issues,^456^ a recent reevaluation after the trial uncovered that the number of cardiac-related deaths was somewhat higher in the Evolocumab group as compared to the placebo controls in the FOURIER trial, which might suggest possible heart harm.^457^ Thus, more attention must be paid to comprehensively examine whether PCSK9-iT will affect homeostasis in healthy tissues. In addition, Evolocumab also led to a median reduction of approximately 27% in Lp(a).^458,459^ Whether this can be attributed to the elevated hepatic LDLR levels induced by Evolocumab, or the decreased secretion of Lp(a) remains a topic of debate.^460–463^

ii) ODYSSEY Outcomes: Evaluation of Cardiovascular Outcomes After an Acute Coronary Syndrome During Treatment With Alirocumab, NCT01663402, completed. (for Alirocumab) (Table 3).

The multicenter, randomized, double-blind, placebo-controlled ODYSSEY Outcomes study engaged 18,924 participants, averaging 59 years of age with 25% being women. This study focused on patients diagnosed as acute coronary syndrome (ACS) 1–12 months prior to randomization, with LDL-C ≥ 70 mg/dL, non-HDL-C ≥ 100 mg/dL, or ApoB ≥ 80 mg/dL.^464^ Participants received atorvastatin 40 mg or rosuvastatin 20 mg and were then divided evenly into two groups: one receiving subcutaneous Alirocumab 75 mg biweekly and the other a placebo. Notably, unlike the FOURIER trial, this study allowed for dosage adjustments. After a 48-month combination treatment of Alirocumab and statins, the mean blood LDL-C concentration decreased to 66 mg/dL. ODYSSEY’s primary endpoint of the trial was defined by a combination of coronary mortality, MI, ischemic strokes, and unstable angina that requires intervention in the hospital. After a median 2.8-year follow-up period, the RR for the primary endpoint of the trial decreased by 15%, corresponding to an HR of 0.85 (95% CI 0.78–0.93). However, no reductions were noted for CAD mortality or other cardiovascular causes, which are similar to the outcomes observed in the aforementioned FOURIER trial utilizing a combination of Evolocumab and statins. Importantly, Alirocumab intervention resulted in a notable 16.2% ARR of death in patients including a disease combination of cerebrovascular, coronary arterial, and peripheral arterial diseases. Lastly, this trial’s extensions suggested that prolonged Alirocumab could potentially lower post-ACS mortality rates, especially in patients at high risk with elevated LDL-C levels.^465^

iii) The ORION program, a worldwide series of clinical studies, explores the therapeutic efficacy and clinical safety of Inclisiran among specific populations, such as those with high-risk ASCVD and diagnosed ASCVD or FH^402,466^ (Table 3). In an initial phase 1 trial (NCT02314442), healthy participants with elevated LDL-C levels > 100 mg/dL received Inclisiran. Administered via subcutaneous injection, doses varied between a single dose (25–800 mg) or multiple doses of 125 to 500 mg, with at least one week’s break between each administration. Remarkable reductions in PCSK9 concentrations (74.5%) after a single 300-mg dose and in blood LDL-C levels (50.6%) after a 500-mg dose at the end of day 84 were reported. Further, all multi-dose regimens exhibited up to a 59.7% and 83.8% reduction in blood LDL-C and PCSK9 levels, respectively. Long-lasting suppressive effects on both LDL-C and PCSK9 levels were observed with doses of 300 mg or higher for at least 180 days. There were no serious adverse events (SAEs) reported in the Inclisiran group compared to placebo.^401^ Following these promising clinical results, a sequence of ORION trials was initiated to delve deeper into Inclisiran’s therapeutic efficacy and clinical safety profiles.^467^ For example, in the ORION-6 and ORION-7 (NCT03159416) trials, Inclisiran’s safety was demonstrated among patients with the malfunction in the liver and kidney, respectively.^468,469^ The ORION-12 trial revealed that Inclisiran could not exert a significant effect on cardiac repolarization.^470^

The ORION-1 clinical trial (NCT02597127), which was the first phase 2 trial for Inclisiran, was a multi-center, double-blind, placebo-controlled trial enrolling 501 high-risk ASCVD patients with elevated blood LDL-C concentrations.^471^ Almost 73% of the recruited participants in this trial were on statin intervention. Patients received a single 200-, 300-, or 500-mg injection of Inclisiran or placebo, or two doses of 100, 200, or 300 mg Inclisriran or placebo (on days 1 and 90). ORION-1’s primary endpoint was defined by the alterations in blood LDL-C concentrations at the 180^th^ day. Depending on the dosage received, reductions in blood PCSK9 and LDL-C levels by the 30^th^ day after the initial injection ranged between 66.2–74.0% and 44.5–50.5%, respectively. On the 180^th^ day, mean decreases in LDL-C levels varied from 27.9–41.9% after a single administration, while 35.5–52.6% following two separate doses. The most considerable reduction in LDL-C levels was observed following the administration of two 300-mg doses. Beyond this, a sustained mean decrease in blood LDL-C concentrations of 26.7–47.2% was recorded at the 240^th^ day, with reductions in both blood PCSK9 and LDL-C levels remaining stable across all dosing schedules. This observation suggested that Inclisiran’s most effective administration was biannual dosing. An additional prespecified analysis indicated that Inclisiran consistently aligned reductions in ApoB and non-HDL-C concentrations in a period of 210 days, with slight decreases observed in VLDL cholesterol (VLDL-C) and TG levels.^472^ AE incidences were comparable between Inclisiran and placebo groups, with SAE incidence rate at 11% and 8%, respectively. Frequently encountered AEs comprised of reactions at the injection site, muscle aches, headaches, tiredness, inflammation of the nasopharynx, back pain, high blood pressure, diarrhea, and lightheadedness. About 4% of participants receiving a single injection together with 7% of those receiving two injections experienced reactions at the injection site.^471^ Additionally, the ORION-3 trial (NCT03060577), a phase 2 OLE of the ORION-1 clinical trial, evaluated Inclisiran’s clinical safety and effectiveness in a long term over a span of 4 years in subjects who were treated with prior Inclisiran administrations in the ORION-1 clinical trial, along with subjects who underwent 1-year treatment with Evolocumab.^473^ The median duration of treatment with Inclisiran from initiation through the ORION-3 clinical study was approximately 4.5 years. Those who took Inclisiran injections twice a year managed to achieve a reduction of 47.5% in blood LDL-C concentrations from the starting point till the 210^th^ day, with the reduction in LDL-C concentrations sustained over the 4-year period without any loss in effectiveness. This outcome exhibited a markedly prolonged effect in comparison to Evolocumab, even though relative reductions in the blood levels of LDL-C and PCSK9 were similar. Throughout the 4-year clinical trial, no new AE patterns emerged.^473^ The ORION-14 (NCT04774003) and ORION-15 (NCT04666298) clinical trials were launched to evaluate the therapeutic effectiveness and clinical safety of Inclisiran treatment in Chinese and Japanese populations, respectively, while the ORION-18 clinical trial (NCT04765657) is underway among Asian participants with ASCVD or ASCVD high risk and elevated LDL-C.

The ORION-10 (NCT03399370) and ORION-11 (NCT03400800) clinical trials were two pivotal phase 3 studies with a double-blind, randomized, placebo-controlled design. They evaluated the percentage change in blood LDL-C levels at a 510-day follow-up and included 1,561 US patients and 1,617 patients from the European Union and South Africa. These patients either had ASCVD plus LDL-C levels ≥70 mg/dL, or ASCVD-risk equivalent plus LDL-C levels ≥100 mg/dL.^474^ The majority of participants in both trials were receiving statin treatment (89.2% in ORION-10 while 94.7% in ORION-11). On the 510th day, blood LDL-C level decreases of 52.3% and 49.9% were observed in the ORION-10 and ORION-11 trials, respectively, for patients treated with Inclisiran. This treatment also led to PCSK9 level reductions of 69.8% and 63.6% in the ORION-10 and −11 clinical trials, respectively. AEs were comparable in both Inclisiran treatment and placebo groups, with the exception that Inclisiran group’s reactions at the injection site were more common to observe.^474^ Furthermore, the ORION-9 clinical trial (NCT03397121) was another important phase 3, double-blind, randomized, placebo-controlled clinical study assessing Inclisiran’s therapeutic effectiveness and clinical safety in 482 patients with heterozygous FH (HeFH). Even though all HeFH patients were receiving the maximum tolerable dose of statin treatment, with ezetimibe or not, the average baseline blood LDL-C concentration was 153 mg/dL. On the 510th day, LDL-C levels decreased by an average of 39.7% in Inclisiran-treated patients compared to an 8.2% increase in the placebo controls. A genotype-based HeFH substudy indicated consistent LDL-C level reductions across those HeFH patients displaying all types of different genetic defects. The incidences of AEs and SAEs were similar in both groups.^475^ A summary of the ORION-9, −10, and −11 clinical trials for all the patients at very high risk showed that Inclisiran treatment decreased blood concentrations of LDL-C, ApoB, and non-HDL-C by 51%, 42%, and 46%, respectively, corresponding to a 24% reduction in CVE rate in the subanalysis.^476,477^ A meta-analysis of five randomized controlled Inclisiran clinical trials also reported optimistic impacts on various indicators for lipids and lipoproteins as well as a robust clinical safety profile of Inclisiran.^478^ The long-term Inclisiran’s tolerability and effectiveness were corroborated by phase 3 clinical data summarized from the ORION-9, −10, and −11 clinical trials, which facilitated Inclisiran’s approval by EMA in 2020 and the US FDA in 2021 for the treatment of primary hypercholesterolemia or mixed dyslipidemia.^479^

The ORION-2 trial (NCT02963311), a phase 2 proof-of-concept, open-label, single-arm, multicenter pilot study was designed to determine the dosing and treatment plan for the following ORION-5 clinical trial (NCT03851705). This study focused on adult patients diagnosed with homozygous FH (HoFH).^480^ The outcome of the ORION-2 trial was that all four patients experienced substantial and sustained decreases in blood PCSK9 levels (48.7–83.6% on the 90th day while 40.2–80.5% on the 180th day, respectively). Notably, three out of the four patients also observed substantial decreases in LDL-C levels (11.7–33.1% at the 90th day while 17.5–37.0% on the 180th day, respectively). The trial did not report any treatment-associated AEs or reactions at the injection site.^85^ The ORION-5 is an ongoing two-part clinical trial (double-blind placebo-controlled as well as open-label) being conducted across multiple centers. It aims to assess Inclisiran’s clinical safety, effectiveness, and tolerability among 56 HoFH patients. Given that reductions in blood LDL-C and PCSK9 levels were observed among HoFH patients who were administered 300 mg of Inclisiran without any adjustments to the dosing or regimen, the ORION-5 trial has proceeded using the standard dose and regimen to evaluate Inclisiran’s effects, tolerability, and safety in the long term. ORION-5’s outcomes are pending publication. In addition, the ORION-13 (NCT04659863) and ORION-16 (NCT04652726) clinical trials, which aim to evaluate the therapeutic effectiveness and clinical safety of Inclisiran in 12–17 years old adolescents diagnosed with HoFH and HeFH, respectively, are currently underway.

The ORION-8 clinical trial (NCT03814187) is another ongoing, global, multicenter study extending from the four ORION-3, −9, −10, and −11 clinical trials. It aims to evaluate Inclisiran’s long-term effectiveness and clinical safety until the 990^th^ day across 2,991 subjects with elevated blood LDL-C levels and an elevated cardiovascular risk. A network meta-analysis of non-statin treatments to reduce lipids illustrated that while anti-PCSK9 mAbs could reduce blood LDL-C concentrations by 64.7% (95% CI 67.4–62.0%), Inclisiran intervention was able to turn down blood LDL-C levels by 50.2% (95% CI 55.0–45.4%). Although anti-PCSK9 mAbs were revealed to be more efficacious at reducing the levels of blood LDL-C, Inclisiran treatment was projected to deliver a comparable improvement in blood LDL-C levels.^481^ When used in combination with maximally tolerated statin treatment along with ezetimibe, Inclisiran facilitated a reduction of over 80% in blood LDL-C levels, similar to the effectiveness of anti-PCSK9 mAbs.^482,483^ Moreover, the ORION-4 clinical trial (NCT03705234), an ongoing double-blind, randomized, placebo-controlled phase 3 clinical trial, has recruited roughly 15,000 patients with established ASCVD across about 180 clinical centers in the US and United Kingdom. This trial aims to assess Inclisiran’s effect on cardiovascular outcomes over a median 5-year monitoring period. Its primary completion date is expected to be in July 2026. Its results are eagerly anticipated worldwide as they will offer valuable insights into Inclisiran’s clinical benefits in preventing CVEs.

At present, the VICTORION series of clinical trials, which includes part of the ORION clinical studies, are in progress to evaluate Inclisiran’s impact on the lifespan of the patients at high risk.^484^ The VICTORION-2PREVENT clinical trial (NCT05030428), a phase 3 trial for established ASCVD patients in several states, aims to evaluate the ability of Inclisiran to decrease the incidence of 3-point-major adverse CVEs, determined with a combination of cardiovascular mortality as well as non-fatal MI and ischemic stroke. Several other ongoing clinical trials, including VICTORION-INCEPTION (NCT04873934), VICTORION-INICIATE (NCT04929249), VICTORION-REAL (NCT05399992), VICTORION-DIFFERENCE (NCT05192941), and VICTORION-SPIRIT (NCT04807400), aim to expand Inclisiran’s diverse applications in several different clinical scenarios^485^ (Table 3).

To date, Inclisiran has displayed promising results in all conducted trials, demonstrating a decrease in both blood LDL-C and PCSK9 levels plus a satisfactory safety feature. Several forthcoming clinical trials are expected to reveal insights into Inclisiran’s therapeutic effects and clinical benefits in the long term.

iv) The CREDIT program, a series of clinical studies, explores the therapeutic efficacy and clinical safety of Tafolecimab (IBI306) among Chinese population, such as those with familial or non-familial hypercholesterolemia^405^ (Table 3). In two initial phase 1 studies (Single Ascending Dose Study of PCSK-9 Inhibitor [IBI306] in Healthy Subjects, NCT03366688 and Multiple Ascending Dose Study of PCSK-9 Inhibitor [IBI306] in Chinese Patients With Hypercholesterolemia, NCT03815812), fifty-eight healthy volunteers (phase 1a) received a single dose of Tafolecimab at 25, 75, 150, 300, 450, or 600 mg subcutaneously, 75 or 450 mg intravenously, or placebo. Sixty patients with hypercholesterolemia (phase 1b) received Tafolecimab at 75 or 140 mg every 2 weeks (Q2W), 300 or 420 mg Q4W, 450 or 600 mg Q6W subcutaneously, or placebo for 12 weeks. Tafolecimab was well tolerated with mild to moderate AEs in both cohorts. Phase 1a saw up to 72% reduction in LDL-C levels with a single dose in healthy volunteers, while Phase 1b showed consistent reductions exceeding 50% in LDL-C levels across all dose regimens up to week 12 in patients. Tafolecimab has thus manifested as a safe PCSK9 mAb, showcasing substantial and lasting LDL-C–lowering potential.^486^

The CREDIT-1 clinical trial (NCT04289285), a phase 3, double-blind, multicenter, randomized, placebo-controlled study, assessed Tafolecimab in Chinese subjects with non-familial hypercholesterolemia who are at high or very high cardiovascular risk. Patients were assigned in a 2:2:1:1 ratio to receive subcutaneous Tafolecimab 450 mg Q4W, Tafolecimab 600 mg Q6W, placebo Q4W, or placebo Q6W for 48 weeks, involving 618 patients with an average LDL-C level of 2.85 mmol/L (9.3% on ezetimibe, and 72.8% at very high cardiovascular risk). Tafolecimab induced notable reductions in LDL-C levels at both dose levels, outperforming the placebo in achieving ≥50% LDL-C reductions, LDL-C < 1.8 mmol/L, and LDL-C < 1.4 mmol/L. In addition, significant reductions in non-HDL cholesterol, ApoB, and Lp(a) levels were also recorded compared to the placebo at week 48. The most prevalent treatment-emergent AEs included upper respiratory tract infection, urinary tract infection, and hyperuricemia.^487^ These results demonstrated Tafolecimab treatment at either dosage led to significant and durable reductions in LDL-C levels together with a robust safety profile in Chinese patients with non-familial hypercholesterolemia.

The CREDIT-2 clinical trial (NCT04179669) was a randomized, double-blind, placebo-controlled phase 3 trial to examine the efficacy and safety of Tafolecimab in Chinese patients with HeFH. Patients diagnosed with HeFH and on a stable lipid-lowering therapy for at least 4 weeks were randomized 2:2:1:1 to receive subcutaneous Tafolecimab 150 mg Q2W, 450 mg Q4W, placebo Q2W, or placebo Q4W for 12 weeks, with a subsequent open-label 150 mg Q2W or 450 mg Q4W for another 12 weeks. 149 participants were randomized and 148 received at least one dose of the study treatment. At week 12, Tafolecimab treatment induced significant reductions in LDL-C levels (−57.4% [97.5% CI, −69.2 to −45.5] for 150 mg Q2W; −61.9% [−73.4 to −50.4] for 450 mg Q4W; both P < 0.0001), outperforming the placebo in achieving ≥50% LDL-C reductions or LDL-C < 1.8 mmol/L at week 12. In addition, significant reductions in non-HDL cholesterol, ApoB, and Lp(a) levels were also recorded compared to the placebo at week 12. The lipid-reducing efficacy of Tafolecimab persisted through the 24^th^ week. During the double-blind treatment phase, AEs reported most frequently for the Tafolecimab treatment encompassed upper respiratory tract infection, elevated levels of blood creatine phosphokinase, elevated alanine aminotransferase, elevated aspartate aminotransferase, and hypertension. Therefore, administering Tafolecimab, whether 150 mg Q2W or 450 mg Q4W, resulted in significant and sustained reductions in LDL-C levels, demonstrating a favorable safety profile for Chinese HeFH patients.^400^

The CREDIT-4 clinical trial (NCT04709536) was another randomized, double-blind, placebo-controlled phase 3 trial to assess the efficacy and safety of Tafolecimab in Chinese patients at high or very high cardiovascular risk with hypercholesterolemia. A total of 303 patients diagnosed with HeFH or at high or very high cardiovascular risk with non-familial hypercholesterolemia (LDL-C level ≥ 1.8 mmol/L) were randomized 2:1 to receive at least 1 dose of Tafolecimab (n = 205) or placebo (n = 98) 450 mg Q4W for 12 weeks. The least squares mean percent change in LDL-C level from baseline to week 12 was –68.9% (SE 1.4%) and –5.8% (1.8%) in the Tafolecimab and placebo group (P < 0.0001), respectively. Tafolecimab outperformed the placebo in achieving ≥50% LDL-C reductions or LDL-C < 1.8 mmol/L, and LDL-C < 1.4 mmol/L at week 12 (all P < 0.0001). Furthermore, substantial reductions in non-HDL cholesterol, ApoB, and Lp(a) levels were also observed compared to the placebo at week 12. Urinary tract infection (5.9% with Tafolecimab vs 4.1% with placebo) and hyperuricemia (3.4% vs 4.1%) were the most reported AEs during the double-blind treatment phase. This trial confirmed the safety and robust lipid-lowering efficacy of Tafolecimab in Chinese patients at high or very high cardiovascular risk with hypercholesterolemia.^406^

### Clinical trials for PCSK9-iTs against other disorders

In addition to the clinical trials that examine the therapeutic efficacy of PCSK9-iTs for CVD, there are also a dozen of ongoing trials to investigate the emerging effect of PCSK9-iTs for other aforementioned disorders (Table 3). For example, a phase 3 clinical trial (EPIC-HIV Study, NCT03207945) is currently underway to examine Alirocumab’s effects on reversing coronary endothelial damage and reducing the inflammation in the artery among HIV patients, with results expected in July 2024. Meanwhile, the PLEASe Study (NCT03869073), Treatment of Severe Infection With Antihyperlipidemia Drug (NCT03634293), and PALMS (NCT05469347), are aiming to ascertain if quick bacterial component removal via PCSK9 neutralization with Evolocumab or Alirocumab proves beneficial. Moreover, the trial, Proprotein Convertase Subtilisin Kexin 9 in Rheumatoid Arthritis (NCT05191342), will assess the expression and significance of PCSK9 in RA patients, while the study, titled “Safety, Tolerability, and Bioeffects of Alirocumab in Non-treatment Seeking Heavy Drinkers” (NCT04781322), is projected to conclude its primary phase in December 2023 to measure the impact of PCSK9-iT on heavy drinkers. Furthermore, seven ongoing trials will help determine the therapeutic potential of PCSK9-iTs as an emerging antitumor strategy, including four trials examining the combination of anti-PCSK9 mAb with ICI in NSCLC and advanced cancers (NCT03337698, NCT05128539, NCT05144529, and NCT05553834), one trial investigating the potential efficacy of the combination of anti-PCSK9 mAb with chemotherapy in advanced pancreatic adenocarcinoma (NCT04862260), one surgical trial examining the pharmacodynamics and kinetics of Evolocumab in malignant glioma patients (NCT04937413), and one trial to study the composite end event of PCSK9 inhibitor in patients with very high risk of ASCVD and cancer (NCT05976893).

### Potential pitfalls of PCSK9-iTs

#### Common AEs

As previously mentioned, four PCSK9-iTs are presently sanctioned for clinical application: Alirocumab and Evolocumab, which received first approval in 2015, Inclisiran approved in 2020, and Tafolecimab, most recently approved in 2023. Given the earlier approval of Alirocumab and Evolocumab, there is a more extensive array of clinical data available to track their AEs. A real-world report analyzed three datasets including a hospital registry (*n* = 164) and two Pharmacovigilance databases, Lareb (*n* = 149) and VigiLyze (*n* = 15,554) to reveal AEs attributed to clinically prescribed Alirocumab or Evolocumab. The hospital registry noted 41.5% of patients experiencing an AE, primarily injection-site reactions (33.8%) and influenza-like illness (27.9%), with 7% discontinuing treatment. The predominant AE reported in Lareb and VigiLyze was myalgia (12.8% and 8.3%, respectively). There were no notable differences in gender or between drugs, and no specific patient subgroup at a higher risk of developing AEs was identified. A majority (71.1%) saw resolution of AEs during follow-up without extra specific treatment. Thus, in everyday clinical settings, PCSK9 inhibitors maintain a safety profile aligning with randomized controlled trial (RCT) findings, showcasing their tolerability.^488^ As displayed in the above clinical trials, the overall AE rate in the patients who received Inclisiran and Tafolecimab was similar to the placebo group, while the most common AEs were comparable to those two early mAbs. However, their long-term AE, especially for Inclisiran, has to be carefully monitored and recorded during their clinical use. Therefore, regular monitoring and follow-ups are essential to detect any AEs early and manage them appropriately. Meanwhile, educating patients about the potential side effects and advising them to report any unusual symptoms promptly can enhance patient safety.

#### Uncommon AEs

Per the prescribing information from the US FDA, hypersensitivity reactions such as rash, pruritus, and urticaria have been documented in association with PCSK9 inhibitors, with instances of severe allergic reactions being exceptionally infrequent. Alirocumab has led to the development of drug-neutralizing antibodies in approximately 1.2% of patients, yet this occurrence did not exhibit a consistent correlation with a reduction in efficacy in diminishing LDL-C levels. During clinical trials, no detection of neutralizing antibodies to Evolocumab was reported. While minor elevations in neurocognitive symptoms have been observed in a few trials with the administration of Alirocumab or Evolocumab compared to placebo,^489,490^ a meticulous sub-study within the FOURIER trial, encompassing 1974 patients, revealed no substantial evidence of neurocognitive deterioration.^491^ Concerning musculoskeletal adverse events (MAEs), a study by Ding et al. disclosed an association between the utilization of anti-PCSK9 mAbs (Alirocumab or Evolocumab) and the emergence of MAEs. This risk was notably amplified when PCSK9 inhibitors were amalgamated with statins.^492^

Additionally, theoretical conjectures suggest a potential influence of PCSK9 on HCV infectivity by modulating entry receptors. PCSK9 antibodies could conceivably facilitate HCV entry into hepatocytes by elevating the expression of LDLR and CD81 on their surface.^204^ However, the current corpus of evidence is inconclusive, necessitating further investigative endeavors. Moreover, despite the theoretical postulation that elevated bile acid levels^493^—induced by more cholesterol uptake by the liver due to the upregulation of LDLR by PCSK9 antibodies—could stimulate bile acid production, leading to colon tumors in rodents, no anomalies were discerned in either rodent or non-rodent toxicity studies during the clinical review of Alirocumab and Evolocumab by the US FDA. This implies that any conceivable augmentation in bile acid levels is presumably clinically negligible. Conversely, both mAbs demonstrated the capability to inhibit the growth of subcutaneous MC38 colon cancer in syngeneic mouse models.^34^ Furthermore, while an established association exists between statin therapy and the risk of T2D,^494^ and certain mouse studies have suggested a requisite role of PCSK9 in maintaining pancreatic functionality,^495^ no established correlation has been found between PCSK9 inhibition and insulin resistance, alterations in blood glucose levels, pancreatic inadequacy, or elevated incidences of diabetes.^464,489,490,496^ However, considering the chronic and progressively developing nature of diabetes, it is imperative to conduct prolonged follow-up studies to meticulously evaluate the repercussions of PCSK9 inhibitors on pancreatic functionality and to elucidate their potential contributory role in the onset of diabetes.

In summary, the scrutiny of the PCSK9-iTs in clinical trials and subsequent studies has demonstrated them to be largely safe, with adverse effects being mild and uncommon, reaffirming their efficacy and safety profile in managing cholesterol levels. However, given the rapid advancement of PCSK9-iTs, it is imperative to meticulously monitor any long-term adverse reactions, particularly for novel treatments utilizing genetic depletion. Even minor potential off-target effects^389^ can result in irreversible modifications to the host. Furthermore, AAV vectors or LNPs, acting as foreign immunogens, have the potential to elicit significant immune responses within the host body.^497^ Hence, ongoing research and post-marketing surveillance are crucial to gather more extensive data on the long-term safety and uncommon AEs of PCSK9-iTs.

## Conclusions and final remarks

The discovery of PCSK9 in 2003 ignited a significant stride forward in our understanding of cholesterol regulation and its implications in a diverse spectrum of disorders. Over the ensuing two decades, we have witnessed substantial strides in unraveling the complexities of PCSK9’s biological features as well as its involvement in an array of physiological and pathological activities ranging from cholesterol homeostasis, cancer biology, to immunology, including but not limited to CVDs, liver diseases, infectious diseases, autoimmune disorders, neurocognitive disorders, and malignancies.^84,498^ PCSK9 operates as a navigator, shepherding specific surface protein receptors to degrade in cytosolic endosomes and/or lysosomes. These receptors include LDLR superfamily members including LDLR, VLDLR, ApoER2, as well as CD81, ACE2, and MHC-I. Interestingly, whereas PCSK9’s catalytic domain associates with specific protein receptors in the LDLR superfamily, the CHRD may play an important role in the interaction with additional receptors beyond the LDLR family, for example, the MHC-I. The rapid evolution from PCSK9’s discovery in 2003 to the approval of the first potent PCSK9-iT as mAb in 2015 owes much to a plethora of biological, genetic, epidemiological, and clinical studies conducted on mice and/or humans. Moreover, the successful development of PCSK9-iTs with robust safety profiles and superior inhibitory potency, such as the three mAbs and one siRNA, has made a significant contribution. A number of high-quality clinical studies, including the FOURIER, ODYSSEY Outcomes, ORION series, and CREDIT series have significantly deepened our knowledge of the multifaceted physiological functions of PCSK9. The remarkable transition from the laboratory discovery of PCSK9 to the safe clinical application of PCSK9-iTs is largely due to the narrow target range of PCSK9 and its primary expression in the liver. This exemplifies a successful bench-to-bedside journey in the field of biomedical research.^499^

Despite its integral role in cholesterol regulation, PCSK9 has been increasingly highlighted in cancer research over the last decade, especially following our discovery that PCSK9-iTs can augment immune checkpoint therapy for cancer treatment. Accumulating data have begun to uncover novel non-canonical roles of PCSK9 in carcinogenesis, metastasis, and antitumor immunity. However, some studies have reported contradictory observations of its role in certain cancer types. Furthermore, the multifaceted functions of PCSK9 in cancer development and the regulation of TIME have not been comprehensively addressed. For instance, further research is needed to explore PCSK9’s actions on cancer cells, its molecular signaling within TIME, and its roles in the modulation of antitumor immunity. It is therefore crucial to conduct more thorough investigations on its expression and primary function across different cancers before considering it as a potential antitumor therapeutic target. Moreover, research focusing on patient-derived xenografts (PDX) and/or tumor organoids in humanized mice and/or transgenic mice could greatly contribute to expanding our knowledge of the intricate roles PCSK9 plays within the TIME of human malignancies. This could further pave the way for launching more clinical trials to examine different strategies to inhibit PCSK9 in cancer patients.

Looking ahead to future research avenues, several aspects of PCSK9’s biology and its clinical applications need further study to deepen our overall understanding. For instance, there is a pressing need for more comprehensive research into PCSK9’s functions in organs other than the liver, such as the gastrointestinal tract, urinary system, immune system, and CNS. Additionally, exploring the potential functions of PCSK9 during developmental stages in the liver and other tissues, possibly independent of LDLR, is also worth examining further. We should also direct more in-depth examination towards specific PCSK9 domains and its interaction partners that connect with CD36 and other receptors outside of the LDLR family, with an aim to identify the elusive “protein X”. Moreover, there is a conspicuous absence of explicit evidence detailing the in vivo cytosolic pathway to degrade those protein targets of PCSK9, an ambiguity that requires further elucidation. From a clinical perspective, it is crucial to fully comprehend the enduring implications of PCSK9-iTs as well as the permanent PCSK9 suppression. Additionally, future research should explore how to decrease the cost of PCSK9-iTs, perhaps via oral administration resulted from novel strategies to develop emerging PCSK9 inhibitors, for example, MK-0616,^435^ to improve their worldwide accessibility. The potential influence of PCSK9-iTs on human cancer or TIME, either as standalone treatments or in combination with other therapies, is another research area to probe into. Similarly, their possible role in reducing the prevalence of certain infections, as well as autoimmune and neurocognitive disorders, also merits further exploration.

Therefore, future investigations may uncover additional unknown roles of PCSK9 that could be targeted by PCSK9-iTs, paving the way for new therapeutic applications of PCSK9-iTs in the treatment of diseases beyond their current uses.^500^ For example, the recent advancements in CRISPR technology, successfully applied to modify the *PCSK9* gene in nonhuman primates (NHPs) foreshadows a potential future where, under conditions of extreme pathological crisis, this advanced technology could be used to substitute Histidine152 with Glutamate152 in human hepatic PCSK9, a PCSK9 LOF variant recognized from the French Canadian variation.^20,21,123,396^ Such an alteration could render the zymogen unprocessable, retained in the ER, and provide enduring protection against the disorders associated with PCSK9 dysfunction. However, the transition of these applications from animal models to human pathology demands scrupulous validation. This is necessary to ensure the safe and successful implementation of these applications, and it should be done in line with the clinical trial models established during the development of the currently approved PCSK9-iTs.

In conclusion, the multifaceted biological functions of PCSK9 have been revealed in areas extending beyond cholesterol regulation. The therapeutic potential of targeting PCSK9 has been assessed in numerous preclinical studies, leading to well-structured clinical trials examining its efficacy in treating various disorders including malignancies. Future efforts should focus on developing safer, novel, and precision therapeutic strategies to target PCSK9, either independently or in conjunction with existing therapies for the treatment of CVDs, liver diseases, infections, autoimmune disorders, neurocognitive disorders, cancers, and etc.

## References

[1] Steiner DF, Cunningham D, Spigelman L, Aten B (1967). Insulin biosynthesis: evidence for a precursor. Science.

[2] Steiner DF (2011). On the discovery of precursor processing. Methods Mol. Biol..

[3] Chretien M, Li CH (1967). Isolation, purification, and characterization of gamma-lipotropic hormone from sheep pituitary glands. Can. J. Biochem.

[4] Chretien M (1976). Isolation of peptides with opiate activity from sheep and human pituitaries: relationship to beta-lipotropin. Biochem. Biophys. Res. Commun..

[5] Chretien M (2013). How the prohormone theory solved two important controversies in hormonal and neural Peptide biosynthesis. Front Endocrinol. (Lausanne).

[6] Seidah NG (1976). Letter: Fragment of sheep beta-lipotropin with morphine-like activity. Lancet.

[7] Seidah NG, Chretien M (1999). Proprotein and prohormone convertases: a family of subtilases generating diverse bioactive polypeptides. Brain Res..

[8] Cendron L (2023). Proprotein convertases regulate trafficking and maturation of key proteins within the secretory pathway. Adv. Protein Chem. Struct. Biol..

[9] Seidah NG, Prat A (2012). The biology and therapeutic targeting of the proprotein convertases. Nat. Rev. Drug Discov..

[10] Seidah NG (1999). Mammalian subtilisin/kexin isozyme SKI-1: A widely expressed proprotein convertase with a unique cleavage specificity and cellular localization. Proc. Natl Acad. Sci. USA.

[11] Seidah NG (2003). The secretory proprotein convertase neural apoptosis-regulated convertase 1 (NARC-1): liver regeneration and neuronal differentiation. Proc. Natl Acad. Sci. USA.

[12] Abifadel M (2003). Mutations in PCSK9 cause autosomal dominant hypercholesterolemia. Nat. Genet..

[13] Cunningham D (2007). Structural and biophysical studies of PCSK9 and its mutants linked to familial hypercholesterolemia. Nat. Struct. Mol. Biol..

[14] Piper DE (2007). The crystal structure of PCSK9: a regulator of plasma LDL-cholesterol. Structure.

[15] Naureckiene S (2003). Functional characterization of Narc 1, a novel proteinase related to proteinase K. Arch. Biochem. Biophys..

[16] Benjannet S (2004). NARC-1/PCSK9 and its natural mutants: zymogen cleavage and effects on the low density lipoprotein (LDL) receptor and LDL cholesterol. J. Biol. Chem..

[17] McNutt MC, Lagace TA, Horton JD (2007). Catalytic activity is not required for secreted PCSK9 to reduce low density lipoprotein receptors in HepG2 cells. J. Biol. Chem..

[18] Poirier S (2008). The proprotein convertase PCSK9 induces the degradation of low density lipoprotein receptor (LDLR) and its closest family members VLDLR and ApoER2. J. Biol. Chem..

[19] Benjannet S, Hamelin J, Chretien M, Seidah NG (2012). Loss- and gain-of-function PCSK9 variants: cleavage specificity, dominant negative effects, and low density lipoprotein receptor (LDLR) degradation. J. Biol. Chem..

[20] Mayne J (2011). Novel loss-of-function PCSK9 variant is associated with low plasma LDL cholesterol in a French-Canadian family and with impaired processing and secretion in cell culture. Clin. Chem..

[21] Lebeau PF (2021). The loss-of-function PCSK9Q152H variant increases ER chaperones GRP78 and GRP94 and protects against liver injury. J. Clin. Invest..

[22] Seidah NG (2019). The elusive inhibitory function of the acidic n-terminal segment of the prodomain of PCSK9: the plot thickens. J. Mol. Biol..

[23] Cohen JC, Boerwinkle E, Mosley TH, Hobbs HH (2006). Sequence variations in PCSK9, low LDL, and protection against coronary heart disease. N. Engl. J. Med..

[24] Berge KE, Ose L, Leren TP (2006). Missense mutations in the PCSK9 gene are associated with hypocholesterolemia and possibly increased response to statin therapy. Arterioscler Thromb. Vasc. Biol..

[25] Benjannet S (2006). The proprotein convertase (PC) PCSK9 is inactivated by furin and/or PC5/6A: functional consequences of natural mutations and post-translational modifications. J. Biol. Chem..

[26] Ben Djoudi Ouadda A (2019). Ser-phosphorylation of PCSK9 (proprotein convertase subtilisin-kexin 9) by Fam20C (family with sequence similarity 20, member C) kinase enhances its ability to degrade the LDLR (low-density lipoprotein receptor). Arterioscler Thromb. Vasc. Biol..

[27] Allard D (2005). Novel mutations of the PCSK9 gene cause variable phenotype of autosomal dominant hypercholesterolemia. Hum. Mutat..

[28] Cameron J (2006). Effect of mutations in the PCSK9 gene on the cell surface LDL receptors. Hum. Mol. Genet..

[29] Essalmani R (2011). In vivo evidence that furin from hepatocytes inactivates PCSK9. J. Biol. Chem..

[30] Timms KM (2004). A mutation in PCSK9 causing autosomal-dominant hypercholesterolemia in a Utah pedigree. Hum. Genet..

[31] Bottomley MJ (2009). Structural and biochemical characterization of the wild type PCSK9-EGF(AB) complex and natural familial hypercholesterolemia mutants. J. Biol. Chem..

[32] Hampton EN (2007). The self-inhibited structure of full-length PCSK9 at 1.9 A reveals structural homology with resistin within the C-terminal domain. Proc. Natl Acad. Sci. USA.

[33] Filkova M, Haluzik M, Gay S, Senolt L (2009). The role of resistin as a regulator of inflammation: implications for various human pathologies. Clin. Immunol..

[34] Liu X (2020). Inhibition of PCSK9 potentiates immune checkpoint therapy for cancer. Nature.

[35] Varret M (1999). A third major locus for autosomal dominant hypercholesterolemia maps to 1p34.1-p32. Am. J. Hum. Genet..

[36] Hunt SC (2000). Genetic localization to chromosome 1p32 of the third locus for familial hypercholesterolemia in a Utah kindred. Arterioscler Thromb. Vasc. Biol..

[37] Saavedra YG, Zhang J, Seidah NG (2013). PCSK9 prosegment chimera as novel inhibitors of LDLR degradation. PLoS ONE.

[38] Abifadel M (2012). Identification and characterization of new gain-of-function mutations in the PCSK9 gene responsible for autosomal dominant hypercholesterolemia. Atherosclerosis.

[39] Fagerberg L (2014). Analysis of the human tissue-specific expression by genome-wide integration of transcriptomics and antibody-based proteomics. Mol. Cell Proteom..

[40] Ferri N (2012). Proprotein convertase subtilisin kexin type 9 (PCSK9) secreted by cultured smooth muscle cells reduces macrophages LDLR levels. Atherosclerosis.

[41] Ding Z (2015). Cross-talk between LOX-1 and PCSK9 in vascular tissues. Cardiovascular Res..

[42] Ding Z (2015). Hemodynamic shear stress via ROS modulates PCSK9 expression in human vascular endothelial and smooth muscle cells and along the mouse aorta. Antioxid. Redox Signal.

[43] Tang ZH (2019). PCSK9: A novel inflammation modulator in atherosclerosis?. J. Cell Physiol..

[44] Ding Z (2016). Cross-talk between PCSK9 and damaged mtDNA in vascular smooth muscle cells: role in apoptosis. Antioxid. Redox Signal.

[45] Giunzioni I (2016). Local effects of human PCSK9 on the atherosclerotic lesion. J. Pathol..

[46] Wang F (2022). PCSK9 modulates macrophage polarization-mediated ventricular remodeling after myocardial infarction. J. Immunol. Res..

[47] Ding Z (2018). PCSK9 expression in the ischaemic heart and its relationship to infarct size, cardiac function, and development of autophagy. Cardiovasc Res..

[48] Ding Z (2022). Corrigendum to: PCSK9 expression in the ischaemic heart and its relationship to infarct size, cardiac function, and development of autophagy. Cardiovasc Res..

[49] Jeong HJ (2008). Sterol-dependent regulation of proprotein convertase subtilisin/kexin type 9 expression by sterol-regulatory element binding protein-2. J. Lipid Res..

[50] Li H (2009). Hepatocyte nuclear factor 1alpha plays a critical role in PCSK9 gene transcription and regulation by the natural hypocholesterolemic compound berberine. J. Biol. Chem..

[51] Tao R (2013). FoxO3 transcription factor and Sirt6 deacetylase regulate low density lipoprotein (LDL)-cholesterol homeostasis via control of the proprotein convertase subtilisin/kexin type 9 (Pcsk9) gene expression. J. Biol. Chem..

[52] Lin YK (2021). Pterostilbene increases LDL metabolism in HL-1 cardiomyocytes by modulating the PCSK9/HNF1alpha/SREBP2/LDLR signaling cascade, upregulating epigenetic hsa-miR-335 and hsa-miR-6825, and LDL receptor expression. Antioxid. (Basel).

[53] Dubuc G (2004). Statins upregulate PCSK9, the gene encoding the proprotein convertase neural apoptosis-regulated convertase-1 implicated in familial hypercholesterolemia. Arterioscler Thromb. Vasc. Biol..

[54] Costet P (2006). Hepatic PCSK9 expression is regulated by nutritional status via insulin and sterol regulatory element-binding protein 1c. J. Biol. Chem..

[55] Lebeau PF (2022). Caffeine blocks SREBP2-induced hepatic PCSK9 expression to enhance LDLR-mediated cholesterol clearance. Nat. Commun..

[56] Dong B, Singh AB, Shende VR, Liu J (2017). Hepatic HNF1 transcription factors control the induction of PCSK9 mediated by rosuvastatin in normolipidemic hamsters. Int J. Mol. Med..

[57] Li H, Liu J (2012). The novel function of HINFP as a co-activator in sterol-regulated transcription of PCSK9 in HepG2 cells. Biochem J..

[58] Ai D (2012). Regulation of hepatic LDL receptors by mTORC1 and PCSK9 in mice. J. Clin. Invest..

[59] Pan PW (2011). Structure and biochemical functions of SIRT6. J. Biol. Chem..

[60] Dong XC (2023). Sirtuin 6-A key regulator of hepatic lipid metabolism and liver health. Cells.

[61] Wang X (2020). A small-molecule inhibitor of PCSK9 transcription ameliorates atherosclerosis through the modulation of FoxO1/3 and HNF1alpha. EBioMedicine.

[62] Maxwell KN, Fisher EA, Breslow JL (2005). Overexpression of PCSK9 accelerates the degradation of the LDLR in a post-endoplasmic reticulum compartment. Proc. Natl Acad. Sci. USA.

[63] Maxwell KN, Breslow JL (2004). Adenoviral-mediated expression of Pcsk9 in mice results in a low-density lipoprotein receptor knockout phenotype. Proc. Natl Acad. Sci. USA.

[64] Park SW, Moon YA, Horton JD (2004). Post-transcriptional regulation of low density lipoprotein receptor protein by proprotein convertase subtilisin/kexin type 9a in mouse liver. J. Biol. Chem..

[65] Horton JD (2003). Combined analysis of oligonucleotide microarray data from transgenic and knockout mice identifies direct SREBP target genes. Proc. Natl Acad. Sci. USA.

[66] Attie AD, Seidah NG (2005). Dual regulation of the LDL receptor—some clarity and new questions. Cell Metab..

[67] Cohen J (2005). Low LDL cholesterol in individuals of African descent resulting from frequent nonsense mutations in PCSK9. Nat. Genet..

[68] Yang J (2001). Decreased lipid synthesis in livers of mice with disrupted Site-1 protease gene. Proc. Natl Acad. Sci. USA.

[69] Rashid S (2005). Decreased plasma cholesterol and hypersensitivity to statins in mice lacking Pcsk9. Proc. Natl Acad. Sci. USA.

[70] Zhao Z (2006). Molecular characterization of loss-of-function mutations in PCSK9 and identification of a compound heterozygote. Am. J. Hum. Genet..

[71] Hooper AJ, Marais AD, Tanyanyiwa DM, Burnett JR (2007). The C679X mutation in PCSK9 is present and lowers blood cholesterol in a Southern African population. Atherosclerosis.

[72] Luquero A, Badimon L, Borrell-Pages M (2021). PCSK9 functions in atherosclerosis are not limited to plasmatic ldl-cholesterol regulation. Front. Cardiovasc Med..

[73] Nassoury N (2007). The cellular trafficking of the secretory proprotein convertase PCSK9 and its dependence on the LDLR. Traffic.

[74] Qian YW (2007). Secreted PCSK9 downregulates low density lipoprotein receptor through receptor-mediated endocytosis. J. Lipid Res..

[75] Holla OL (2007). Degradation of the LDL receptors by PCSK9 is not mediated by a secreted protein acted upon by PCSK9 extracellularly. BMC Cell Biol..

[76] Lo Surdo P (2011). Mechanistic implications for LDL receptor degradation from the PCSK9/LDLR structure at neutral pH. EMBO Rep..

[77] Kwon HJ (2008). Molecular basis for LDL receptor recognition by PCSK9. Proc. Natl Acad. Sci. USA.

[78] Lagace TA (2006). Secreted PCSK9 decreases the number of LDL receptors in hepatocytes and in livers of parabiotic mice. J. Clin. Invest..

[79] Zhang DW (2007). Binding of proprotein convertase subtilisin/kexin type 9 to epidermal growth factor-like repeat A of low density lipoprotein receptor decreases receptor recycling and increases degradation. J. Biol. Chem..

[80] Horton JD, Cohen JC, Hobbs HH (2009). PCSK9: a convertase that coordinates LDL catabolism. J. Lipid Res..

[81] Zhang DW (2008). Structural requirements for PCSK9-mediated degradation of the low-density lipoprotein receptor. Proc. Natl Acad. Sci. USA.

[82] Holla OL (2011). Role of the C-terminal domain of PCSK9 in degradation of the LDL receptors. J. Lipid Res..

[83] Ni YG (2010). A proprotein convertase subtilisin-like/kexin type 9 (PCSK9) C-terminal domain antibody antigen-binding fragment inhibits PCSK9 internalization and restores low density lipoprotein uptake. J. Biol. Chem..

[84] Seidah NG, Prat A (2022). The multifaceted biology of PCSK9. Endocr. Rev..

[85] Jang HD (2020). Cyclase-associated protein 1 is a binding partner of proprotein convertase subtilisin/kexin type-9 and is required for the degradation of low-density lipoprotein receptors by proprotein convertase subtilisin/kexin type-9. Eur. Heart J..

[86] Fruchart Gaillard C (2023). Molecular interactions of PCSK9 with an inhibitory nanobody, CAP1 and HLA-C: Functional regulation of LDLR levels. Mol. Metab..

[87] Roubtsova A (2011). Circulating proprotein convertase subtilisin/kexin 9 (PCSK9) regulates VLDLR protein and triglyceride accumulation in visceral adipose tissue. Arterioscler Thromb. Vasc. Biol..

[88] Poirier S (2009). Dissection of the endogenous cellular pathways of PCSK9-induced low density lipoprotein receptor degradation: evidence for an intracellular route. J. Biol. Chem..

[89] Susan-Resiga D (2017). The proprotein convertase subtilisin/kexin type 9-resistant R410S low density lipoprotein receptor mutation: a novel mechanism causing familial hypercholesterolemia. J. Biol. Chem..

[90] Mikaeeli S (2020). Functional analysis of natural PCSK9 mutants in modern and archaic humans. FEBS J..

[91] Seidah NG (2017). The proprotein convertases in hypercholesterolemia and cardiovascular diseases: emphasis on proprotein convertase subtilisin/kexin 9. Pharm. Rev..

[92] Seidah NG, Awan Z, Chretien M, Mbikay M (2014). PCSK9: a key modulator of cardiovascular health. Circ. Res.

[93] Garcon D (2020). Circulating rather than intestinal PCSK9 (proprotein convertase subtilisin kexin type 9) regulates postprandial lipemia in mice. Arterioscler Thromb. Vasc. Biol..

[94] Peyot ML (2021). Substantial PCSK9 inactivation in beta-cells does not modify glucose homeostasis or insulin secretion in mice. Biochim. Biophys. Acta Mol. Cell Biol. Lipids.

[95] Ramin-Mangata S (2021). Effects of proprotein convertase subtilisin kexin type 9 modulation in human pancreatic beta cells function. Atherosclerosis.

[96] Alborn WE (2007). Serum proprotein convertase subtilisin kexin type 9 is correlated directly with serum LDL cholesterol. Clin. Chem..

[97] Mayne J (2007). Plasma PCSK9 levels correlate with cholesterol in men but not in women. Biochem. Biophys. Res Commun..

[98] Lambert G (2008). Plasma PCSK9 concentrations correlate with LDL and total cholesterol in diabetic patients and are decreased by fenofibrate treatment. Clin. Chem..

[99] Lakoski SG (2009). Genetic and metabolic determinants of plasma PCSK9 levels. J. Clin. Endocrinol. Metab..

[100] Dubuc G (2010). A new method for measurement of total plasma PCSK9: clinical applications. J. Lipid Res..

[101] Awan Z (2012). Rosuvastatin, proprotein convertase subtilisin/kexin type 9 concentrations, and LDL cholesterol response: the JUPITER trial. Clin. Chem..

[102] Jaworski K, Jankowski P, Kosior DA (2017). PCSK9 inhibitors—from discovery of a single mutation to a groundbreaking therapy of lipid disorders in one decade. Arch. Med Sci..

[103] Canuel M (2013). Proprotein convertase subtilisin/kexin type 9 (PCSK9) can mediate degradation of the low density lipoprotein receptor-related protein 1 (LRP-1). PLoS ONE.

[104] Lillis AP, Van Duyn LB, Murphy-Ullrich JE, Strickland DK (2008). LDL receptor-related protein 1: unique tissue-specific functions revealed by selective gene knockout studies. Physiol. Rev..

[105] Badimon L (2021). PCSK9 and LRP5 in macrophage lipid internalization and inflammation. Cardiovasc. Res..

[106] Desita SR (2022). PCSK9 and LRP6: potential combination targets to prevent and reduce atherosclerosis. J. Basic Clin. Physiol. Pharm..

[107] Shan L (2008). PCSK9 binds to multiple receptors and can be functionally inhibited by an EGF-A peptide. Biochem. Biophys. Res. Commun..

[108] Hey PJ (1998). Cloning of a novel member of the low-density lipoprotein receptor family. Gene.

[109] Demers A (2015). PCSK9 induces CD36 degradation and affects long-chain fatty acid uptake and triglyceride metabolism in adipocytes and in mouse liver. Arterioscler Thromb. Vasc. Biol..

[110] Adorni MP (2017). Inhibitory effect of PCSK9 on Abca1 protein expression and cholesterol efflux in macrophages. Atherosclerosis.

[111] Levy E (2013). PCSK9 plays a significant role in cholesterol homeostasis and lipid transport in intestinal epithelial cells. Atherosclerosis.

[112] Zhang X (2023). Immune regulation of the liver through the PCSK9/CD36 pathway during heart transplant rejection. Circulation.

[113] Le QT, Blanchet M, Seidah NG, Labonte P (2015). Plasma membrane tetraspanin CD81 complexes with proprotein convertase subtilisin/kexin type 9 (PCSK9) and low density lipoprotein receptor (LDLR), and its levels are reduced by PCSK9. J. Biol. Chem..

[114] Sharotri V (2012). Regulation of epithelial sodium channel trafficking by proprotein convertase subtilisin/kexin type 9 (PCSK9). J. Biol. Chem..

[115] Jonas MC, Costantini C, Puglielli L (2008). PCSK9 is required for the disposal of non-acetylated intermediates of the nascent membrane protein BACE1. EMBO Rep..

[116] Essalmani R (2023). SKI-1/S1P facilitates SARS-CoV-2 spike induced cell-to-cell fusion via activation of SREBP-2 and metalloproteases, whereas PCSK9 enhances the degradation of ACE2. Viruses.

[117] Brown MS, Goldstein JL (1976). Receptor-mediated control of cholesterol metabolism. Science.

[118] Guo Q, Feng X, Zhou Y (2020). PCSK9 variants in familial hypercholesterolemia: a comprehensive synopsis. Front Genet..

[119] Kotowski IK (2006). A spectrum of PCSK9 alleles contributes to plasma levels of low-density lipoprotein cholesterol. Am. J. Hum. Genet..

[120] Gai MT (2021). Polymorphisms of rs2483205 and rs562556 in the PCSK9 gene are associated with coronary artery disease and cardiovascular risk factors. Sci. Rep..

[121] Coggi D (2021). Relationship between circulating PCSK9 and markers of subclinical atherosclerosis—the improve study. Biomedicines.

[122] Zaid A (2008). Proprotein convertase subtilisin/kexin type 9 (PCSK9): hepatocyte-specific low-density lipoprotein receptor degradation and critical role in mouse liver regeneration. Hepatology.

[123] Chretien M, Mbikay M (2016). 60 YEARS OF POMC: From the prohormone theory to pro-opiomelanocortin and to proprotein convertases (PCSK1 to PCSK9). J. Mol. Endocrinol..

[124] Iacocca MA (2018). Whole-gene duplication of PCSK9 as a novel genetic mechanism for severe familial hypercholesterolemia. Can. J. Cardiol..

[125] Cariou B (2009). PCSK9 dominant negative mutant results in increased LDL catabolic rate and familial hypobetalipoproteinemia. Arterioscler Thromb. Vasc. Biol..

[126] Chapa JJ (2023). PCSK9 inhibition in patients after heart transplantation: a retrospective review and literature analysis. Curr. Heart Fail Rep..

[127] Jennings DL (2023). PCSK9 inhibitors safely and effectively lower LDL after heart transplantation: a systematic review and meta-analysis. Heart Fail Rev..

[128] Herbert B (2010). Increased secretion of lipoproteins in transgenic mice expressing human D374Y PCSK9 under physiological genetic control. Arterioscler. Thromb. Vasc. Biol..

[129] Bjorklund MM (2014). Induction of atherosclerosis in mice and hamsters without germline genetic engineering. Circ. Res..

[130] Bjorklund MM, Bernal JA, Bentzon JF (2022). Atherosclerosis induced by adeno-associated virus encoding gain-of-function PCSK9. Methods Mol. Biol..

[131] Al-Mashhadi RH (2013). Familial hypercholesterolemia and atherosclerosis in cloned minipigs created by DNA transposition of a human PCSK9 gain-of-function mutant. Sci. Transl. Med..

[132] Denis M (2012). Gene inactivation of proprotein convertase subtilisin/kexin type 9 reduces atherosclerosis in mice. Circulation.

[133] Wang H (2021). LincRNA-p21 alleviates atherosclerosis progression through regulating the miR-221/SIRT1/Pcsk9 axis. J. Cell Mol. Med..

[134] Ma CY (2021). Berberine attenuates atherosclerotic lesions and hepatic steatosis in ApoE(−/−) mice by down-regulating PCSK9 via ERK1/2 pathway. Ann. Transl. Med..

[135] Miranda MX (2015). The Sirt1 activator SRT3025 provides atheroprotection in Apoe−/− mice by reducing hepatic Pcsk9 secretion and enhancing Ldlr expression. Eur. Heart J..

[136] Huang YW (2022). 20(S)-protopanaxadiol decreases atherosclerosis in ApoE KO mice by increasing the levels of LDLR and inhibiting its binding with PCSK9. Food Funct..

[137] Camera M (2018). PCSK9 as a Positive Modulator of Platelet Activation. J. Am. Coll. Cardiol..

[138] Qi Z (2021). PCSK9 (proprotein convertase subtilisin/kexin 9) enhances platelet activation, thrombosis, and myocardial infarct expansion by binding to platelet CD36. Circulation.

[139] Barale C (2020). Platelet function and activation markers in primary hypercholesterolemia treated with anti-PCSK9 monoclonal antibody: A 12-month follow-up. Nutr. Metab. Cardiovasc. Dis..

[140] Cammisotto V (2021). Proprotein convertase subtilisin kexin type 9 inhibitors reduce platelet activation modulating ox-LDL pathways. Int J. Mol. Sci..

[141] Kotani K, Banach M (2017). Lipoprotein(a) and inhibitors of proprotein convertase subtilisin/kexin type 9. J. Thorac. Dis..

[142] Paciullo F (2022). Pleiotropic effects of PCSK9-inhibition on hemostasis: Anti-PCSK9 reduce FVIII levels by enhancing LRP1 expression. Thrombosis Res..

[143] Geovanini GR, Libby P (2018). Atherosclerosis and inflammation: overview and updates. Clin. Sci. (Lond.).

[144] Ma M, Hou C, Liu J (2023). Effect of PCSK9 on atherosclerotic cardiovascular diseases and its mechanisms: Focus on immune regulation. Front. Cardiovasc. Med..

[145] Fang C, Luo T, Lin L (2018). Elevation of serum proprotein convertase subtilisin/kexin type 9 (PCSK9) concentrations and its possible atherogenic role in patients with systemic lupus erythematosus. Ann. Transl. Med..

[146] Le Bras M (2013). Plasma PCSK9 is a late biomarker of severity in patients with severe trauma injury. J. Clin. Endocrinol. Metab..

[147] Li S (2015). Proprotein convertase subtilisin-kexin type 9 as a biomarker for the severity of coronary artery disease. Ann. Med..

[148] Li S (2014). Association of plasma PCSK9 levels with white blood cell count and its subsets in patients with stable coronary artery disease. Atherosclerosis.

[149] Zanni MV (2017). Proprotein convertase subtilisin/kexin 9 levels in relation to systemic immune activation and subclinical coronary plaque in HIV. Open Forum Infect. Dis..

[150] Zhang Y (2014). Relation of circulating PCSK9 concentration to fibrinogen in patients with stable coronary artery disease. J. Clin. Lipido..

[151] Polisecki E (2008). Genetic variation at the PCSK9 locus moderately lowers low-density lipoprotein cholesterol levels, but does not significantly lower vascular disease risk in an elderly population. Atherosclerosis.

[152] Zhang YX, Cliff WJ, Schoefl GI, Higgins G (1999). Coronary C-reactive protein distribution: its relation to development of atherosclerosis. Atherosclerosis.

[153] Ridker PM (2002). Comparison of C-reactive protein and low-density lipoprotein cholesterol levels in the prediction of first cardiovascular events. N. Engl. J. Med.

[154] Feingold KR (2008). Inflammation stimulates the expression of PCSK9. Biochem. Biophys. Res. Commun..

[155] Xu Q (2023). PCSK9: A emerging participant in heart failure. Biomed. Pharmacother..

[156] Huang G (2022). PCSK9 inhibition protects against myocardial ischemia-reperfusion injury via suppressing autophagy. Microvasc. Res..

[157] Krychtiuk KA (2021). Circulating levels of proprotein convertase subtilisin/kexin type 9 (PCSK9) are associated with monocyte subsets in patients with stable coronary artery disease. J. Clin. Lipido..

[158] Wang X (2020). PCSK9 regulates pyroptosis via mtDNA damage in chronic myocardial ischemia. Basic Res. Cardiol..

[159] Cheng JM (2016). PCSK9 in relation to coronary plaque inflammation: results of the ATHEROREMO-IVUS study. Atherosclerosis.

[160] Tang ZH (2017). New role of PCSK9 in atherosclerotic inflammation promotion involving the TLR4/NF-kappaB pathway. Atherosclerosis.

[161] Tang Z (2012). PCSK9 siRNA suppresses the inflammatory response induced by oxLDL through inhibition of NF-kappaB activation in THP-1-derived macrophages. Int J. Mol. Med..

[162] Ricci C (2018). PCSK9 induces a pro-inflammatory response in macrophages. Sci. Rep..

[163] Kuhnast S (2014). Alirocumab inhibits atherosclerosis, improves the plaque morphology, and enhances the effects of a statin. J. Lipid Res..

[164] Landlinger C (2017). The AT04A vaccine against proprotein convertase subtilisin/kexin type 9 reduces total cholesterol, vascular inflammation, and atherosclerosis in APOE*3Leiden.CETP mice. Eur. Heart J..

[165] Barcena ML (2023). The impact of the PCSK-9/VLDL-receptor axis on inflammatory cell polarization. Cytokine.

[166] Rehues P (2023). PCSK9 inhibitors have apolipoprotein C-III-related anti-inflammatory activity, assessed by 1H-NMR glycoprotein profile in subjects at high or very high cardiovascular risk. Int J. Mol. Sci..

[167] Bernelot Moens SJ (2017). PCSK9 monoclonal antibodies reverse the pro-inflammatory profile of monocytes in familial hypercholesterolaemia. Eur. Heart J..

[168] Grune J (2017). PCSK9 regulates the chemokine receptor CCR2 on monocytes. Biochem. Biophys. Res. Commun..

[169] Nahrendorf M, Swirski FK (2017). Cholesterol, CCR2, and monocyte phenotypes in atherosclerosis. Eur. Heart J..

[170] Liu A, Frostegard J (2018). PCSK9 plays a novel immunological role in oxidized LDL-induced dendritic cell maturation and activation of T cells from human blood and atherosclerotic plaque. J. Intern. Med..

[171] Sundararaman SS, Doring Y, van der Vorst EPC (2021). PCSK9: a multi-faceted protein that is involved in cardiovascular biology. Biomedicines.

[172] Ragusa R (2021). PCSK9 and atherosclerosis: looking beyond LDL regulation. Eur. J. Clin. Invest.

[173] Tang Y (2020). Research progress on alternative non-classical mechanisms of PCSK9 in atherosclerosis in patients with and without diabetes. Cardiovasc Diabetol..

[174] Xie W (2016). Association between plasma PCSK9 levels and 10-year progression of carotid atherosclerosis beyond LDL-C: A cohort study. Int J. Cardiol..

[175] Zhu YM (2015). Association of proprotein convertase subtilisin/kexin type 9 (PCSK9) with cardiovascular risk in primary prevention. Arterioscler Thromb. Vasc. Biol..

[176] Nicholls SJ (2018). Effect of evolocumab on coronary plaque composition. J. Am. Coll. Cardiol..

[177] Cyr Y (2021). Lower plasma PCSK9 in normocholesterolemic subjects is associated with upregulated adipose tissue surface-expression of LDLR and CD36 and NLRP3 inflammasome. Physiol. Rep..

[178] Lee JS, O’Connell EM, Pacher P, Lohoff FW (2021). PCSK9 and the gut-liver-brain axis: a novel therapeutic target for immune regulation in alcohol use disorder. J. Clin. Med..

[179] Grefhorst A, McNutt MC, Lagace TA, Horton JD (2008). Plasma PCSK9 preferentially reduces liver LDL receptors in mice. J. Lipid Res..

[180] Paquette M (2020). Circulating PCSK9 is associated with liver biomarkers and hepatic steatosis. Clin. Biochem..

[181] Taskinen MR (2020). Impact of proprotein convertase subtilisin/kexin type 9 inhibition with evolocumab on the postprandial responses of triglyceride-rich lipoproteins in type II diabetic subjects. J. Clin. Lipido..

[182] Tavori H, Rashid S, Fazio S (2015). On the function and homeostasis of PCSK9: reciprocal interaction with LDLR and additional lipid effects. Atherosclerosis.

[183] Momtazi-Borojeni AA, Banach M, Ruscica M, Sahebkar A (2022). The role of PCSK9 in NAFLD/NASH and therapeutic implications of PCSK9 inhibition. Expert Rev. Clin. Pharm..

[184] Lebeau PF (2019). Diet-induced hepatic steatosis abrogates cell-surface LDLR by inducing de novo PCSK9 expression in mice. J. Biol. Chem..

[185] Lebeau PF (2019). Pcsk9 knockout exacerbates diet-induced non-alcoholic steatohepatitis, fibrosis and liver injury in mice. JHEP Rep..

[186] Ioannou GN (2022). Pcsk9 deletion promotes murine nonalcoholic steatohepatitis and hepatic carcinogenesis: role of cholesterol. Hepatol. Commun..

[187] Shapiro MD, Miles J, Tavori H, Fazio S (2018). Diagnosing resistance to a proprotein convertase subtilisin/kexin type 9 inhibitor. Ann. Intern Med..

[188] Lee JS (2019). PCSK9 inhibition as a novel therapeutic target for alcoholic liver disease. Sci. Rep..

[189] Grimaudo S (2021). PCSK9 rs11591147 R46L loss-of-function variant protects against liver damage in individuals with NAFLD. Liver Int..

[190] Lai Q (2017). E2F1 inhibits circulating cholesterol clearance by regulating Pcsk9 expression in the liver. JCI Insight.

[191] Emma MR (2020). Hepatic and circulating levels of PCSK9 in morbidly obese patients: Relation with severity of liver steatosis. Biochim Biophys. Acta Mol. Cell Biol. Lipids.

[192] Ruscica M (2016). Liver fat accumulation is associated with circulating PCSK9. Ann. Med..

[193] Wargny M (2018). Circulating PCSK9 levels are not associated with the severity of hepatic steatosis and NASH in a high-risk population. Atherosclerosis.

[194] Baragetti A (2017). PCSK9 deficiency results in increased ectopic fat accumulation in experimental models and in humans. Eur. J. Prev. Cardiol..

[195] Scicali R (2021). Analysis of steatosis biomarkers and inflammatory profile after adding on PCSK9 inhibitor treatment in familial hypercholesterolemia subjects with nonalcoholic fatty liver disease: a single lipid center real-world experience. Nutr. Metab. Cardiovasc. Dis..

[196] Shafiq M (2020). Effects of proprotein convertase subtilisin/kexin type-9 inhibitors on fatty liver. World J. Hepatol..

[197] Sekhon AK (2021). A New Potential Strategy for Acute Non-Alcoholic Steatohepatitis (NASH). Am. J. Case Rep..

[198] Molina S (2007). The low-density lipoprotein receptor plays a role in the infection of primary human hepatocytes by hepatitis C virus. J. Hepatol..

[199] Felmlee DJ (2013). Hepatitis C virus, cholesterol and lipoproteins—impact for the viral life cycle and pathogenesis of liver disease. Viruses.

[200] Caron J (2019). Low-density lipoprotein receptor-deficient hepatocytes differentiated from induced pluripotent stem cells allow familial hypercholesterolemia modeling, CRISPR/Cas-mediated genetic correction, and productive hepatitis C virus infection. Stem Cell Res. Ther..

[201] D’Ambrosio R (2019). Real-world effectiveness and safety of glecaprevir/pibrentasvir in 723 patients with chronic hepatitis C. J. Hepatol..

[202] Hyrina A (2017). Treatment-induced viral cure of hepatitis C virus-infected patients involves a dynamic interplay among three important molecular players in lipid homeostasis: circulating microRNA (miR)-24, miR-223, and proprotein convertase subtilisin/kexin type 9. EBioMedicine.

[203] Bridge SH (2015). PCSK9, apolipoprotein E and lipoviral particles in chronic hepatitis C genotype 3: evidence for genotype-specific regulation of lipoprotein metabolism. J. Hepatol..

[204] Labonte P (2009). PCSK9 impedes hepatitis C virus infection in vitro and modulates liver CD81 expression. Hepatology.

[205] Seidah NG (2016). New developments in proprotein convertase subtilisin-kexin 9’s biology and clinical implications. Curr. Opin. Lipido..

[206] Pirro M (2017). Hepatitis C virus and proprotein convertase subtilisin/kexin type 9: a detrimental interaction to increase viral infectivity and disrupt lipid metabolism. J. Cell Mol. Med..

[207] Seidah NG, Pasquato A, Andreo U (2021). How do enveloped viruses exploit the secretory proprotein convertases to regulate infectivity and spread?. Viruses.

[208] Magnasco L (2022). The role of PCSK9 in infectious diseases. Curr. Med Chem..

[209] Leucker TM (2018). Coronary endothelial dysfunction is associated with elevated serum PCSK9 levels in people with HIV independent of low-density lipoprotein cholesterol. J. Am. Heart Assoc..

[210] Leucker TM (2020). Evolocumab, a PCSK9-monoclonal antibody, rapidly reverses coronary artery endothelial dysfunction in people living with HIV and People with Dyslipidemia. J. Am. Heart Assoc..

[211] Bhatt S (2013). The global distribution and burden of dengue. Nature.

[212] Gan ES (2020). Dengue virus induces PCSK9 expression to alter antiviral responses and disease outcomes. J. Clin. Invest..

[213] Brown MS, Goldstein JL (1997). The SREBP pathway: regulation of cholesterol metabolism by proteolysis of a membrane-bound transcription factor. Cell.

[214] Li Z, Liu Q (2018). Proprotein convertase subtilisin/kexin type 9 inhibits interferon beta expression through interacting with ATF-2. FEBS Lett..

[215] Tikoo K (2015). Tissue specific up regulation of ACE2 in rabbit model of atherosclerosis by atorvastatin: role of epigenetic histone modifications. Biochem. Pharm..

[216] Shin YH (2017). The effect of fluvastatin on cardiac fibrosis and angiotensin-converting enzyme-2 expression in glucose-controlled diabetic rat hearts. Heart Vessels.

[217] Zhang XJ (2020). In-hospital use of statins is associated with a reduced risk of mortality among individuals with COVID-19. Cell Metab..

[218] Tan WYT (2020). Statin use is associated with lower disease severity in COVID-19 infection. Sci. Rep..

[219] Memel, Z. N. et al. Statins are associated with improved 28-day mortality in patients hospitalized with SARS-CoV-2 infection. *medRxiv*10.1101/2021.03.27.21254373 (2021).

[220] Vuorio A, Kovanen PT (2020). Prevention of endothelial dysfunction and thrombotic events in COVID-19 patients with familial hypercholesterolemia. J. Clin. Lipido..

[221] Vuorio A, Kovanen PT (2021). PCSK9 inhibitors for COVID-19: an opportunity to enhance the antiviral action of interferon in patients with hypercholesterolaemia. J. Intern. Med..

[222] Zhang Q (2020). Inborn errors of type I IFN immunity in patients with life-threatening COVID-19. Science.

[223] Navarese EP (2023). PCSK9 inhibition during the inflammatory stage of SARS-CoV-2 infection. J. Am. Coll. Cardiol..

[224] Goonewardena SN, Rosenson RS (2023). PCSK9: the nexus of lipoprotein metabolism and inflammation in COVID-19. J. Am. Coll. Cardiol..

[225] Singer M (2016). The third international consensus definitions for sepsis and septic shock (Sepsis-3). JAMA.

[226] Russell JA (2006). Management of sepsis. N. Engl. J. Med..

[227] Walley KR (2014). PCSK9 is a critical regulator of the innate immune response and septic shock outcome. Sci. Transl. Med..

[228] Boyd JH (2016). Increased plasma PCSK9 levels are associated with reduced endotoxin clearance and the development of acute organ failures during sepsis. J. Innate Immun..

[229] Walley KR (2016). Role of lipoproteins and proprotein convertase subtilisin/kexin type 9 in endotoxin clearance in sepsis. Curr. Opin. Crit. Care.

[230] Dwivedi DJ (2016). Differential expression of PCSK9 modulates infection, inflammation, and coagulation in a murine model of sepsis. Shock.

[231] Grin PM (2018). Low-density lipoprotein (LDL)-dependent uptake of Gram-positive lipoteichoic acid and Gram-negative lipopolysaccharide occurs through LDL receptor. Sci. Rep..

[232] Yuan Y (2020). PCSK9: a potential therapeutic target for sepsis. J. Immunol. Res..

[233] Leung AKK (2019). Reduced proprotein convertase subtilisin/kexin 9 (PCSK9) function increases lipoteichoic acid clearance and improves outcomes in Gram positive septic shock patients. Sci. Rep..

[234] Feng Q (2019). A genetic approach to the association between PCSK9 and sepsis. JAMA Netw. Open.

[235] Genga KR (2018). Impact of PCSK9 loss-of-function genotype on 1-year mortality and recurrent infection in sepsis survivors. EBioMedicine.

[236] Shimada T (2020). Very low density lipoprotein receptor sequesters lipopolysaccharide into adipose tissue during sepsis. Crit. Care Med..

[237] Efron PA, Mohr AM, Moore FA, Moldawer LL (2015). The future of murine sepsis and trauma research models. J. Leukoc. Biol..

[238] Shaler CR (2017). MAIT cells launch a rapid, robust and distinct hyperinflammatory response to bacterial superantigens and quickly acquire an anergic phenotype that impedes their cognate antimicrobial function: Defining a novel mechanism of superantigen-induced immunopathology and immunosuppression. PLoS Biol..

[239] Ernst W (2013). Humanized mice, a new model to study the influence of drug treatment on neonatal sepsis. Infect. Immun..

[240] Atreya MR (2020). Proprotein convertase subtilisin/kexin type 9 loss-of-function is detrimental to the juvenile host with septic shock. Crit. Care Med..

[241] Vecchie A (2021). PCSK9 is associated with mortality in patients with septic shock: data from the ALBIOS study. J. Intern. Med..

[242] Innocenti F (2021). Plasma PCSK9 levels and sepsis severity: an early assessment in the emergency department. Clin. Exp. Med..

[243] Rannikko J (2019). Reduced plasma PCSK9 response in patients with bacteraemia is associated with mortality. J. Intern. Med..

[244] Trinder M, Boyd JH, Brunham LR (2019). Molecular regulation of plasma lipid levels during systemic inflammation and sepsis. Curr. Opin. Lipido..

[245] Zhou Z (2023). The association between PCSK9 inhibitor use and sepsis: a systematic review and meta-analysis of 20 double-blind, randomized, placebo-controlled trials. Am. J. Med..

[246] Mbikay M, Mayne J, Seidah NG, Chretien M (2007). Of PCSK9, cholesterol homeostasis and parasitic infections: possible survival benefits of loss-of-function PCSK9 genetic polymorphisms. Med. Hypotheses.

[247] Arama C (2018). Malaria severity: possible influence of the E670G PCSK9 polymorphism: a preliminary case-control study in Malian children. PLoS ONE.

[248] Fedoryak O (2020). Association of the rs562556 PCSK9 gene polymorphism with reduced mortality in severe malaria among malian children. Can. J. Infect. Dis. Med Microbiol..

[249] Farley E, Menter A (2011). Psoriasis: comorbidities and associations. G Ital. Dermatol Venereol..

[250] Fernandez-Armenteros JM (2019). Psoriasis, metabolic syndrome and cardiovascular risk factors. A population-based study. J. Eur. Acad. Dermatol. Venereol..

[251] Fiore M (2018). Liver illness and psoriatic patients. Biomed. Res. Int..

[252] Masson W, Lobo M, Molinero G (2020). Psoriasis and cardiovascular risk: a comprehensive review. Adv. Ther..

[253] Santos-Juanes J (2015). Psoriasis vulgaris with or without arthritis and independent of disease severity or duration is a risk factor for hypercholesterolemia. Dermatology.

[254] Orem A (1999). The significance of autoantibodies against oxidatively modified low-density lipoprotein (LDL) in patients with psoriasis. Clin. Chim. Acta.

[255] Meijer K (2011). Human primary adipocytes exhibit immune cell function: adipocytes prime inflammation independent of macrophages. PLoS ONE.

[256] Socha M (2020). The effect of statins on psoriasis severity: a meta-analysis of randomized clinical trials. Arch. Med. Sci..

[257] Merleev A (2022). Proprotein convertase subtilisin/kexin type 9 is a psoriasis-susceptibility locus that is negatively related to IL36G. JCI Insight.

[258] Luan C (2019). Potentiation of psoriasis-like inflammation by PCSK9. J. Invest. Dermatol..

[259] Cao A, Wu M, Li H, Liu J (2011). Janus kinase activation by cytokine oncostatin M decreases PCSK9 expression in liver cells. J. Lipid Res..

[260] Krahel JA (2020). Methotrexate decreases the level of PCSK9-a novel indicator of the risk of proatherogenic lipid profile in psoriasis. the preliminary data. J. Clin. Med.

[261] Garshick MS (2021). Characterization of PCSK9 in the blood and skin of psoriasis. J. Invest. Dermatol..

[262] Zhao SS, Yiu ZZN, Barton A, Bowes J (2023). Association of lipid-lowering drugs with risk of psoriasis: a mendelian randomization study. JAMA Dermatol..

[263] Cross M (2014). The global burden of rheumatoid arthritis: estimates from the global burden of disease 2010 study. Ann. Rheum. Dis..

[264] Moreland LW (1997). Treatment of rheumatoid arthritis with a recombinant human tumor necrosis factor receptor (p75)-Fc fusion protein. N. Engl. J. Med..

[265] Wijbrandts CA, Tak PP (2017). Prediction of response to targeted treatment in rheumatoid arthritis. Mayo Clin. Proc..

[266] van Vollenhoven RF, Nagy G, Tak PP (2014). Early start and stop of biologics: has the time come?. BMC Med..

[267] Arida A (2021). PCSK9/LDLR system and rheumatoid arthritis-related atherosclerosis. Front. Cardiovasc. Med..

[268] Frostegard J (2011). Cardiovascular co-morbidity in patients with rheumatic diseases. Arthritis Res. Ther..

[269] Choy E (2014). Cardiovascular risk in rheumatoid arthritis: recent advances in the understanding of the pivotal role of inflammation, risk predictors and the impact of treatment. Rheumatology (Oxford).

[270] Karpouzas GA, Ormseth SR, Hernandez E, Budoff MJ (2020). Biologics may prevent cardiovascular events in rheumatoid arthritis by inhibiting coronary plaque formation and stabilizing high-risk lesions. Arthritis Rheumatol..

[271] Frostegard J (2021). Low levels of PCSK9 are associated with remission in patients with rheumatoid arthritis treated with anti-TNF-alpha: potential underlying mechanisms. Arthritis Res. Ther..

[272] Meng Y (2023). Circulating PCSK9 relates to aggravated disease activity, Th17/Treg imbalance, and predicts treatment outcome of conventional synthetic DMARDs in rheumatoid arthritis patients. Ir. J. Med. Sci..

[273] Alam J, Jantan I, Bukhari SNA (2017). Rheumatoid arthritis: recent advances on its etiology, role of cytokines and pharmacotherapy. Biomed. Pharmacother..

[274] Udalova IA, Mantovani A, Feldmann M (2016). Macrophage heterogeneity in the context of rheumatoid arthritis. Nat. Rev. Rheumatol..

[275] Frostegard J (2022). The role of PCSK9 in inflammation, immunity, and autoimmune diseases. Expert Rev. Clin. Immunol..

[276] Frostegård J (2021). Low levels of PCSK9 are associated with remission in patients with rheumatoid arthritis treated with anti-TNF-α: potential underlying mechanisms. Arthritis Res. Ther..

[277] Koch AE (1992). Enhanced production of monocyte chemoattractant protein-1 in rheumatoid arthritis. J. Clin. Invest..

[278] Shahrara S (2008). Inhibition of monocyte chemoattractant protein-1 ameliorates rat adjuvant-induced arthritis. J. Immunol..

[279] Winyard PG (1993). Presence of foam cells containing oxidised low density lipoprotein in the synovial membrane from patients with rheumatoid arthritis. Ann. Rheum. Dis..

[280] Wada Y (2005). Autoantibodies against oxidized low-density lipoprotein (LDL) and carotid atherosclerosis in patients with rheumatoid arthritis. Clin. Exp. Rheumatol..

[281] Nowak B (2016). Disease activity, oxidized-LDL fraction and anti-oxidized LDL antibodies influence cardiovascular risk in rheumatoid arthritis. Adv. Clin. Exp. Med..

[282] Scheinecker C, Goschl L, Bonelli M (2020). Treg cells in health and autoimmune diseases: New insights from single cell analysis. J. Autoimmun..

[283] Urowitz MB (1976). The bimodal mortality pattern of systemic lupus erythematosus. Am. J. Med..

[284] Skaggs BJ, Hahn BH, McMahon M (2012). Accelerated atherosclerosis in patients with SLE—mechanisms and management. Nat. Rev. Rheumatol..

[285] Frieri M, Stampfl H (2016). Systemic lupus erythematosus and atherosclerosis: review of the literature. Autoimmun. Rev..

[286] Schanberg LE (2012). Use of atorvastatin in systemic lupus erythematosus in children and adolescents. Arthritis Rheum..

[287] van Leuven SI (2012). Mycophenolate mofetil but not atorvastatin attenuates atherosclerosis in lupus-prone LDLr(-/-) mice. Ann. Rheum. Dis..

[288] Liu A (2020). Proprotein convertase subtilisin kexin 9 is associated with disease activity and is implicated in immune activation in systemic lupus erythematosus. Lupus.

[289] Mok CC (2023). Circulating proprotein convertase subtilisin/kexin type 9 (PCSK9) is associated with disease activity and risk of incident cardiovascular disease in patients with systemic lupus erythematosus. Inflammation.

[290] Cai J (2022). Serum PCSK9 is positively correlated with disease activity and Th17 cells, while its short-term decline during treatment reflects desirable outcomes in ankylosing spondylitis patients. Ir. J. Med. Sci..

[291] Tiniakou E, Rivera E, Mammen AL, Christopher-Stine L (2019). Use of proprotein convertase subtilisin/kexin type 9 inhibitors in statin-associated immune-mediated necrotizing myopathy: a case series. Arthritis Rheumatol..

[292] de Dios Garcia-Diaz J (2019). Proprotein convertase subtilisin/kexin type 9 antibody and statin-associated autoimmune myopathy. Ann. Intern. Med..

[293] Vigne S (2022). Lowering blood cholesterol does not affect neuroinflammation in experimental autoimmune encephalomyelitis. J. Neuroinflammation.

[294] Bell AS, Wagner J, Rosoff DB, Lohoff FW (2023). Proprotein convertase subtilisin/kexin type 9 (PCSK9) in the central nervous system. Neurosci. Biobehav. Rev..

[295] Vitali C, Wellington CL, Calabresi L (2014). HDL and cholesterol handling in the brain. Cardiovasc. Res..

[296] Dietschy JM (2009). Central nervous system: cholesterol turnover, brain development and neurodegeneration. Biol. Chem..

[297] Nieweg K, Schaller H, Pfrieger FW (2009). Marked differences in cholesterol synthesis between neurons and glial cells from postnatal rats. J. Neurochem..

[298] Chen YQ, Troutt JS, Konrad RJ (2014). PCSK9 is present in human cerebrospinal fluid and is maintained at remarkably constant concentrations throughout the course of the day. Lipids.

[299] Lee JS (2019). PCSK9 is increased in cerebrospinal fluid of individuals with alcohol use disorder. Alcohol Clin. Exp. Res..

[300] Zimetti F (2017). Increased PCSK9 cerebrospinal fluid concentrations in Alzheimer’s disease. J. Alzheimers Dis..

[301] Bjorkhem I, Meaney S (2004). Brain cholesterol: long secret life behind a barrier. Arterioscler Thromb. Vasc. Biol..

[302] Tracey TJ, Steyn FJ, Wolvetang EJ, Ngo ST (2018). Neuronal lipid metabolism: multiple pathways driving functional outcomes in health and disease. Front. Mol. Neurosci..

[303] Wu Q (2014). The dual behavior of PCSK9 in the regulation of apoptosis is crucial in Alzheimer’s disease progression (Review). Biomed. Rep..

[304] Wang L (2018). Inhibition of proprotein convertase subtilisin/kexin type 9 attenuates neuronal apoptosis following focal cerebral ischemia via apolipoprotein E receptor 2 downregulation in hyperlipidemic mice. Int. J. Mol. Med..

[305] Abuelezz SA, Hendawy N (2021). HMGB1/RAGE/TLR4 axis and glutamate as novel targets for PCSK9 inhibitor in high fat cholesterol diet induced cognitive impairment and amyloidosis. Life Sci..

[306] Adorni MP (2019). Proprotein convertase subtilisin/kexin type 9, brain cholesterol homeostasis and potential implication for Alzheimer’s disease. Front. Aging Neurosci..

[307] Mazura AD (2022). PCSK9 acts as a key regulator of Abeta clearance across the blood-brain barrier. Cell Mol. Life Sci..

[308] Hendawy N, Salaheldin TH, Abuelezz SA (2023). PCSK9 inhibition reduces depressive like behavior in CUMS-exposed rats: highlights on HMGB1/RAGE/TLR4 pathway, NLRP3 inflammasome complex and IDO-1. J. Neuroimmune Pharm..

[309] Courtemanche H (2018). PCSK9 concentrations in cerebrospinal fluid are not specifically increased in Alzheimer’s disease. J. Alzheimers Dis..

[310] Apaijai N (2019). Pretreatment with PCSK9 inhibitor protects the brain against cardiac ischemia/reperfusion injury through a reduction of neuronal inflammation and amyloid beta aggregation. J. Am. Heart Assoc..

[311] Liu M (2010). PCSK9 is not involved in the degradation of LDL receptors and BACE1 in the adult mouse brain. J. Lipid Res..

[312] Fu T, Guan Y, Xu J, Wang Y (2017). APP, APLP2 and LRP1 interact with PCSK9 but are not required for PCSK9-mediated degradation of the LDLR in vivo. Biochim. Biophys. Acta Mol. Cell Biol. Lipids.

[313] Picard C (2019). Proprotein convertase subtilisin/kexin type 9 (PCSK9) in Alzheimer’s disease: a genetic and proteomic multi-cohort study. PLoS ONE.

[314] Shibata N (2005). No genetic association between PCSK9 polymorphisms and Alzheimer’s disease and plasma cholesterol level in Japanese patients. Psychiatr. Genet..

[315] Reynolds CA (2010). Analysis of lipid pathway genes indicates association of sequence variation near SREBF1/TOM1L2/ATPAF2 with dementia risk. Hum. Mol. Genet..

[316] Paquette M (2018). Loss-of-function PCSK9 mutations are not associated with Alzheimer disease. J. Geriatr. Psychiatry Neurol..

[317] Benn M, Nordestgaard BG, Frikke-Schmidt R, Tybjaerg-Hansen A (2017). Low LDL cholesterol, PCSK9 and HMGCR genetic variation, and risk of Alzheimer’s disease and Parkinson’s disease: Mendelian randomisation study. BMJ.

[318] You M, Fischer M, Deeg MA, Crabb DW (2002). Ethanol induces fatty acid synthesis pathways by activation of sterol regulatory element-binding protein (SREBP). J. Biol. Chem..

[319] Rosoff DB (2019). Association of high-intensity binge drinking with lipid and liver function enzyme levels. JAMA Netw. Open.

[320] Rosoff DB (2020). Lipid profile dysregulation predicts alcohol withdrawal symptom severity in individuals with alcohol use disorder. Alcohol.

[321] Habib A (2005). High-density lipoprotein cholesterol as an indicator of liver function and prognosis in noncholestatic cirrhotics. Clin. Gastroenterol. Hepatol..

[322] Bhattacharya A, Chowdhury A, Chaudhury K, Shukla PC (2021). Proprotein convertase subtilisin/kexin type 9 (PCSK9): a potential multifaceted player in cancer. Biochim. Biophys. Acta Rev. Cancer.

[323] Mahboobnia K (2021). PCSK9 and cancer: rethinking the link. Biomed. Pharmacother..

[324] King RJ, Singh PK, Mehla K (2022). The cholesterol pathway: impact on immunity and cancer. Trends Immunol..

[325] Cerami E (2012). The cBio cancer genomics portal: an open platform for exploring multidimensional cancer genomics data. Cancer Discov..

[326] Gao J (2013). Integrative analysis of complex cancer genomics and clinical profiles using the cBioPortal. Sci. Signal.

[327] Xu B (2020). Proprotein convertase subtilisin/kexin type 9 promotes gastric cancer metastasis and suppresses apoptosis by facilitating MAPK signaling pathway through HSP70 up-regulation. Front. Oncol..

[328] Zhou B (2020). Plasma proteomics-based identification of novel biomarkers in early gastric cancer. Clin. Biochem..

[329] Fasolato S (2020). PCSK9 levels are raised in chronic HCV patients with hepatocellular carcinoma. J. Clin. Med..

[330] Bhat M (2015). Decreased PCSK9 expression in human hepatocellular carcinoma. BMC Gastroenterol..

[331] Pitteri SJ (2011). Tumor microenvironment-derived proteins dominate the plasma proteome response during breast cancer induction and progression. Cancer Res..

[332] Ozkan C (2015). Proprotein convertase subtilisin/kexin type 9 (PCSK9), soluble lectin-like oxidized LDL receptor 1 (sLOX-1) and ankle brachial index in patients with differentiated thyroid cancer. Endocr. J..

[333] Wong Chong E (2022). Circulating levels of PCSK9, ANGPTL3 and Lp(a) in stage III breast cancers. BMC Cancer.

[334] Luo X (2022). Bioinformatics identification of key genes for the development and prognosis of lung adenocarcinoma. Inquiry.

[335] Li L, Lu S, Ma C (2022). Anti-proliferative and pro-apoptotic effects of curcumin on skin cutaneous melanoma: bioinformatics analysis and in vitro experimental studies. Front. Genet..

[336] Zhang SZ (2021). PCSK9 promotes tumor growth by inhibiting tumor cell apoptosis in hepatocellular carcinoma. Exp. Hematol. Oncol..

[337] Ito M (2021). Association of serum anti-PCSK9 antibody levels with favorable postoperative prognosis in esophageal cancer. Front. Oncol..

[338] Sun Y (2022). S-palmitoylation of PCSK9 induces sorafenib resistance in liver cancer by activating the PI3K/AKT pathway. Cell Rep..

[339] Sung H (2021). Global Cancer Statistics 2020: GLOBOCAN estimates of incidence and mortality worldwide for 36 cancers in 185 countries. CA Cancer J. Clin..

[340] He M (2021). Protein convertase subtilisin/Kexin type 9 inhibits hepatocellular carcinoma growth by interacting with GSTP1 and suppressing the JNK signaling pathway. Cancer Biol. Med..

[341] Tao JH (2022). Reduced serum high-density lipoprotein cholesterol levels and aberrantly expressed cholesterol metabolism genes in colorectal cancer. World J. Clin. Cases.

[342] Wong CC (2022). The cholesterol uptake regulator PCSK9 promotes and is a therapeutic target in APC/KRAS-mutant colorectal cancer. Nat. Commun..

[343] Yang K (2021). Pro-protein convertase subtilisin/kexin type 9 promotes intestinal tumor development by activating Janus kinase 2/signal transducer and activator of transcription 3/SOCS3 signaling in Apc(Min/+) mice. Int J. Immunopathol. Pharm..

[344] Wang L (2022). PCSK9 promotes the progression and metastasis of colon cancer cells through regulation of EMT and PI3K/AKT signaling in tumor cells and phenotypic polarization of macrophages. J. Exp. Clin. Cancer Res..

[345] Wang R (2022). Inhibition of PCSK9 enhances the antitumor effect of PD-1 inhibitor in colorectal cancer by promoting the infiltration of CD8(+) T cells and the exclusion of Treg cells. Front. Immunol..

[346] Yuan J (2021). Potentiating CD8(+) T cell antitumor activity by inhibiting PCSK9 to promote LDLR-mediated TCR recycling and signaling. Protein Cell.

[347] Momtazi-Borojeni AA (2019). Potential anti-tumor effect of a nanoliposomal antiPCSK9 vaccine in mice bearing colorectal cancer. Arch. Med. Sci..

[348] Guo W (2022). Self-assembly of a multifunction DNA tetrahedron for effective delivery of aptamer PL1 and Pcsk9 siRNA potentiate immune checkpoint therapy for colorectal cancer. ACS Appl Mater. Interfaces.

[349] Marimuthu A (2013). SILAC-based quantitative proteomic analysis of gastric cancer secretome. Proteom. Clin. Appl..

[350] Zhang Z (2021). Identification of small proline-rich protein 1B (SPRR1B) as a prognostically predictive biomarker for lung adenocarcinoma by integrative bioinformatic analysis. Thorac. Cancer.

[351] Wang Y (2023). Construction of an endoplasmic reticulum stress-related signature in lung adenocarcinoma by comprehensive bioinformatics analysis. BMC Pulm. Med..

[352] Lopez-Alonso I (2022). Mechanical ventilation promotes lung tumour spread by modulation of cholesterol cell content. Eur. Respir. J..

[353] Suh JM (2021). Proprotein convertase subtilisin/kexin Type 9 is required for Ahnak-mediated metastasis of melanoma into lung epithelial cells. Neoplasia.

[354] Bonaventura A (2019). Serum PCSK9 levels at the second nivolumab cycle predict overall survival in elderly patients with NSCLC: a pilot study. Cancer Immunol. Immunother..

[355] Xie M (2022). Low baseline plasma PCSK9 level is associated with good clinical outcomes of immune checkpoint inhibitors in advanced non-small cell lung cancer. Thorac. Cancer.

[356] Gao X (2023). PCSK9 regulates the efficacy of immune checkpoint therapy in lung cancer. Front. Immunol..

[357] Wu Y (2020). Identification of the six-RNA-binding protein signature for prognosis prediction in bladder cancer. Front. Genet..

[358] Yang QC (2023). Targeting PCSK9 reduces cancer cell stemness and enhances antitumor immunity in head and neck cancer. iScience.

[359] Montero-Calle, A. et al. In-depth quantitative proteomics analysis revealed C1GALT1 depletion in ECC-1 cells mimics an aggressive endometrial cancer phenotype observed in cancer patients with low C1GALT1 expression. *Cell Oncol. (Dordr.)* **46**, 697–715 (2023). 10.1007/s13402-023-00778-w PMC10205863 36745330

[360] Jacome Sanz D (2021). Evaluating targeted therapies in ovarian cancer metabolism: novel role for pcsk9 and second generation mTOR inhibitors. Cancers (Basel).

[361] Sun L (2022). Associations of genetically proxied inhibition of HMG-CoA reductase, NPC1L1, and PCSK9 with breast cancer and prostate cancer. Breast Cancer Res.

[362] Fang S (2023). Association between genetically proxied PCSK9 inhibition and prostate cancer risk: A Mendelian randomisation study. PLoS Med..

[363] Gormley M (2021). Using genetic variants to evaluate the causal effect of cholesterol lowering on head and neck cancer risk: a Mendelian randomization study. PLoS Genet..

[364] Liu L (2021). Association between genetically proxied lipid-lowering drug targets and renal cell carcinoma: a mendelian randomization study. Front. Nutr..

[365] Ha CSR (2023). Proteomics biomarker discovery for individualized prevention of familial pancreatic cancer using statistical learning. PLoS ONE.

[366] Hanahan D (2022). Hallmarks of cancer: new dimensions. Cancer Discov..

[367] Xu X (2017). PCSK9 regulates apoptosis in human lung adenocarcinoma A549 cells via endoplasmic reticulum stress and mitochondrial signaling pathways. Exp. Ther. Med..

[368] Piao MX, Bai JW, Zhang PF, Zhang YZ (2015). PCSK9 regulates apoptosis in human neuroglioma u251 cells via mitochondrial signaling pathways. Int. J. Clin. Exp. Pathol..

[369] Bai J (2017). A retrospective study of NENs and miR-224 promotes apoptosis of BON-1 cells by targeting PCSK9 inhibition. Oncotarget.

[370] Alannan M (2022). Targeting PCSK9 in liver cancer cells triggers metabolic exhaustion and cell death by ferroptosis. Cells.

[371] Alannan M (2022). Rewiring lipid metabolism by targeting PCSK9 and HMGCR to treat liver cancer. Cancers (Basel).

[372] He M (2017). Actinidia chinensis Planch root extract inhibits cholesterol metabolism in hepatocellular carcinoma through upregulation of PCSK9. Oncotarget.

[373] Gan SS (2017). Inhibition of PCSK9 protects against radiation-induced damage of prostate cancer cells. Onco Targets Ther..

[374] Sun X (2012). Proprotein convertase subtilisin/kexin type 9 deficiency reduces melanoma metastasis in liver. Neoplasia.

[375] Gu Y (2023). PCSK9 facilitates melanoma pathogenesis via a network regulating tumor immunity. J. Exp. Clin. Cancer Res..

[376] Katsuki S (2022). Proprotein convertase subtilisin/kexin 9 (PCSK9) promotes macrophage activation via LDL receptor-independent mechanisms. Circ. Res..

[377] Mantovani A (2017). Tumour-associated macrophages as treatment targets in oncology. Nat. Rev. Clin. Oncol..

[378] Hu J (2022). PCSK9 suppresses M2-like tumor-associated macrophage polarization by regulating the secretion of OX40L from hepatocellular carcinoma cells. Immunol. Invest.

[379] Mach F (2020). 2019 ESC/EAS guidelines for the management of dyslipidaemias: lipid modification to reduce cardiovascular risk. Eur. Heart J..

[380] Catapano AL, Pirillo A, Norata GD (2020). New pharmacological approaches to target PCSK9. Curr. Atheroscler. Rep..

[381] Gaine SP, Quispe R, Patel J, Michos ED (2022). New strategies for lowering low density lipoprotein cholesterol for cardiovascular disease prevention. Curr. Cardiovasc Risk Rep..

[382] Ahamad S, Bhat SA (2022). Recent update on the development of PCSK9 inhibitors for hypercholesterolemia treatment. J. Med. Chem..

[383] Seidah NG (2019). Novel strategies to target proprotein convertase subtilisin kexin 9: beyond monoclonal antibodies. Cardiovasc. Res..

[384] Tombling BJ (2021). The emerging landscape of peptide-based inhibitors of PCSK9. Atherosclerosis.

[385] Coppinger C (2022). A comprehensive review of PCSK9 inhibitors. J. Cardiovasc. Pharm. Ther..

[386] Nishikido T, Ray KK (2018). Non-antibody approaches to proprotein convertase subtilisin kexin 9 inhibition: siRNA, antisense oligonucleotides, adnectins, vaccination, and new attempts at small-molecule inhibitors based on new discoveries. Front. Cardiovasc. Med..

[387] Rifai MA, Ballantyne CM (2021). PCSK9-targeted therapies: present and future approaches. Nat. Rev. Cardiol..

[388] Rothgangl T (2021). In vivo adenine base editing of PCSK9 in macaques reduces LDL cholesterol levels. Nat. Biotechnol..

[389] Wang L (2018). Meganuclease targeting of PCSK9 in macaque liver leads to stable reduction in serum cholesterol. Nat. Biotechnol..

[390] Wang L (2021). Long-term stable reduction of low-density lipoprotein in nonhuman primates following in vivo genome editing of PCSK9. Mol. Ther..

[391] Lintner NG (2017). Selective stalling of human translation through small-molecule engagement of the ribosome nascent chain. PLoS Biol..

[392] Zhang Y (2014). Identification of a small peptide that inhibits PCSK9 protein binding to the low density lipoprotein receptor. J. Biol. Chem..

[393] Ding Q (2014). Permanent alteration of PCSK9 with in vivo CRISPR-Cas9 genome editing. Circ. Res..

[394] Li Q (2021). In vivo PCSK9 gene editing using an all-in-one self-cleavage AAV-CRISPR system. Mol. Ther. Methods Clin. Dev..

[395] Jiang C (2017). A non-viral CRISPR/Cas9 delivery system for therapeutically targeting HBV DNA and pcsk9 in vivo. Cell Res..

[396] Musunuru K (2021). In vivo CRISPR base editing of PCSK9 durably lowers cholesterol in primates. Nature.

[397] Xu S, Luo S, Zhu Z, Xu J (2019). Small molecules as inhibitors of PCSK9: current status and future challenges. Eur. J. Med. Chem..

[398] Stein EA (2012). Effect of a monoclonal antibody to PCSK9 on LDL cholesterol. N. Engl. J. Med..

[399] Sullivan D (2012). Effect of a monoclonal antibody to PCSK9 on low-density lipoprotein cholesterol levels in statin-intolerant patients: the GAUSS randomized trial. JAMA.

[400] Chai M (2023). Efficacy and safety of tafolecimab in Chinese patients with heterozygous familial hypercholesterolemia: a randomized, double-blind, placebo-controlled phase 3 trial (CREDIT-2). BMC Med..

[401] Fitzgerald K (2017). A Highly Durable RNAi Therapeutic Inhibitor of PCSK9. N. Engl. J. Med..

[402] Hardy J (2021). A critical review of the efficacy and safety of Inclisiran. Am. J. Cardiovasc. Drugs.

[403] Yadav K, Sharma M, Ferdinand KC (2016). Proprotein convertase subtilisin/kexin type 9 (PCSK9) inhibitors: present perspectives and future horizons. Nutr. Metab. Cardiovasc Dis..

[404] Traber, G. M. & Yu, A. M. RNAi based therapeutics and novel RNA bioengineering technologies. *J. Pharmacol. Exp. Ther.* **384**, 133–154 (2022). 10.1124/jpet.122.001234 PMC9827509 35680378

[405] Keam SJ (2023). Tafolecimab: First Approval. Drugs.

[406] Qi L (2023). Tafolecimab in Chinese patients with hypercholesterolemia (CREDIT-4): a randomized, double-blind, placebo-controlled phase 3 trial. *JACC*. Asia.

[407] Grzesk G (2022). Safety of PCSK9 inhibitors. Biomed. Pharmacother..

[408] Winston-McPherson GN (2019). Discovery of 2,3’-diindolylmethanes as a novel class of PCSK9 modulators. Bioorg. Med. Chem. Lett..

[409] Qiao MQ (2023). Structure-activity relationship and biological evaluation of xanthine derivatives as PCSK9 inhibitors for the treatment of atherosclerosis. Eur. J. Med. Chem..

[410] Nagiec MM (2018). Novel tricyclic glycal-based TRIB1 inducers that reprogram LDL metabolism in hepatic cells. Medchemcomm.

[411] Petrilli WL (2020). From screening to targeted degradation: strategies for the discovery and optimization of small molecule ligands for PCSK9. Cell Chem. Biol..

[412] Evison BJ (2020). A small molecule inhibitor of PCSK9 that antagonizes LDL receptor binding via interaction with a cryptic PCSK9 binding groove. Bioorg. Med. Chem..

[413] Petersen DN (2016). A small-molecule anti-secretagogue of PCSK9 targets the 80S ribosome to inhibit PCSK9 protein translation. Cell Chem. Biol..

[414] Wang J (2022). Identification and evaluation of a lipid-lowering small compound in preclinical models and in a Phase I trial. Cell Metab..

[415] Adorni MP (2020). Naturally occurring PCSK9 inhibitors. Nutrients.

[416] Ahamad S, Mathew S, Khan WA, Mohanan K (2022). Development of small-molecule PCSK9 inhibitors for the treatment of hypercholesterolemia. Drug Discov. Today.

[417] Singh, S. et al. Natural proprotein convertase subtilisin/kexin type 9 inhibitors: a review. *Comb. Chem. High Throughput Screen* **26**, 2668–2678 (2023). 10.2174/1386207326666230627122630 37366365

[418] Nhoek P (2021). Sesquiterpenoids from the aerial parts of salvia plebeia with inhibitory activities on proprotein convertase subtilisin/kexin type 9 expression. J. Nat. Prod..

[419] Ahn J (2019). Prenylated flavonoids from the roots and rhizomes of sophora tonkinensis and their effects on the expression of inflammatory mediators and proprotein convertase subtilisin/kexin type 9. J. Nat. Prod..

[420] Li HH (2020). 23,24-Dihydrocucurbitacin B promotes lipid clearance by dual transcriptional regulation of LDLR and PCSK9. Acta Pharm. Sin..

[421] Chae HS (2021). Identification of neolignans with PCSK9 downregulatory and LDLR upregulatory activities from Penthorum chinense and the potential in cholesterol uptake by transcriptional regulation of LDLR via SREBP2. J. Ethnopharmacol..

[422] Abdelwahed KS (2020). Pseurotin A as a novel suppressor of hormone dependent breast cancer progression and recurrence by inhibiting PCSK9 secretion and interaction with LDL receptor. Pharm. Res..

[423] Wang D (2020). Ascorbic acid enhances low-density lipoprotein receptor expression by suppressing proprotein convertase subtilisin/kexin 9 expression. J. Biol. Chem..

[424] Sui GG, Xiao HB, Lu XY, Sun ZL (2018). Naringin activates AMPK resulting in altered expression of SREBPs, PCSK9, and LDLR to reduce body weight in obese C57BL/6J mice. J. Agric. Food Chem..

[425] Stucchi M (2016). Disrupting the PCSK9/LDLR protein-protein interaction by an imidazole-based minimalist peptidomimetic. Org. Biomol. Chem..

[426] Taechalertpaisarn J, Zhao B, Liang X, Burgess K (2018). Small molecule inhibitors of the PCSK9.LDLR interaction. J. Am. Chem. Soc..

[427] Brousseau ME (2022). Identification of a PCSK9-LDLR disruptor peptide with in vivo function. Cell Chem. Biol..

[428] Sabnis RW (2020). Novel cyclic tetramer compounds as PCSK9 inhibitors for treating metabolic disorders. ACS Med. Chem. Lett..

[429] Tombling BJ (2021). Increased Valency Improves Inhibitory Activity of Peptides Targeting Proprotein Convertase Subtilisin/Kexin Type 9 (PCSK9) Abstract. ChemBioChem.

[430] Kong W (2004). Berberine is a novel cholesterol-lowering drug working through a unique mechanism distinct from statins. Nat. Med..

[431] Schroeder CI (2014). Design and synthesis of truncated EGF-A peptides that restore LDL-R recycling in the presence of PCSK9 in vitro. Chem. Biol..

[432] Alleyne C (2020). Series of novel and highly potent cyclic peptide PCSK9 inhibitors derived from an mRNA display screen and optimized via structure-based design. J. Med Chem..

[433] Tucker TJ (2021). A series of novel, highly potent, and orally bioavailable next-generation tricyclic peptide PCSK9 inhibitors. J. Med. Chem..

[434] Iskandar SE, Bowers AA (2022). mRNA display reaches for the clinic with new PCSK9 inhibitor. ACS Med Chem. Lett..

[435] Johns DG (2023). Orally bioavailable macrocyclic peptide that inhibits binding of PCSK9 to the low density lipoprotein receptor. Circulation.

[436] Ballantyne CM (2023). Phase 2b randomized trial of the oral PCSK9 inhibitor MK-0616. J. Am. Coll. Cardiol..

[437] Zhang Y (2017). Discovery of a cryptic peptide-binding site on PCSK9 and design of antagonists. Nat. Struct. Mol. Biol..

[438] Kirchhofer D (2020). Regions of conformational flexibility in the proprotein convertase PCSK9 and design of antagonists for LDL cholesterol lowering. Biochem. Soc. Trans..

[439] Burdick DJ (2020). Design of organo-peptides as bipartite PCSK9 antagonists. ACS Chem. Biol..

[440] Oza PP, Kashfi K (2023). The evolving landscape of PCSK9 inhibition in cancer. Eur. J. Pharm..

[441] Singh A (2023). A comprehensive review on PCSK9 as mechanistic target approach in cancer therapy. Mini Rev. Med. Chem..

[442] Momtazi-Borojeni AA, Jaafari MR, Badiee A, Sahebkar A (2019). Long-term generation of antiPCSK9 antibody using a nanoliposome-based vaccine delivery system. Atherosclerosis.

[443] Momtazi-Borojeni AA (2020). Effects of immunisation against PCSK9 in mice bearing melanoma. Arch. Med. Sci..

[444] Momtazi-Borojeni AA (2019). Effects of immunization against PCSK9 in an experimental model of breast cancer. Arch. Med. Sci..

[445] Zeitlinger M (2021). A phase I study assessing the safety, tolerability, immunogenicity, and low-density lipoprotein cholesterol-lowering activity of immunotherapeutics targeting PCSK9. Eur. J. Clin. Pharm..

[446] Alshaer W (2021). siRNA: Mechanism of action, challenges, and therapeutic approaches. Eur. J. Pharm..

[447] Liu C (2022). PCSK9 inhibition: from current advances to evolving future. Cells.

[448] Mitchell T (2014). Pharmacologic profile of the Adnectin BMS-962476, a small protein biologic alternative to PCSK9 antibodies for low-density lipoprotein lowering. J. Pharm. Exp. Ther..

[449] Abdelwahed KS (2023). Pseurotin a validation as a metastatic castration-resistant prostate cancer recurrence-suppressing lead via PCSK9-LDLR axis modulation. Mar. Drugs.

[450] McGehee OC (2023). Towards developing novel prostate cancer recurrence suppressors: acute toxicity of pseurotin a, an orally active PCSK9 axis-targeting small-molecule in swiss albino mice. Molecules.

[451] Yamagata H (2023). Association of high proprotein convertase subtilisin/kexin type 9 antibody level with poor prognosis in patients with diabetes: a prospective study. Sci. Rep..

[452] Sabatine MS (2017). Evolocumab and clinical outcomes in patients with cardiovascular disease. N. Engl. J. Med..

[453] Sabatine MS (2017). Cardiovascular safety and efficacy of the PCSK9 inhibitor evolocumab in patients with and without diabetes and the effect of evolocumab on glycaemia and risk of new-onset diabetes: a prespecified analysis of the FOURIER randomised controlled trial. Lancet Diabetes Endocrinol..

[454] Wiviott SD (2020). Effect of evolocumab on type and size of subsequent myocardial infarction: a prespecified analysis of the FOURIER randomized clinical trial. JAMA Cardiol..

[455] Deedwania P (2021). Efficacy and safety of PCSK9 inhibition with evolocumab in reducing cardiovascular events in patients with metabolic syndrome receiving statin therapy: secondary analysis from the FOURIER randomized clinical trial. JAMA Cardiol..

[456] Gaba P (2023). Association between achieved low-density lipoprotein cholesterol levels and long-term cardiovascular and safety outcomes: an analysis of FOURIER-OLE. Circulation.

[457] Erviti J (2022). Restoring mortality data in the FOURIER cardiovascular outcomes trial of evolocumab in patients with cardiovascular disease: a reanalysis based on regulatory data. BMJ Open.

[458] Emerging Risk Factors C (2009). Lipoprotein(a) concentration and the risk of coronary heart disease, stroke, and nonvascular mortality. JAMA.

[459] Boffa MB, Koschinsky ML (2018). The journey towards understanding lipoprotein(a) and cardiovascular disease risk: are we there yet?. Curr. Opin. Lipido..

[460] Romagnuolo R (2015). Lipoprotein(a) catabolism is regulated by proprotein convertase subtilisin/kexin type 9 through the low density lipoprotein receptor. J. Biol. Chem..

[461] Romagnuolo R (2017). Roles of the low density lipoprotein receptor and related receptors in inhibition of lipoprotein(a) internalization by proprotein convertase subtilisin/kexin type 9. PLoS ONE.

[462] Blanchard V (2022). The size of apolipoprotein (a) is an independent determinant of the reduction in lipoprotein (a) induced by PCSK9 inhibitors. Cardiovasc. Res..

[463] Villard EF (2016). PCSK9 modulates the secretion but not the cellular uptake of Lipoprotein(a) ex vivo: an effect blunted by alirocumab. JACC Basic Transl. Sci..

[464] Schwartz GG (2018). Alirocumab and cardiovascular outcomes after acute coronary syndrome. N. Engl. J. Med..

[465] Jukema JW (2019). Alirocumab in patients with polyvascular disease and recent acute coronary syndrome: ODYSSEY OUTCOMES Trial. J. Am. Coll. Cardiol..

[466] Kosmas, C. E. et al. New and emerging lipid-modifying drugs to lower LDL cholesterol. *Drugs Context*. **10**, 10.7573/dic.2021-8-3 (2021). 10.7573/dic.2021-8-3 PMC8565402 34795777

[467] Stoekenbroek RM, Kallend D, Wijngaard PL, Kastelein JJ (2018). Inclisiran for the treatment of cardiovascular disease: the ORION clinical development program. Future Cardiol..

[468] Kallend D (2022). Pharmacokinetics and pharmacodynamics of inclisiran, a small interfering RNA therapy, in patients with hepatic impairment. J. Clin. Lipido..

[469] Wright RS (2020). Effects of renal impairment on the pharmacokinetics, efficacy, and safety of inclisiran: an analysis of the ORION-7 and ORION-1 studies. Mayo Clin. Proc..

[470] Kallend D (2022). An evaluation of a supratherapeutic dose of inclisiran on cardiac repolarization in healthy volunteers: A phase I, randomized study. Clin. Transl. Sci..

[471] Ray KK (2017). Inclisiran in Patients at High Cardiovascular Risk with Elevated LDL Cholesterol. N. Engl. J. Med..

[472] Ray KK (2018). Effect of an siRNA therapeutic targeting PCSK9 on atherogenic lipoproteins: prespecified secondary end points in ORION 1. Circulation.

[473] Ray KK (2023). Long-term efficacy and safety of inclisiran in patients with high cardiovascular risk and elevated LDL cholesterol (ORION-3): results from the 4-year open-label extension of the ORION-1 trial. Lancet Diabetes Endocrinol..

[474] Ray KK (2020). Two phase 3 trials of inclisiran in patients with elevated LDL cholesterol. N. Engl. J. Med..

[475] Raal FJ (2020). Inclisiran for the treatment of heterozygous familial hypercholesterolemia. N. Engl. J. Med..

[476] Khan SA, Naz A, Qamar Masood M, Shah R (2020). Meta-analysis of inclisiran for the treatment of hypercholesterolemia. Am. J. Cardiol..

[477] Wright RS (2021). Pooled patient-level analysis of inclisiran trials in patients with familial hypercholesterolemia or atherosclerosis. J. Am. Coll. Cardiol..

[478] Cicero, A. F. G. et al. Efficacy and safety of inclisiran a newly approved FDA drug: a systematic review and pooled analysis of available clinical studies. *Am. Heart J. Plus: Cardiol. Res. Practice* **13**, 100127 (2022). 10.1016/j.ahjo.2022.100127 PMC10978220 38560059

[479] Nishikido T (2023). Clinical potential of inclisiran for patients with a high risk of atherosclerotic cardiovascular disease. Cardiovasc. Diabetol..

[480] Hovingh GK (2020). Inclisiran durably lowers low-density lipoprotein cholesterol and proprotein convertase subtilisin/kexin type 9 expression in homozygous familial hypercholesterolemia: the ORION-2 pilot study. Circulation.

[481] Toth PP (2022). Network meta-analysis of randomized trials evaluating the comparative efficacy of lipid-lowering therapies added to maximally tolerated statins for the reduction of low-density lipoprotein cholesterol. J. Am. Heart Assoc..

[482] Banach M (2022). Personalized management of dyslipidemias in patients with diabetes-it is time for a new approach (2022). Cardiovasc. Diabetol..

[483] Visseren FLJ (2021). 2021 ESC Guidelines on cardiovascular disease prevention in clinical practice. Eur. Heart J..

[484] Ebenezer O, Comoglio P, Wong GK, Tuszynski JA (2023). Development of novel siRNA therapeutics: a review with a focus on inclisiran for the treatment of hypercholesterolemia. Int J. Mol. Sci..

[485] Scicchitano P (2021). Inclisiran in lipid management: a Literature overview and future perspectives. Biomed. Pharmacother..

[486] Cui Y (2021). A potential long-acting LDL-cholesterol-lowering pcsk9 monoclonal antibody: randomized, placebo-controlled phase 1 studies. *JACC*. Asia.

[487] Huo Y (2022). Abstract 10111: efficacy and safety of tafolecimab in Chinese patients with non-familial hypercholesterolemia (CREDIT-1): a 48-week randomized, double-blind, placebo-controlled phase 3 trial. Circulation.

[488] Gurgoze MT (2019). Adverse events associated with PCSK9 inhibitors: a real-world experience. Clin. Pharm. Ther..

[489] Robinson JG (2015). Efficacy and safety of alirocumab in reducing lipids and cardiovascular events. N. Engl. J. Med..

[490] Sabatine MS (2015). Efficacy and safety of evolocumab in reducing lipids and cardiovascular events. N. Engl. J. Med..

[491] Giugliano RP (2017). Design and rationale of the EBBINGHAUS trial: A phase 3, double-blind, placebo-controlled, multicenter study to assess the effect of evolocumab on cognitive function in patients with clinically evident cardiovascular disease and receiving statin background lipid-lowering therapy-A cognitive study of patients enrolled in the FOURIER trial. Clin. Cardiol..

[492] Ding L (2022). Musculoskeletal adverse events associated with PCSK9 inhibitors: disproportionality analysis of the FDA adverse event reporting system. Cardiovasc. Ther..

[493] Debruyne PR (2001). The role of bile acids in carcinogenesis. Mutat. Res..

[494] Rajpathak SN (2009). Statin therapy and risk of developing type 2 diabetes: a meta-analysis. Diabetes Care.

[495] Mbikay M (2010). PCSK9-deficient mice exhibit impaired glucose tolerance and pancreatic islet abnormalities. FEBS Lett..

[496] Colhoun HM (2016). No effect of PCSK9 inhibitor alirocumab on the incidence of diabetes in a pooled analysis from 10 ODYSSEY Phase 3 studies. Eur. Heart J..

[497] Gouni-Berthold I, Schwarz J, Berthold HK (2022). PCSK9 monoclonal antibodies: new developments and their relevance in a nucleic acid-based therapy era. Curr. Atheroscler. Rep..

[498] Seidah NG, Garcon D (2022). Expanding biology of PCSK9: roles in atherosclerosis and beyond. Curr. Atheroscler. Rep..

[499] Seidah NG (2017). The PCSK9 revolution and the potential of PCSK9-based therapies to reduce LDL-cholesterol. Glob. Cardiol. Sci. Pr..

[500] Katzmann JL, Gouni-Berthold I, Laufs U (2020). PCSK9 inhibition: insights from clinical trials and future prospects. Front. Physiol..

[501] Liu S (2023). PCSK9 attenuates efferocytosis in endothelial cells and promotes vascular aging. Theranostics.

[502] Masuda Y (2018). Generation and Characterization of a Novel Small Biologic Alternative to Proprotein Convertase Subtilisin/Kexin Type 9 (PCSK9) Antibodies DS-9001a Albumin Binding Domain–Fused Anticalin Protein. J. Pharmacol. Exp. Ther..

